# Hydrogen-Bond-Assisted Diels–Alder Kinetics or Self-Healing in Reversible Polymer Networks? A Combined Experimental and Theoretical Study

**DOI:** 10.3390/molecules27061961

**Published:** 2022-03-17

**Authors:** Jessica Mangialetto, Kiano Gorissen, Lise Vermeersch, Bruno Van Mele, Niko Van den Brande, Freija De Vleeschouwer

**Affiliations:** 1 Physical Chemistry and Polymer Science (FYSC), Vrije Universiteit Brussel (VUB), Pleinlaan 2, 1050 Brussels, Belgium; jessica.mangialetto@vub.be (J.M.); bruno.van.mele@vub.be (B.V.M.); niko.van.den.brande@vub.be (N.V.d.B.); 2 General Chemistry–Algemene Chemie (ALGC), Vrije Universiteit Brussel (VUB), Pleinlaan 2, 1050 Brussels, Belgium; kiano.gorissen@vub.be (K.G.); lise.vermeersch@vub.be (L.V.)

**Keywords:** hydrogen bonding, Diels–Alder catalysis, microcalorimetry, density functional theory

## Abstract

Diels–Alder (DA) cycloadditions in reversible polymer networks are important for designing sustainable materials with self-healing properties. In this study, the DA kinetics of hydroxyl-substituted bis- and tetrafunctional furans with bis- and tris-functional maleimides, both containing ether-functionalized spacers, is investigated by modelling two equilibria representing the *endo* and *exo* cycloadduct formation. Concretely, the potential catalysis of the DA reaction through hydrogen bonding between hydroxyl of the furans and carbonyl of the maleimides or ether of the spacers is experimentally and theoretically scrutinized. Initial reaction rates and forward DA rate constants are determined by microcalorimetry at 20 °C for a model series of reversible networks, extended with (i) a hydroxyl-free network and hydroxyl-free linear or branched systems, and (ii) polypropylene glycol additives, increasing the hydroxyl concentration. A computational density-functional theory study is carried out on the *endo* and *exo* cycloadditions of furan and maleimide derivatives, representative for the experimental ones, in the absence and presence of ethylene glycol as additive. Additionally, an ester-substituted furan was investigated as a hydroxyl-free system for comparison. Experiment and theory indicate that the catalytic effect of H-bonding is absent or very limited. While increased concentration of H-bonding could in theory catalyze the DA reaction, the experimental results rule out this supposition.

## 1. Introduction

**Figure 1:**
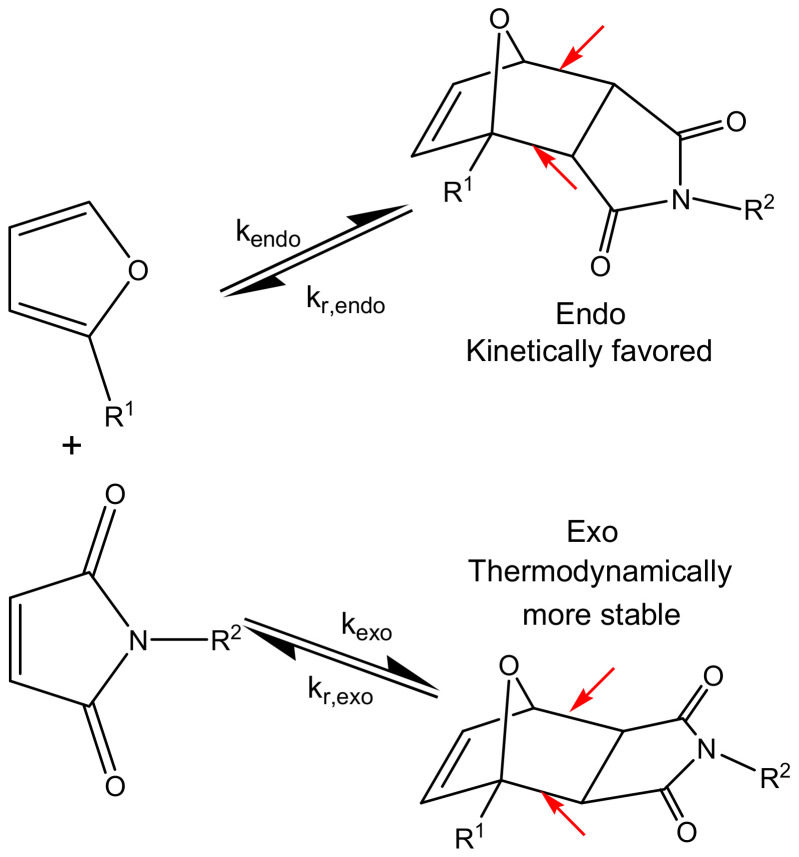
Furan-maleimide DA cycloadditions with formation of *endo* and *exo* stereoisomers. R^1^ and R^2^ represent flexible side groups with hydroxyl- and ether-functional groups. Reversible Diels–Alder bonds are indicated by red arrows. Adapted with permission from [9]. Copyright 2019 American Chemical Society.

Diels–Alder (DA) cycloadditions based on furan-maleimide chemistry, with formation of *endo* and *exo* stereoisomers according to two equilibria (see Figure 1), are important for developing reversible polymer networks by the introduction of reversible covalent bonds in the polymer backbone (indicated by arrows in Figure 1) [1,2,3,4,5,6,7,8,9,10,11].

In this way, self-healing polymer materials can be formed. In the case of damage, the reversible DA bonds are preferentially broken. This is a direct consequence of the DA equilibrium kinetics and the lower bond strength of the reversible DA bonds. However, they can be restored again, leading to the healing of the material with recovery of properties [4,6,7,10,12].

The DA equilibrium and the kinetics of forward and backward reaction are not only important for understanding the healing ability of the polymer material, but also for designing new self-healing materials with improved properties for various kinds of applications, such as protective thermoset coatings with sustainable barrier properties [3,13,14,15], elastomers for soft robotics [16,17], and 3D printing materials [18].

Recently, the kinetics and thermodynamics were experimentally determined for a series of reversible model networks of which the glass transition temperature and thermomechanical properties could be altered by varying the spacer length between the furan and maleimide DA reagents (see R^1^ and R^2^ flexible side groups in Figure 1) [8,9,11]. A mechanistic model as shown in Figure 1 was used, considering the equilibrium reactions between the furan and maleimide functional groups and the *endo* and *exo* DA cycloadducts. The main experimental kinetic and thermodynamic conclusions were [8,11]:

The formation of the *endo* cycloadduct has a lower activation energy than the *exo* cycloadduct and is faster at 298 K.

The retro DA reaction of the *exo* cycloadduct has a higher activation energy than the *endo* cycloadduct.

The *exo* cycloadduct is more stable than the *endo* cycloadduct.

The kinetic model that was used to determine the rates of the forward and retro Diels–Alder reactions in our model networks did not take into account any secondary, noncovalent interactions. Nonetheless, the spacers used in the model networks contain different concentrations of hydroxyl and ether functionalities (see Section 2.1. Materials), which might influence the kinetics of the model systems by hydrogen bonding (HB) interactions. Several studies have demonstrated that strong or bifunctional hydrogen bonds are capable of catalyzing the Diels–Alder cycloaddition reaction and may even alter the preference for *endo* versus *exo* [19,20,21,22,23].

In this paper, we try to elucidate whether hydrogen bonding influences the kinetics of our model networks with applications in self-healing materials, using a combined experimental and theoretical study. To this end, additional experimental data are provided on the effect of the concentrations of hydroxyl and ether functionalities on the initial reaction rate of the furan-maleimide DA cycloadditions at 20 °C. To further increase the hydroxyl concentration and measure its effect on the DA kinetics, different amounts of polypropylene glycol can be added to the networks. Moreover, hydroxyl-free systems and a network with ester groups, replacing the hydroxyl functionalities of the furan side group, are considered as well to exclude any effect of hydrogen bonding.

The objective of the theoretical study is to validate the experimental findings on kinetics and thermodynamics of these furan-maleimide reversible networks and to evaluate the potential catalytic effect of the HB interactions. To this end, a computational density-functional theory (DFT) study was carried out on the *endo* and *exo* cycloaddition reactions of model furan (Furan) and maleimide (Maleimide) derivatives as depicted in Figure 1, for which a reasonable cut-off of the spacers in the side groups R^1^ and R^2^ has been applied. As such, the R^1^ side group of Furan includes a hydroxyl functionality which can act as a hydrogen bond donor to a variety of sites, among which are the carbonyls of the maleimide’s main structure, the ring oxygen in furan and the maleimide’s and furan’s ether functionalities in the flexible side groups. Additionally, polypropylene glycol additives are modeled by inclusion of an ethylene glycol to the DA system. A detailed analysis is presented on how H-bonding interactions may affect the activation energy of both the forward and retro DA reactions, considering different conformations and making distinction between (1) H-bonding through the OH group of the furan reagent and (2) H-bonding via addition of a hydrogen bond donor as a third component. The analysis includes a preliminary study on the hydrogen bond strength and the favorability of orbital interactions by selected secondary interactions, since in literature it is generally accepted that catalysts reduce the energy barrier in part by a lowering of the HOMO-LUMO energy gap [24,25]. The DFT study is concluded by comparing the Furan-Maleimide results with the kinetics for the DA reaction between Maleimide and a hydroxyl-free furan molecule, in which the OH in Furan is replaced by an ester (OC(O)CH_3_) functional group.

## 2. Materials and Methods

### 2.1. Materials

**Figure 2:**
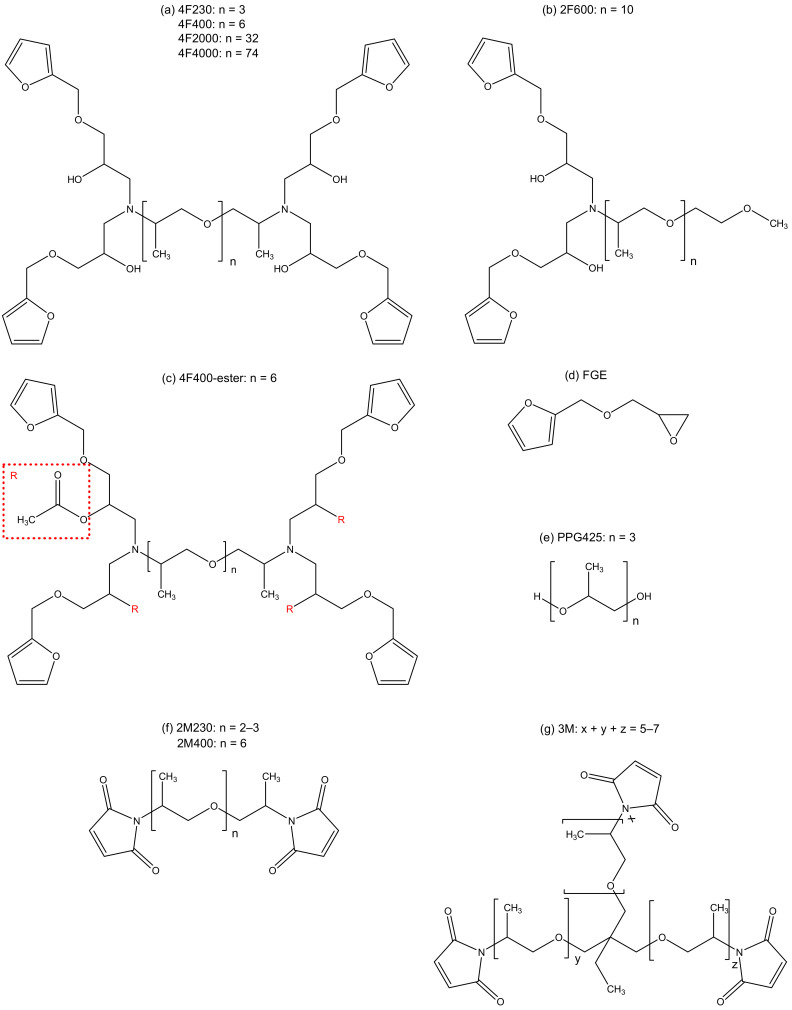
Furan and maleimide compounds for the kinetic study.

The furan and maleimide compounds used to measure the initial Diels–Alder reaction rates at 20 °C are depicted in Figure 2. Their nomenclature and synthesis are described in [9,11]. The synthesis of the modified 4F400-ester (OH free) is described in the Supplementary Materials. Polypropylene glycol of molar mass 425 (PPG425, Sigma Aldrich, Saint Louis, MO, USA) is also shown in Figure 2.

Fresh stoichiometric DA mixtures, indicated in Table 1, are prepared as described in [9]. The stoichiometric mixture of 4F400 and 2M400 is also prepared with different concentrations of PPG425 as additive, ranging from 8 wt% to 58 wt% of PPG425 in the mixture. In the case of 58 wt% of PPG425, the hydroxyl concentration is doubled compared to the hydroxyl concentration in the pure 4F400-2M400 mixture.

The average preparation time before entering the microcalorimeter is 5 min at an average temperature of 23 °C, which is 3 °C higher than the chosen microcalorimeter temperature. This preparation time at 23 °C is taken into account in the calculation of the experimental initial DA reaction rate (see Section 2.2. Methods).

### 2.2. Methods

#### 2.2.1. Microcalorimetry

A Thermal Activity Monitor TAM III from TA Instruments (New Castle, DE, USA) is used to follow the reaction kinetics of different DA mixtures by heat flow measurements. Isothermal measurements at 20 °C are performed on freshly prepared samples with a weight of around 2 g. All heat flows used are normalized against the sample mass and extrapolated to time = 0. For more details see the Supplementary Materials.

#### 2.2.2. Kinetic Calculations

A mechanistic model for the reaction kinetics is used that considers the equilibrium reactions between the compounds with furan and maleimide functional groups, as shown in Figure 2 and Table 1 (see Section 2.1. Materials), and the stereoisomeric *endo* and *exo* cycloadducts. The kinetics of the Diels–Alder and retro Diels–Alder reactions are described for both stereoisomers by their corresponding rate constants k_kin_(T) as shown in Figure 1. The mechanistic model leads to a set of differential equations for all components i:

(1) $$\frac{{\mathrm{dC}}_{i}}{\mathrm{dt}}=\overset{R}{\underset{j=1}{\sum }}{(v}_{j})$$

where C_i_ (mol·kg^−1^) is the concentration and dC_i_/dt (mol kg^−1^ s^−1^) the production rate of component i, R is the number of reactions involved (R = 2 or 4), v_j_ (mol kg^−1^ s^−1^) is the formation (or consumption) rate of component i in reaction j, which depends on the rate constant and the concentrations of the components involved in reaction j. The differential equations can be related to the measured reaction heat flow curves normalized against the sample weight through:

(2) $$\frac{{\mathrm{dq}}_{r}}{\mathrm{dt}}=\overset{N}{\underset{i=1}{\sum }}\left[\frac{{\mathrm{dC}}_{i}}{\mathrm{dt}}\;{∆}_{f}H_{i}^{0}\right]$$

where dq_r_/dt (W kg^−1^) is the experimental normalized heat flow, and N is the number of components i with formation enthalpy Δ_f_H_i_^0^ (kJ mol^−1^) (N = 4).

For each DA mixture of Table 1 (see Section 2.1. Materials), the normalized TAM heat flow after 60 min at 20 °C is corrected to time = 0 (see Section 2.2.1), using Equations (1) and (2), together with the concentration of the starting products (i.e., [F]_0_ for the furan and [M]_0_ for the maleimide compound), and the average preparation time (5 min) and temperature (23 °C). Optimized rate constants *k* with reaction enthalpies Δ_r_H^0^ for *endo* and *exo* adducts are used for these corrections (see Table S1) [11]. All normalized heat flows at time = 0, dq_r0_/dt, are converted to initial DA reaction rates, v_0_, using Equations (3) and (4):

(3) $$\frac{{\mathrm{dq}}_{r0}}{\mathrm{dt}}{=v}_{\mathrm{endo}}\;{∆}_{r}H_{\mathrm{endo}}^{0}{+v}_{\mathrm{exo}}\;{∆}_{r}H_{\mathrm{exo}}^{0}$$

v_0_ = v_endo_ + v_exo_ = (k_endo_ + k_exo_) [F]_0_ [M]_0_ = k_DA_ [F]_0_ [M]_0_ (4)

The initial DA reaction rates are further normalized against the initial concentrations of each system to obtain k_DA_ values at 20 °C for comparison.

#### 2.2.3. Density Functional Theory Calculations

All calculations were performed in the gas phase using density functional theory (DFT) as implemented in Gaussian16 [26]. Geometry optimizations and subsequent vibrational frequency calculations on all stationary points, i.e., reagents, reactant complexes, transition states and cycloadducts, were carried out at the M06-2X [27,28] level of theory. The Minnesota functional with 54% exact exchange was selected since it is widely used for Diels–Alder kinetics [29,30,31,32], and in particular the noncovalent catalysis of Diels–Alder reactions [33,34,35]. The integration grid was set to ultrafine. The zero-point vibrational energy correction and thermal corrections to the enthalpy and Gibbs free energy, at 298.15 K and 1 atm., were obtained from the harmonic vibrational frequency calculations. In addition, it was confirmed that each stationary point was indeed a minimum or first order saddle point (for TSs) on the potential energy surface. The full energy paths connecting all relevant stationary points were constructed via intrinsic reaction coordinate (IRC) calculations starting from the TSs, using the same level of theory. Next, refined energies were computed using M06-2X with the Grimme D3 dispersion correction [36] coupled to cc-pVDZ, cc-pVTZ and cc-pVQZ to allow for an extrapolation to the complete basis set (CBS) limit using the Feller three-point extrapolation [37,38], as such eliminating the basis set superposition error. Table S2 reports the comparison of our level of theory with the gold-standard and benchmark method CCSD(T)/CBS for the unsubstituted furan-maleimide *endo* and *exo* Diels–Alder reactions. Whereas the kinetics of the forward Diels–Alder reactions is reproduced very well by M06-2X, the DA adducts are understabilized due to the so-called delocalization error inherent to most density functional approximations [39]. Nevertheless, the sequence in reaction energies and retro DA barriers are expected to remain unaffected. In addition, orbital energies were taken at the M06-2X/cc-pVDZ level of theory. Finally, we would like to note that during our study focus was put on the energy differences between transition state structures and reactant complexes, and not on the barriers taken from the separate reactants, as this more closely represents the experimental settings of the self-healing polymer networks.

## 3. Results and Discussion

### 3.1. Experimental Study

Recently, the kinetics and thermodynamics for a model series of reversible networks based on DA cycloadditions were experimentally determined [11]. While the same furan-maleimide cycloaddition reactions were studied, the molecular architecture of the amorphous networks (and the corresponding glass transition temperature and thermomechanical properties) could be altered by using different spacer lengths (and thus concentrations of hydroxyl and ether groups) of the furan and maleimide compounds in the mixtures (see Figure 2 in Section 2.1. Materials). A mechanistic model with four rate constants as shown in Figure 1 was used, considering the equilibrium reactions between the furan and maleimide functional groups and the *endo* and *exo* DA cycloadducts. Non-isothermal microcalorimetric (normalized) heat flow data, measured from 20 °C to 90 °C, in combination with isothermal data at 55 °C, were used to fit the model parameters. One unique set of kinetic and thermodynamic equilibrium parameters was optimized in ref. [11] (see Table S1 in the Supplementary Materials), describing in a satisfactory way the reaction rates of the full set of reversible networks from low to high reaction conversions and for temperatures ranging from 20 °C to 90 °C, taking into account the effect of forward and backward DA reactions along the reaction path. Although all model networks contained different concentrations of hydroxyl and ether functionalities in their respective spacers, no extra reaction steps in the model were needed for an accurate description of all measured heat flow profiles.

#### 3.1.1. Model Series of Reversible Networks: Effect of Concentrations of Hydroxyl and Ether Functional Groups on Initial Reaction Rate and Forward DA Rate Constant

To further examine the potential influence of secondary H-bonding interactions on the furan-maleimide kinetics, an alternative experimental approach solely based on isothermally measured initial DA reaction rates (at reaction time t = 0) is followed in this paper.

For this purpose, the DA rates of different stoichiometric mixtures of furans and maleimides, giving rise to the model series of reversible networks, are experimentally determined by microcalorimetry in isothermal conditions at 20 °C. The measured rates are extrapolated to initial DA rates (v_0_) at reaction time t = 0, using the optimized kinetic parameter set of ref. [11] (see Table S1 and Section 2.2. Methods). The initial reaction rates v_0_ are further normalized against the starting concentrations of the furan and maleimide functional groups in the reaction mixtures. This ratio v_0_/([F]_0_ [M]_0_) at 20 °C is equal to the experimental forward DA rate constant k_DA_ at 20 °C, i.e., the rate constant for the formation of the sum of *endo* and *exo* cycloadducts without any interference of the retro DA (backward) reactions (see Equation (4) in Section 2.2.2. Kinetic calculations). The average experimental value of k_DA_ for this model series of DA reactions is <k_DA_> = 2.29 ± 0.24 10^−5^ kg (mol s)^−1^ (see Table S3 in the Supplementary Materials). A reliable and constant value of <k_DA_> is obtained with a limited standard deviation (SD = 0.24 10^−5^ kg (mol s)^−1^), certainly if slight potential errors in the stoichiometric mixing ratio of all independently prepared mixtures are taken into account (as a result of small uncertainties on the average functionalities of the furan and maleimide compounds and the fast mixing protocol to limit reaction conversion during preparation outside the analytical instrument) (see Section 2.1 Materials, Table S1, and Ref. [11]).

#### 3.1.2. Extended Series: Effect of Hydroxyl-free Systems and Increased Concentration of Hydroxyl Groups on Initial Reaction Rate and Forward DA Rate Constant

The model series of networks is further extended with a hydroxyl-free furan-maleimide network (based on 4F400-ester, see Supplementary Materials) and some hydroxyl-free linear or branched furan-maleimide DA systems (based on the monofunctional furan FGE). On the other hand, the hydroxyl concentration in the stoichiometric furan-maleimide mixtures is also increased by adding different amounts of PPG425 as a third component (see Section 2.1. Materials).

The DA rates of these additional stoichiometric mixtures of furans and maleimides (plus PPG425 additive) are experimentally determined by microcalorimetry in isothermal conditions at 20 °C and interpreted in the same way as discussed in Section 3.1.1. The average experimental value of k_DA_ (=v_0_/([F]_0_ [M]_0_) at 20 °C) for the extended series of DA reactions (model series of 3.1.1. plus OH-free systems and systems with PPG425) is <k_DA_> = 2.27 ± 0.21 10^−5^ kg (mol s)^−1^ (see Table S3). Very similar values of <k_DA_> and SD are obtained as for the model series in 3.1.1. The experimental <k_DA_> of this extended series is slightly lower than the value calculated with the optimized kinetic parameters from Table S1 (k_DA_ (*endo* plus *exo* at 20 °C) = 2.42 10^−5^ kg (mol s)^−1^). This slight deviation (6%) is acceptable in view of the extra experimental systems involved and the different experimental approaches (vide supra). Note also that a deviation of 6% on k_DA_ is within the SD limits, which can be expressed as equivalent to minor temperature changes of (less than) ± 1 °C around the average reaction temperature of 20 °C.

**Figure 3:**
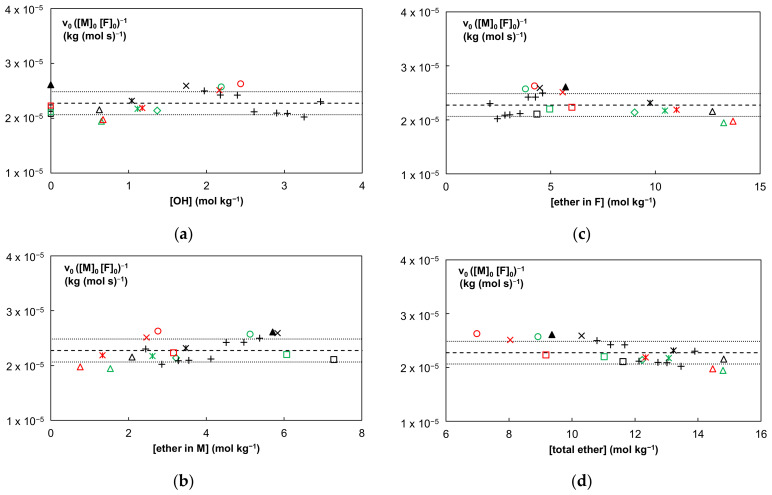
Forward DA rate constants k_DA_ at 20 °C (equal to initial DA reaction rates, v_0_, normalized against the starting concentrations of the furan and maleimide functional groups) as a function of the concentration in the mixtures of: (**a**) hydroxyl, (**b**) ether of the maleimide compounds, (**c**) ether of the furan compounds, and (**d**) total ether. Series of different maleimides with: FGE (□, □, □), 2F600 (◇), 4F230 (○, ○), 4F400 (✕, ✕) 4F400-ester (▲), 4F2000 (🞵, 🞵, 🞵), 4F4000 (△, △, △), 4F400-2M400 plus different amounts of PPG425 (+).

To examine the influence of the concentration of hydroxyl or ether functionalities on k_DA_ at 20 °C and to detect potential trends, all data of the extended series are presented in Figure 3. The ratio in the studied extended series = v_0_/([F]_0_ [M]_0_) at 20 °C is plotted as a function of the concentration of additional functional groups in these systems, i.e., OH (Figure 3a) and ether (Figure 3b–d). Nearly constant values for k_DA_ are observed in all plots, in agreement with the limited SD on <k_DA_>. Moreover, no obvious trends are found as a function of hydroxyl or ether concentration in the mixtures. The addition of extra OH-groups by the PPG425 additive, up to a doubled concentration in the mixture 4F400-2M400, also has no effect on the forward DA rate constant (see “+” symbols in Figure 3). A strong support for the absence of H-bond catalysis is the observation that the OH free linear or branched systems with FGE (FGE-2M230, FGE-2M400, and FGE-3M) and the OH free network (4F400-ester-2M400), in which *no* H-bonding is interfering, have a similar experimental value of k_DA_ (within the standard deviation of 0.21 10^−5^ kg (mol s)^−1^) and are in line with all data in Figure 3 (see symbols on y-axis of Figure 3a).

It can be concluded that the H-bonding catalytic effect of the hydroxyl groups of the furan compounds, interacting with ether groups of the spacers and carbonyl groups of the maleimides, on the DA reactivity seems to be absent or at most very limited in the studied extended series (even if the hydroxyl concentration is increased by the PPG425 additive). In the following section a computational study is performed to validate these experimental results.

### 3.2. Preliminary DFT Study

**Figure 4:**
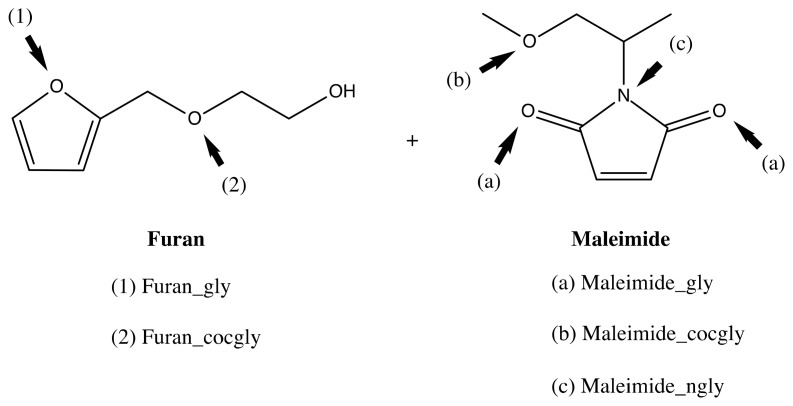
Coordination sites for the glycol group in Furan and Maleimide and their nomenclature.

In the preliminary study, the strength of hydrogen bonding (HB) that may occur in the formation of the reversible polymer network is estimated using the model compounds in combination with a glycol molecule serving as hydrogen bond donor. Both Furan and Maleimide contain a variety of HB acceptor sites, as displayed in Figure 4, with these being the furan’s ring oxygen and side-group’s ether functionality, and the maleimide’s carbonyls and ring nitrogen, as well as an ether functionality in the side group. Moreover, we also considered the intramolecular hydrogen bond that can be formed between the hydroxyl end group of the furan’s sidechain and the furan’s ring oxygen, termed in this paper as Furan_IHB. We found that hydrogen bonding interactions with Maleimide are stronger than with Furan. However, none of the interactions result in stable HB complexes. In addition, Furan with a linear side group is preferred over Furan with an intramolecular HB. A more comprehensive analysis can be found in the Supplementary Materials.

Next to the HB strength, a detailed look at the frontier molecular orbitals of the two compounds and their complexes with glycol is included, so that the possible favorable effect of HB coordination on the HOMO-LUMO energy gap can be scrutinized.

In Diels–Alder reactions, a diene, e.g., Furan, combines with a dienophile, e.g., Maleimide, to form a cycloadduct. DA reactions can be categorized into normal or inverse electron-demand reactions depending on which frontier orbital interactions are dominating: in the case of normal demand the HOMO of the diene strongly interacts with the LUMO of the dienophile, whereas for an inverse demand DA reaction, the diene’s LUMO and dienophile’s HOMO combination shows the smallest energy gap. It is also widely known that the orbital energy gap can be additionally reduced or increased by inclusion of electron-accepting or -donating substituents on the diene or dienophile. Moreover, DA reactions can be further accelerated by adding Lewis acids that can coordinate to one of the reagents or through suitable noncovalent interactions such as hydrogen bond donation [20,34,35,40,41,42]. For normal electron-demand DA reactions, this has been commonly attributed to a stabilization of the LUMO of the dienophile, as such reducing the orbital energy gap and lowering the activation energy to cycloaddition [24,25]. However, two very recent contributions by Vermeeren et al. [43,44] question this general assertion. The authors concluded that, even though Lewis acids may induce a considerable HOMO-LUMO gap reduction, the total orbital interactions between the reagents are not enhanced, and that the increased reactivity is due to diminished Pauli repulsion between the π-systems of the approaching reactants.

Nevertheless, we explored the impact of hydrogen bonding at the different coordination sites on the frontier orbital levels to probe possible catalytic activity through secondary interactions. Table S5 indicates that glycol interaction with the HB acceptor atoms in Furan stabilizes both the HOMO and LUMO levels compared to uncoordinated Furan, with the effect being slightly larger for the LUMO energy. When glycol forms a HB with one of the maleimide’s carbonyl groups, again a lowering of both of Maleimide’s frontier orbital energy levels is observed, but in this case it is less pronounced for the LUMO energy. Remark that in the case of Maleimide_gly, the occupied orbital that may combine with the diene’s LUMO is in fact the HOMO-3, since the HOMO to HOMO-2 orbitals are mainly located on the glycol and/or the maleimide’s side group (see Figure S1). A different situation is encountered for coordination to the ether’s oxygen of Maleimide_cocgly, for which a minor stabilization of the HOMO level is computed whereas the LUMO energy level is slightly destabilized.

Table 2 summarizes the HOMO-LUMO orbital energy differences between the LUMO of the maleimide and the HOMO of the furan, on the one hand, and maleimide’s HOMO and furan’s LUMO, on the other. From the tabulated values, it is clear that the furan-maleimide cycloaddition can be categorized as a normal electron-demand DA reaction. Focusing on the normal-demand orbital interactions, a HOMO-LUMO energy gap reduction is only registered for the Furan-Maleimide_gly combination, with a relatively modest value of 0.21 eV. The largest gaps are associated with the intramolecularly hydrogen bonded furan derivative, which showed the largest stabilization of the HOMO level.

**Figure 5:**
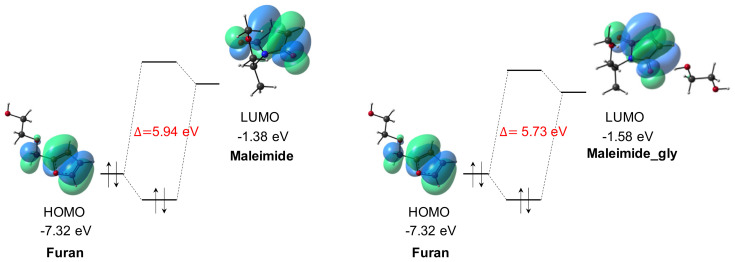
Orbital diagrams for the normal demand Diels–Alder reaction, with and without addition of glycol. Only the reduced orbital energy gap compared to the uncatalyzed DA reaction, combining Furan and Maleimide_gly, is depicted.

A simplified orbital diagram, showing the relevant frontier orbitals for the uncoordinated and glycol-coordinated maleimide in combination with Furan, is depicted in Figure 5. The HOMO and LUMO almost fully reside on the main furan and maleimide structure, respectively. Based on the computed orbital gaps, we conjecture that only HB coordination to the carbonyl groups in Maleimide may result in a catalytic effect, as should be reflected in a lower activation energy compared to the DA reaction without considering secondary interactions. In the next section, the impact of hydrogen bonding, with a hydroxyl group as HB donor, on the DA reaction profiles will be examined in detail for both *endo* and *exo* cycloaddition.

### 3.3. Influence of Hydrogen Bonding on Diels–Alder Energetics of Substituted Furan and Maleimide

**Figure 6:**
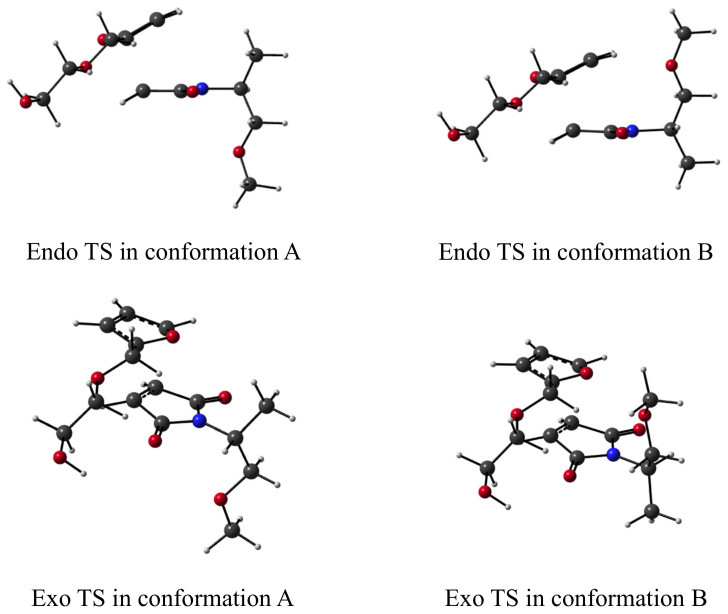
Transition state structures in conformation A and B for the *endo* and *exo* cycloaddition reaction.

We calculated the reaction profiles of the *endo* and *exo* cycloaddition reactions without and with hydrogen bonding interactions present. The energetics were computed at the same level of theory as the complexation energies in the Supplementary Materials. and are tabulated in Table 3 (enthalpy *H* at 298.15 K), Table S6 (internal energies at 0 K) and Table S7 (Gibbs free energies at 298.15 K). In all cases, two distinct conformations were considered, with the ether functionality of the maleimide’s side group pointing away (conformation A) or towards (conformation B) the furan molecule, as depicted in Figure 6.

When no secondary interactions are present, the lowest enthalpy of activation $∆H^{‡}$ is found for the formation of the *endo* cycloadduct in conformation B, with a value of 18.6 kcal mol^−1^. Even though *endo* conformation B is slightly more stable for the reactant complex and adduct compared to conformation A, the transition state of *endo* B is even lower in energy, as such yielding the lowest barriers for both the forward and reverse DA reactions. The $∆H^{‡}$ values for the *exo* DA reactions in conformations A and B are distinctly higher than their respective *endo* reactions, by 0.6 to 1.2 kcal mol^−1^ for the forward DA and 1.5 to 1.9 kcal mol^−1^ for the retro DA. Please note that when the reaction profiles for *exo* cyclization in conformations A and B are compared, it is found that conformation B results in lower enthalpies (with respect to the separate reagents) for the reactant complex and transition state structures but not for the *exo* adduct. When entropic contributions are also added, the stationary point structures in conformation A are energetically less destabilized than those in conformation B. However, this does not impact the trends we found for the activation and reaction enthalpies, which still indicate that *endo* cycloaddition is kinetically favored over *exo* cyclization (with also here a preference for conformation B) and that the *exo* products are more stable than their *endo* equivalents.

In a next step, we investigated the possibility of hydrogen bonding between the two reagents, in which the hydroxyl functionality in the furan’s side group is interacting with one of the carbonyl groups of the maleimide. For both *endo* and *exo* cycloaddition in conformations A and B, reaction profiles were obtained, which are denoted as M[C=O] + F[-OH] in Table 3, Table S6 and S7. The stabilizing effect of the hydrogen bonds is more pronounced in the *endo* than in the *exo* reactant complexes, but similar for the A and B conformations. In the transition state structures, the hydrogen bonding is around 1–2 kcal mol^−1^ more stabilizing than in the RCs. The different TS conformations are visualized in Figure S4. Please note that the magnitude of the energy lowering for the different stationary points does not correlate with the measured hydrogen bond lengths (-C=O…HO-) (Table 4), indicating that steric interactions also play a role.

Hence, for both *endo* and *exo* conformations, the hydrogen bonding gives rise to a reduction in the activation enthalpies by 0.8 to 1.7 kcal mol^−1^. The lowest barriers are found for conformation B, again with a difference of around 1 kcal mol^−1^ in favor of *endo* cyclization. The barriers for the *exo* retro DA reactions remain the same and reduce by ~1.5 kcal mol^−1^ for the *endo* retro DA (conformations A and B). When the Gibbs free energy profiles (Table S7) are considered, 4 reactions show a quantifiable reduction in barrier compared to the situation without HB: forward *endo* and *exo* conformation A and the retro *endo* DA reactions. Generalizing over both conformations, a minor to no measurable catalytic effect for the forward *endo* and *exo* cycloadditions and retro *exo* DA reactions is observed; theoretically, only the reverse DA reaction for *endo* adducts may be accelerated by hydrogen bonding, by a factor of 18 at most.

#### 3.3.1. Influence of Hydrogen Bonding of Additive on Diels–Alder Energetics of Substituted Furan and Maleimide

Besides this intermolecular hydrogen bonding between the reagents, we also examined the effect of possible hydrogen bond donation of polypropylene glycol (PPG) additives to one of the reagents. This type of HB was modeled by adding an ethane-1,2-diol or ethylene glycol molecule to the elementary DA reactions. Both Furan and Maleimide bear two types of hydrogen bond acceptor positions. Based on our orbital interaction study, only HB with the maleimide’s carbonyl groups is anticipated to induce a reduction of the energy barriers of the forward DA reaction, whereas coordination to the ring oxygen of furan is expected to increase the DA barriers.

First, Furan’s ring oxygen is considered, denoted as F[(O)] + glycol in Table 3. The enthalpy barriers at 25 °C for the forward and retro DA reactions are in almost all cases considerably larger than their respective “uncatalyzed” reactions. Only for the forward *exo* conformation B reaction is a similar activation enthalpy found. The same trends apply for the Gibbs free energetics. At first sight, it seems that coordination of glycol to Furan’s oxygen has a more adverse impact on the *endo* reactions compared to the *exo* reactions. This can be associated with an additional hydrogen bond formed between the second hydroxyl of glycol and the OH functionality of Furan and is only present in the reactant complexes. We therefore also computed the *endo* reaction paths using 2-methoxyethanol as a possible catalyst. The results are listed in Table S8. The energy barriers reduce by approximately 1.5 kcal mol^−1^ and are therefore more in line with what is observed for the *exo* reactions.

The second coordination site we examined is one of the carbonyl groups in Maleimide, listed as M[C=O] + glycol, for which the orbital diagrams conjecture a potential catalytic effect. Based on the enthalpy values in Table 3, hydrogen bonding causes a clear decrease in forward and retro DA reaction barriers for both *endo* and *exo*. This decrease is maintained upon inclusion of entropy. The barriers are ±1 kcal mol^−1^ lower than when the hydrogen bond arises from the actively involved furan reactant, except for forward *endo* conformation A for which a smaller decrease is distinguished and the retro *endo* conformation B which has a nearly identical barrier.

Finally, the ether groups part of Furan (F[-O-] + glycol) or Maleimide’s sidechain (M[-O-] + glycol) were also examined. In the case of Furan coordination, the $∆H^{‡}$ and $∆G^{‡}$ values are similar or slightly larger than for the “uncatalyzed” equivalents, both for the forward and retro cycloadditions. Looking at Maleimide’s ether hydrogen bonding, a ~1.5 kcal mol^−1^ lowering of the Gibbs free activation energy (but not for $∆H^{‡}$) is found for the forward *endo* and *exo* conformation B cycloadditions. For *endo*, this can be partially explained by increased steric repulsion due to the proximity of the furan reactant and the glycol-coordinated side group of the maleimide, as can be witnessed by the less stabilized reactant complex (RC) for this reaction though less so for the corresponding TS.

#### 3.3.2. Influence of Ester Substitution on Diels–Alder Energetics of Substituted Furan and Maleimide

To further elucidate the influence of hydrogen bonding on the experimental reaction rates, additional computational work was carried out in which the hydroxyl groups near the furans were replaced by ester functions, as such excluding any HB formation within the network. Table 5 lists the enthalpies H, computed at 298.15 K, for all stationary points along the reaction path of the [4+2] cycloaddition between Maleimide and Furan, but having an ester replacing the hydroxyl functionality. Again, the separate reagents are taken as reference. The 0K and Gibbs free energy (298.15 K) results are collected in Table S9.

Comparing the enthalpies in Table 5 to the first four entries of Table 3 (no HB), the ester-containing reactant complexes and TS structures are 0.6 to 1.8 kcal mol^−1^ less stable than their hydroxyl equivalents. The differences are even more pronounced for the products. However, the forward reaction barriers (TS − RC) are almost identical, whereas the retro DA barriers are on average about 2 kcal mol^−1^ lower. More importantly, these discrepancies nearly completely disappear for the Gibbs free energy values. Therefore, if the Maleimide-Furan DA reactions are not catalyzed through hydrogen bonding interactions (for the cases without and in the presence of PPG additive), then their reaction rates should match (within experimental deviations) with those obtained for the hydroxyl-free reactions. This is indeed demonstrated in Figure 3 of the experimental study, described in Section 3.1.

We can conclude that for all cases the *endo* formation remains kinetically favored, whereas the *exo* formation is thermodynamically favored, which is in agreement with our experimental findings. The discussed data indicate that, in theory, hydrogen bonding through functional groups present in the network polymers can act as a catalyst for both the forward and retro Diels–Alder reactions, if the maleimide’s carbonyl or possibly nearby ether groups are involved and with hydroxyl groups of furan or PPG as the possible hydrogen bond donors.

To allow for a more direct comparison with the experimental data in this study, rough estimates of the second-order rate constants were determined based on the computed Gibbs free energy profiles in Table S7 and compared to the experimental measurements listed in Table S3. First, the rate constants at 20 °C of all *endo* and *exo* cycloadditions in Table S7, i.e., without and with hydrogen bonding (furan-based or glycol-based), were calculated using the Eyring–Polanyi equation from transition state theory (Table S10). Next, the rate constants were weighted via the Boltzmann populations of the reactant complexes for three different scenarios: (1) not accounting for hydrogen bonding, (2) considering hydrogen bonding between the two reagents, Furan and Maleimide, (3) including for hydrogen bonding via glycol additives as well. Moreover, the weighted rate constant of the DA reaction between Maleimide and the OH-free, ester-substituted furan (Table S11) was estimated starting from the energetics in Table S9. The total DA reaction rate constants were taken as the sum of the rate constants obtained for *endo* and *exo* adduct formation and are listed in Table S12 together with the experimental second-order rate constants k_DA_ of the corresponding experimental systems.

Table 6 contains the predicted vs. the experimentally measured relative DA rate constants, with the average of the Model Series taken as the reference. A first observation is that possible hydrogen bonding between Furan and Maleimide does not affect the total rate constant. The rate constant for the ester-functionalized Furan-Maleimide cycloaddition is +/−1.5 times larger than for the ester system, for which no change was experimentally measured (cf. 4F400-ester-2M400). Note, however, that this relates to a Gibbs free activation energy difference of merely 0.3 kcal mol^−1^, which can be due to different errors as a result of the level of theory. Finally, the influence of the addition of glycol on the initial rate was evaluated. Theoretically, a non-negligible rate enhancement of a factor of 3.5 is seen, which is not reflected in the experimental data of 4F400-2M400 with PPG425 against the Model Series (average values).

In summary, hydrogen bonding via addition of a HB donor to the DA system can in theory catalyze the reaction to some degree if the majority of the OH groups, being from the furans’ side groups or glycols, are coordinated to sites that result in a lowering of the Gibbs free energy of activation. However, the experimental results clearly rule out this supposition. It is indeed very unlikely that every Diels–Alder reaction taking place during the formation of the reversible network will be catalyzed, mainly because the hydroxyl groups can interact with a range of different sites of which (not so nearby) ether functionalities in the spacers are the most abundant (cf. Figure 3) and which have no catalytic activity.

## 4. Conclusions

In this work, experimental measurements on the Diels–Alder kinetics of reversible polymer networks and a complementary computational DFT study on model systems were combined to shed light on the role that hydrogen bonding can play in accelerating forward and retro Diels–Alder reactions. It can be concluded from the calculations that for all cases the *endo* formation remains kinetically favored, whereas the *exo* formation is thermodynamically favored, in agreement with all experimental findings. Experiment and theory further indicate that the catalytic effect of H-bonding, resulting from interactions between hydroxyl-substituted furans with carbonyl of the maleimides or ether groups of the spacers, is very limited (or even absent) in the reversible networks studied. This is confirmed by the hydroxyl-free systems, which give rise to similar initial Diels–Alder reaction rates and forward Diels–Alder rate constants. While an increased concentration of H-bonding by the addition of hydroxyl containing additives could theoretically catalyze the Diels–Alder reaction, the experimental results rule out this supposition. A viable explanation is that hydroxyl groups can coordinate to a range of different sites, of which (not so nearby) ether groups of the spacers are the most abundant; however, with no catalytic activity.

It cannot be excluded that other configurations of Diels–Alder furan-maleimide cycloadditions, with other substitutions and higher concentrations of hydroxyl functionalities, might show a catalytic effect of H-bonding. Moreover, it should be emphasized that hydrogen bond-assisted Diels–Alder kinetics, as treated in this work, and hydrogen bond-assisted rate of self-healing are two distinct aspects to consider in self-healing reversible networks based on DA cycloadditions. While hydrogen bonding has no (or only minimal) effect on the Diels–Alder kinetics, as demonstrated in this paper, these secondary interactions might increase the rate of the self-healing process substantially. Indeed, damage recovery was accelerated in dual-dynamic polymer networks (with reversible Diels−Alder bonds *and* hydrogen bonding sites), compared to single-dynamic polymer networks (with only reversible Diels−Alder bonds) because of the faster dynamics of the hydrogen bonding/debonding equilibrium, as recently proven for reversible networks with similar Diels–Alder kinetics in [15].

The experimental and computational results justify the kinetic model used for the DA systems in this study and previous papers [8,9,10,11,15], which is based on two parallel equilibrium reactions for the *endo* and *exo* cycloadducts as shown in Figure 1, without any additional reaction steps for secondary interactions.

## Figures and Tables

**Table 1:** Stoichiometric DA mixtures for the kinetic study. The symbols and colors used for the mixtures are indicated.

Compound	2M230	2M400	3M
FGE	□	□	□
2F600			◇
4F230	○		○
4F400	✕	✕	
4F2000	🞵	🞵	🞵
4F4000	△	△	△
4F400 + PPG425		+	
4F400-ester		▲	

**Table 2:** Frontier orbital energy differences for the normal and inverse demand Diels–Alder reactions between a furan and a maleimide system. Values are given in eV.

Furan	Maleimide	Normal Demand	Inverse Demand
		M(L) − F(H)	F(L) − M(H)
Furan	Maleimide	5.94	10.42
Furan_IHB	Maleimide	6.52	9.76
Furan_gly	Maleimide	6.22	10.06
Furan_cocgly	Maleimide	6.12	10.18
Furan	Maleimide_gly	5.73	10.84
Furan_IHB	Maleimide_gly	6.31	10.18
Furan	Maleimide_cocgly	6.07	10.48

**Table 3:** The energetics of the Diels–Alder reactions using enthalpies *H* at 298.15 K, with and without addition of ethylene glycol. Hydrogen bond (HB) is formed with Furan (F) or Maleimide (M) (C=O: carbonyl; -OH: hydroxyl; (O): ring oxygen; -O-: ether). Values are relative to the separate reagents and given in kcal mol^−1^. R1_gly: reactant complex between first reactant and glycol; RC: reactant complex of R1_gly with second reactant R2; TS: transition state; P_gly: product complex with glycol; P: product.

HB?	Endo/Exo	R1_gly + R2	RC	TS	P_gly	P (+ gly)	TS − RC	TS − P(_gly)
no	Endo ^a^	-	−7.6	11.9	-	−14.9	19.4	26.7
	Endo ^b^	-	−8.4	10.3	-	−15.3	18.6	25.6
	Exo ^a^	-	−8.4	11.6	-	−17.0	20.0	28.6
	Exo ^b^	-	−9.6	10.2	-	−16.9	19.8	27.1
M[C=O] + F[-OH]	Endo ^a^	-	−11.8	6.5	-	−18.8	18.4	25.3
	Endo ^b^	-	−13.9	3.9	-	−20.4	17.8	24.2
	Exo ^a^	-	−11.9	7.2	-	−21.3	19.1	28.5
	Exo ^b^	-	−13.5	5.4	-	−21.7	18.9	27.1
F[(O)] + glycol	Endo ^a^	−7.8 ^d^	−15.8 ^d^	7.7	−20.7	−14.5	23.6 ^d^	28.4
(Furan_gly)	Endo ^b^	−7.5 ^d^	−17.0 ^d^	6.1	−21.1	−14.8	23.2 ^d^	27.2
	Exo ^a^	−5.4	−13.0	8.7	−22.6	−16.4	21.7	31.4
	Exo ^b,c^	−4.7	−14.8	4.8	−23.1	−15.9	19.6	28.0
F[-O-] + glycol	Endo ^a^	−5.9	−13.9	5.6	−21.9	−14.9	19.5	27.5
(Furan_cocgly)	Endo ^b^	−5.9	−16.5	3.8	−22.7	−15.3	20.4	26.5
	Exo ^a^	−5.4	−15.0	5.2	−23.4	−17.0	20.1	28.5
	Exo ^b^	−5.4	−16.6	4.5	−22.8	−16.4	21.1	27.2
M[C=O] + glycol	Endo ^a^	−6.7	−15.2	2.7	−22.3	−14.9	18.0	25.0
(Maleimide_gly)	Endo ^b^	−5.8	−15.8	1.4	−22.5	−15.3	17.1	23.9
	Exo ^a^	−7.1	−15.2	3.0	−24.5	−16.9	18.2	27.5
	Exo ^b^	−5.7	−16.2	1.9	−24.2	−16.9	18.1	26.1
M[-O-] + glycol	Endo ^a^	−8.0	−15.5	3.7	−24.0	−14.9	19.3	27.8
(Maleimide_cocgly)	Endo ^b^	−9.1	−14.7	3.7	−22.3	−15.3	18.4	26.0
	Exo ^a^	−8.3	−16.7	3.1	−26.5	−16.9	19.7	29.5
	Exo ^b^	−8.3	−16.8	2.4	−25.2	−16.4	19.2	27.6

^a^ Conformation 1 with ether functionality of the maleimide substituent pointing away from Furan; ^b^ conformation 2 with ether functionality of the maleimide substituent pointing in the direction of Furan; ^c^ the glycol’s hydroxyl group is forming a hydrogen bond with the furan’s ring oxygen and the maleimide sidechain’s ether group; ^d^ extra HB with other hydroxyl functionality of glycol.

**Table 4:** The hydrogen bond (HB) length measured in the stationary points of the M[C=O] + F[-OH] reaction. Values are given in Å. RC: reactant complex; TS: transition state; P: product.

System	RC	TS	P
Endo ^a^	1.923	1.873	1.904
Endo ^b^	1.913	1.877	1.901
Exo ^a^	1.874	1.855	1.861
Exo ^b^	1.859	1.815	1.826

^a^ Conformation A with ether functionality of the maleimide’s substituent pointing away from Furan; ^b^ conformation B with ether functionality of the maleimide’s substituent pointing in the direction of Furan.

**Table 5:** The energetics of the Diels–Alder reactions between Maleimide and ester-functionalized furan (with ester group instead of hydroxyl functionality) using enthalpies *H* at 298.15 K. Values are relative to the separate reagents and given in kcal mol^−1^. RC: reactant complex; TS: transition state; P: product.

HB?	Endo/Exo	RC	TS	P	TS − RC	TS − P
no	Endo ^a^	−7.0	12.7	−11.7	19.6	24.3
	Endo ^b^	−6.8	11.4	−12.2	18.2	23.6
	Exo ^a^	−7.3	13.1	−13.3	20.3	26.4
	Exo ^b^	−8.1	12.0	−13.4	20.1	25.4

^a^ Conformation A with ether functionality of the maleimide’s substituent pointing away from Furan; ^b^ conformation B with ether functionality of the maleimide’s substituent pointing in the direction of Furan.

**Table 6:** Experimental versus computed relative total Diels–Alder reaction rate constants k_DA_ at 20 °C, using k_DA_ of the average of the Model Series experimental systems or the “No HB” computational system as a reference. Difference in computed Δ*G*^‡^ with respect to the “No HB” system is given in kcal mol^−1^.

Exp. System	Rel. k_DA_	Comp. System	Rel. k_DA_	Diff. in Comp. Δ*G*^‡^
		No HB	1.0	
<Model Series>	1.0	Poss. HB from Furan	1.0	0.0
<4F400-2M400 + PPG425>	1.0	Glycol additive	3.5	−0.7
4F400-ester-2M400	1.1	Ester	0.7	+0.3
<FGE> (OH free)	1.0			

## Data Availability

The geometry of all structures has been included in the Supplementary Materials. Data of microcalorimetry measurements, as well as calculated forward DA rate constants, are stored on the institute (VUB) sharepoint and are available from the authors upon request.

## Supplementary Materials

The following supporting information can be downloaded at: https://www.mdpi.com/article/10.3390/molecules27061961/s1, Synthesis of modified 4F400, Microcalorimetry experiments method, Experimental kinetic parameters and rate constants (Table S1), Benchmark of level of theory (Table S2, [45]), Experimental forward DA rate constants at 20 °C (Table S3), Preliminary study on hydrogen bond strength (Figures S1 and S2, Table S4, [46,47,48]), HOMO and LUMO energies of furan and maleimide and their complexes with glycol (Table S5), Visualization of the highest occupied orbitals (Figure S3), Energetics of the Diels–Alder reactions at 0 K (Table S6) and 298.15 K (Table S7), Energetics of the *endo* Diels–Alder reactions at 0 K with addition of 2-methoxyethanol (Table S8), Transition state structures for the uncoordinated and hydrogen bond-assisted reactions (Figure S4), Energetics of the Diels–Alder reactions between Maleimide and OH-free furan (Table S9), Computed kinetics of the Diels–Alder reactions between Maleimide and Furan (Table S10), Computed kinetics of the Diels–Alder reactions between Maleimide and OH-free furan (Table S11), Experimental versus computed Diels–Alder rate constants k_DA_ at 20 °C (Table S12), Cartesian coordinates.

## Sample Availability

Samples of the compounds with structures shown in Figure 2 are available from the authors.
