# Supplementary material for: Hydrogen-Bond-Assisted Diels–Alder Kinetics or Self-Healing in Reversible Polymer Networks? A Combined Experimental and Theoretical Study
Source: Molecules. 2022 Mar 17;27(6):1961. doi: 10.3390/molecules27061961 (PMC8951177; doi:10.3390/molecules27061961)
Supplement: Supplementary file 1 [file molecules-27-01961-s001.zip › molecules-1607139-supplementary.pdf]

Supplementary Information

Hydrogen-Bond-Assisted Diels-Alder Kinetics or Self-Healing in Reversible Polymer Networks? A Combined Experimental and Theoretical Study

Jessica Mangialetto[1], Kiano Gorissen[2], Lise Vermeersch[2], Bruno Van Mele[1], Niko Van den Brande[1], Freija De Vleeschouwer[2*]

[1] Physical Chemistry and Polymer Science (FYSC), Vrije Universiteit Brussel (VUB), Pleinlaan 2, 1050 Brussels ; Jessica.Mangialetto@vub.be (J.M.), Bruno.Van.Mele@vub.be (B.V.M), Niko.Van.den.Brande@vub.be (N.V.d.B)
[2] General Chemistry - Algemene Chemie (ALGC), Vrije Universiteit Brussel (VUB), Pleinlaan 2, 1050 Brussels ; Kiano.Gorissen@vub.be (K.G.), Lise.Vermeersch@vub.be (L.V.), Freija.De.Vleeschouwer@vub.be (F.D.V)
* Correspondence: Freija.De.Vleeschouwer@vub.be

Table of Contents

Synthesis of 4F400-ester 4

Microcalorimetry 4

Table S1. Optimized kinetic parameters and rate constants k with reaction enthalpies $\Delta_r H^0$ for *endo* and *exo* adducts, and average functionalities for furan and maleimide compounds, according to ref. [11]. 5

Table S2. Benchmarking level of theory: comparison between M06-2X-D3/CBS and gold-standard CCSD(T)/CBS[a], both taken at M06-2X/cc-pVDZ optimized geometries, for the unsubstituted furan-maleimide Diels-Alder reaction for *endo* and *exo*. Electronic energies and Gibbs free energies at 298.15K are given in kcal mol^{-1}. Values are reported using the separate reactants as energy reference. . 6

Table S3. Forward DA rate constants (in 10^{-5} kg (mol s)$^{-1}$) at 20 °C (equal to initial experimental DA rates (v_0), normalized against initial furan ($[F]_0$) and maleimide ($[M]_0$) concentrations) for the different furan-maleimide systems of model series and extended series. 7

Preliminary study: hydrogen bond strength 8

Figure S1. Computed hydrogen-bonding complexes between maleimide acceptor sites, (a) and (b), or furan acceptor sites, (c) and (d), and a glycol molecule. In addition, a furan conformation with intramolecular hydrogen bond (e) was found. 8

Figure S2. Noncovalent interaction surfaces (isovalue reduced density gradient = 0.5 a.u.) plotted by NCIPLOT; the blue colored regions indicate hydrogen-bonding interactions, whereas the green colored regions represent weak Van der Waals type interactions, such as dispersion. 9

Table S4. Hydrogen bond length (in Å) and complexation energies at 0 K (ΔEcomplex), complexation enthalpies at 298.15K (ΔHcomplex) and complexation Gibbs free energies at 298.15K (ΔGcomplex) for glycol coordination to furan and maleimide, indicating the hydrogen bond strength. ΔE, ΔH and ΔG values are given in kcal mol^{-1}, ΔS values in cal mol^{-1} K^{-1} 9

Table S5. HOMO and LUMO energies of furan and maleimide and their complexes with glycol. Values are given in eV. Orbital differences are relative to the respective uncoordinated molecules. ... 10

Figure S3. Visualization of the highest occupied orbitals, HOMO to HOMO − 3, of **Maleimide_gly**. 10

Table S6. The energetics of the Diels-Alder reactions at 0 K, with and without addition of ethylene glycol. Hydrogen bond (HB) is formed with **Furan** (F) or **Maleimide** (M) (C=O: carbonyl; -OH: hydroxyl; (O): ring oxygen; -O-: ether). Values are relative to the separate reagents and given in kcal mol^{-1}. R1_gly: reactant complex between first reactant and glycol; RC: reactant complex of R1_gly with second reactant R2; TS: transition state; P_gly: product complex with glycol; P: product. 11

Table S7. The Gibbs free energetics of the Diels-Alder reactions at 298.15 K, with and without addition of ethylene glycol. Hydrogen bond (HB) is formed with **Furan** (F) or **Maleimide** (M) (C=O: carbonyl; -OH: hydroxyl; (O): ring oxygen; -O-: ether). Values are relative to the separate reagents and given in kcal mol^{-1}. R1_gly: reactant complex between first reactant and glycol; RC: reactant complex of R1_gly with second reactant R2; TS: transition state; P_gly: product complex with glycol; P: product. 12

Table S8. The energetics of the *endo* Diels-Alder reactions at 0 K (E) and 298.15 K (H and G), with and without addition of 2-methoxyethanol (cat). Hydrogen bond (HB) is formed with ring oxygen of **Furan**. Values are relative to the separate reagents and given in kcal mol^{-1}. R1_gly: reactant complex between first reactant and cat; RC: reactant complex of R1_gly with second reactant R2; TS: transition state; P_gly: product complex with cat; P: product. 13

Figure S4. Transition state structures for the uncoordinated and hydrogen-bond-assisted reactions, the latter originating from the furan's hydroxyl functionality and one of the maleimide's carbonyl groups. 14

Table S9. The energetics of the Diels-Alder reactions between **Maleimide** and ester-functionalized furan (with ester group instead of hydroxyl functionality) at 0 K (*E*) and 298.15 K (*G*). Values are relative to the separate reagents and given in kcal mol^{-1}. RC: reactant complex; TS: transition state; P: product. 15

Table S10. The computed kinetics of the Diels-Alder reactions between **Maleimide** and **Furan.** ΔG values (RC, TS, TS – RC) are given in kcal mol^{-1}. Rate constants at 20°C in s^{-1} (at the standard concentration of 1 mol L^{-1}) are computed via the Eyring-Polanyi equation from Transition State Theory. The rate constants of the individual reactions are then weighted via the Boltzmann population of the reactant complexes. RC: reactan complex; TS: transition state; P: product. 16

Table S11. The computed kinetics of the Diels-Alder reactions between **Maleimide** and ester-functionalized furan (with ester group instead of hydroxyl functionality). ΔG values (RC, TS, TS – RC) are given in kcal mol^{-1}. Rate constants at 20°C in s^{-1} (at the standard concentration of 1 mol L^{-1}) are computed via the Eyring-Polanyi equation from Transition State Theory. The rate constants of the individual reactions are then weighted via the Boltzmann population of the reactant complexes. RC: reactant complex; TS: transition state; P: product. 17

Table S12. Experimental[a] versus computed[b] Diels-Alder rate constants k_{DA} at 20°C. Exp. k_{DA} in kg mol^{-1} s^{-1}; comp. k_{DA} in s^{-1} (L mol^{-1}). Difference in associated ΔG ‡ given in kcal mol^{-1}. 18

Cartesian coordinates 19

Synthesis of 4F400-ester

Modified 4F400 without OH (4F400-ester) was synthesized by reacting 4F400 (1 equiv, 5mmol, 1.30 g) with acetic anhydride (2 equiv, 10 mmol, 0.94 mL) in dry dichloromethane (DCM, 10 mL) with pyridine (2.5 equiv, 12.5 mmol, 1.00 mL) and 4-dimethylaminopyridine (DMAP, 0.01 mol%, 0.0305 g). After reaction, while stirring overnight, 30 mL of DCM was added in combination with aqueous Na_2CO_3. The organic phase was extracted (3 times), washed with water (3 times) and dried over $MgSO_4$. The solution was then filtered and the solvent evaporated in a rotary evaporator.

Microcalorimetry

The temperature accuracy of the TAM is ± 0.1 K with a heat flow signal precision of ± 200 nW and a baseline drift of less than 250 nW over 24 h in isothermal conditions. Before lowering the 4 ml sample ampoule and the reference ampoule in the microcalorimeter cell, a thermal equilibration step of 15 minutes is needed. An additional time of 45 minutes in the microcalorimeter cell is needed to ensure reliable heat flows at 20 °C to follow the reaction kinetics of each DA system. The experimental TAM heat flow values are extrapolated to time = 0 at 20 °C, by calculating the effect of reaction conversion in the preceding 60 min at 20 °C and the average preparation time of 5 min of the fresh mixtures at room temperature (around 23 °C) by means of the optimized kinetics and thermodynamics of the DA systems of ref. [11] (see Table S1). As the reaction rates of all systems at 20 °C is low, this correction is limited to 12.5 % on average. All normalized heat flows at time = 0, dq_{r0}/dt, are linked to the initial DA reaction rates, v_0, according to equations (3) and (4) (see 2.2.2. Kinetic calculations).

Table S1. Optimized kinetic parameters and rate constants *k* with reaction enthalpies $\Delta_r H^0$ for *endo* and *exo* adducts, and average functionalities for furan and maleimide compounds, according to ref. [11].

**Kinetic/thermodynamic parameters**	**Endo adduct**[11]	**Kinetic/thermodynamic parameters**	**Exo adduct**[11]
$\ln(A_{DA,endo})$ (kg mol^{-1} s^{-1})	13.4	$\ln(A_{DA,exo})$ (kg mol^{-1} s^{-1})	14.7
$E_{DA,endo}$ (kJ mol^{-1})	59.4	$E_{DA,exo}$ (kJ mol^{-1})	64.6
$\ln(k_{DA,endo})$ (kg mol^{-1} s^{-1}) at 293 K	-11.0	$\ln(k_{DA,exo})$ (kg mol^{-1} s^{-1}) at 293 K	-11.8
$\ln(A_{rDA,endo})$ (s^{-1})	30.9	$\ln(A_{rDA,exo})$ (s^{-1})	31.4
$E_{rDA,endo}$ (kJ mol^{-1})	113.1	$E_{rDA,exo}$ (kJ mol^{-1})	123.6
$\ln(k_{rDA,endo})$ (s^{-1}) at 293 K	-15.5	$\ln(k_{rDA,exo})$ (s^{-1}) at 293 K	-19.3
$\Delta_r H^0_{endo}$ (kJ mol^{-1})	-53.7	$\Delta_r H^0_{exo}$ (kJ mol^{-1})	-59.0
$\Delta_r S^0_{endo}$ (J mol^{-1} K^{-1})	-145.2	$\Delta_r S^0_{exo}$ (J mol^{-1} K^{-1})	-138.9

**Average functionalities (MMeq in g mol^{-1})**	**Optimized set**[11]
f(FGE)	1.00 (154)
f(2F600)	1.87 (518)
f(4F230)	4.00 (213)
f(4F400)	3.75 (261)
f(4F400-OH free)	3.75 (284)
f(4F2000)	3.70 (675)
f(4F4000)	3.70 (1331)
f(2M230)	2.10 (183)
f(2M400)	1.95 (308)
f(3M)	2.65 (249)

Table S2. Benchmarking level of theory: comparison between M06-2X-D3/CBS and gold-standard CCSD(T)/CBS[a], both taken at M06-2X/cc-pVDZ optimized geometries, for the unsubstituted furan-maleimide Diels-Alder reaction for *endo* and *exo*. Electronic energies and Gibbs free energies at 298.15K are given in kcal mol^{-1}. Values are reported using the separate reactants as energy reference.

**Level of theory**	$\Delta E^{\ddagger}_{el}$ *endo*	$\Delta_r E_{el}$ *endo*	$\Delta G^{\ddagger}$ *endo*	$\Delta_r G$ *endo*	$\Delta E^{\ddagger}_{el}$ *exo*	$\Delta_r E_{el}$ *exo*	$\Delta G^{\ddagger}$ *exo*	$\Delta_r G$ *exo*
M06-2X-D3/CBS	13.7	-13.9	28.2	3.6	14.1	-15.9	28.6	1.5
CCSD(T)/CBS	13.7	-17.1	28.2	0.3	14.1	-19.2	28.6	-1.8

[a] Extrapolation to the complete basis set was obtained using the principles of focal point analysis (FPA), more specifically FPA-QZ (see reference Hajgató, B.; Deleuze, M.S.; Tozer, D.J.; De Proft, F. A benchmark theoretical study of the electron affinities of benzene and linear acenes. *J. Chem. Phys*. **2008**, *129*, 084308 and references therein)

Table S3. Forward DA rate constants (in 10^{-5} kg (mol s)$^{-1}$) at 20 °C (equal to initial experimental DA rates (v_0), normalized against initial furan ($[F]_0$) and maleimide ($[M]_0$) concentrations) for the different furan-maleimide systems of model series and extended series.

**DA systems**	Model series	Extended series
2F600-3M	2.14	2.14
4F230-2M230	2.63	2.63
4F230-3M	2.57	2.57
4F400-2M230	2.51	2.51
4F400-2M400	2.59	2.59
4F2000-2M230	2.19	2.19
4F2000-2M400	2.32	2.32
4F2000-3M	2.17	2.17
4F4000-2M230	1.97	1.97
4F4000-2M400	2.16	2.16
4F4000-3M	1.94	1.94
FGE-2M230 (OH free)		2.23
FGE-2M400 (OH free)		2.11
FGE-3M (OH free)		2.20
4F400-ester-2M400		2.61
4F400-2M400 + PPG425 ([OH] x 1.125)		2.50
4F400-2M400 + PPG425 ([OH] x 1.25)		2.42
4F400-2M400 + PPG425 ([OH] x 1.375)		2.42
4F400-2M400 + PPG425 ([OH] x 1.5)		2.12
4F400-2M400 + PPG425 ([OH] x 1.675)		2.10
4F400-2M400 + PPG425 ([OH] x 1.75)		2.02
4F400-2M400 + PPG425 ([OH] x 1.875)		2.09
4F400-2M400 + PPG425 ([OH] x 2)		2.30
Average value $\langle k_{DA} \rangle$	2.29 ± 0.24	2.27 ± 0.21

Preliminary study: hydrogen bond strength

The hydrogen bond strength is computed at the M06-2X-D3/CBS//M06-2X/cc-pVDZ level of theory as the complexation energy for the furan or the maleimide interacting with a glycol molecule. The structures of the different complexes are displayed in Figure S1. Note that coordination to the nitrogen atom of maleimide did not lead to an energetically stable HB complex.

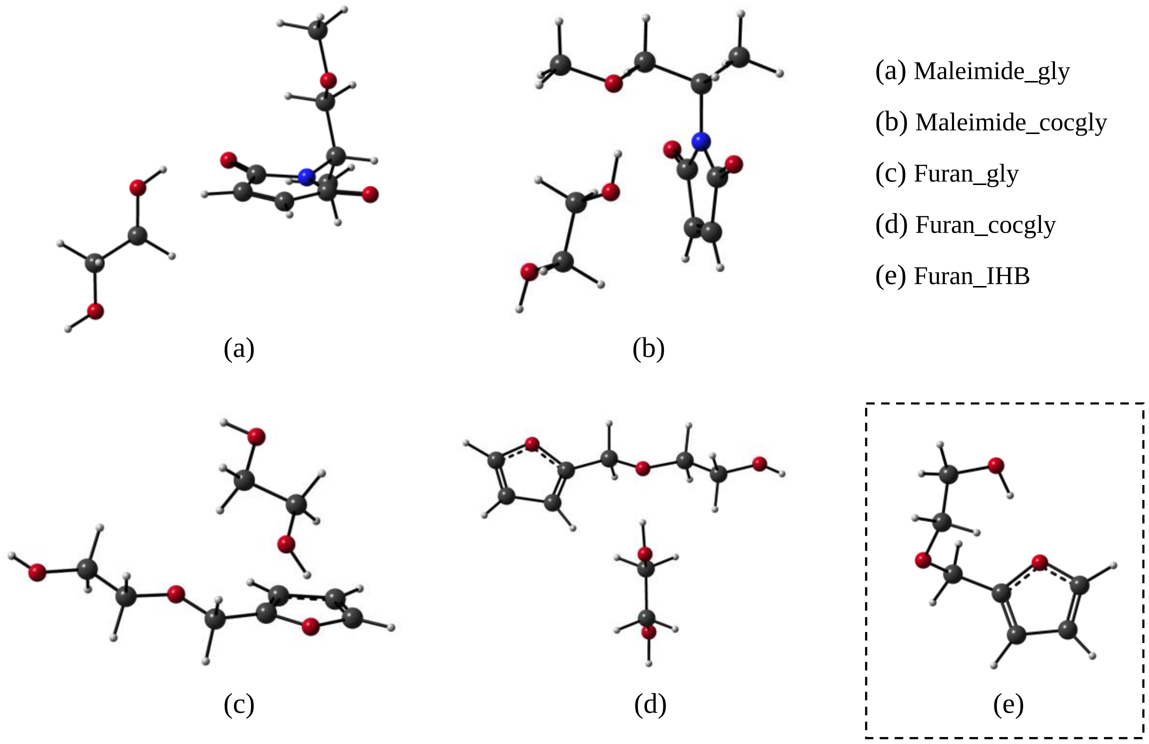

Figure S1. Computed hydrogen-bonding complexes between maleimide acceptor sites, (a) and (b), or furan acceptor sites, (c) and (d), and a glycol molecule. In addition, a furan conformation with intramolecular hydrogen bond (e) was found.

The resulting complexation values are listed in Table S4. At 0K, the strongest hydrogen bond is formed with the ether functionality of the maleimide, **Maleimide_cocgly**, which is about 2-2.5 kcal mol^{-1} more stable than **Furan_cocgly** and **Maleimide_gly**. The weakest interaction is found for complexation at the furan's ring oxygen. These results are maintained when considering the enthalpy at 298.15K. However, in gas-phase conditions at 298.15K, the negative entropic contributions prevent the complexes from spontaneously forming, as demonstrated by the positive $\Delta G_{complex}$ values. Hydrogen-bonding with the maleimide's carbonyl is now of similar strength as with the ether functional group in the maleimide's sidechain. Already in 1997, it was established that the furan aromatic oxygen is a considerably weaker HB acceptor than the oxygens in ether and carbonyl groups, which is consistent with our results [1]. Nevertheless, there is a significant discrepancy between the hydrogen-bonding interaction energy for **Maleimide_cocgly** and **Furan_cocgly**, both having an ether oxygen as HB acceptor atom. We therefore plotted all attractive noncovalent interactions present in each of the complexes using NCIPLOT [2,3] As shown in Figure S2, several dispersion interactions, indicated by the green colored regions, are present besides the stronger HB interaction displayed as a blue surface. However, the plot for **Maleimide_cocgly** additionally shows a distinct n-π interaction between the lone pair of the ether's oxygen and the π-cloud of the maleimide's heterocycle, which may explain the difference in complexation energy.

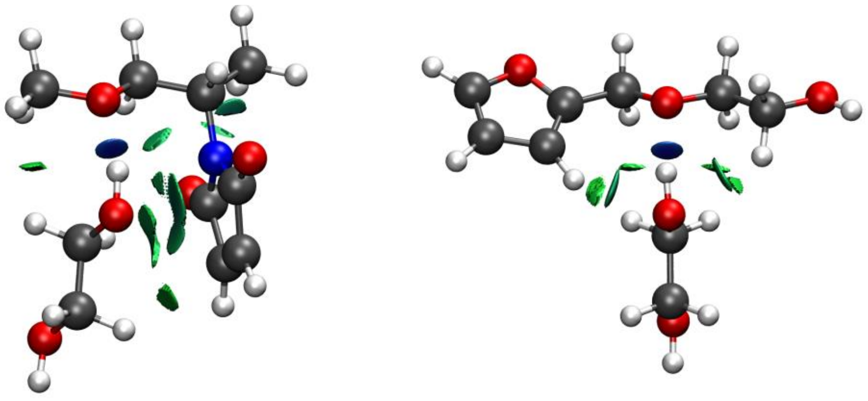

Maleimide_cocgly Furan_cocgly

Figure S2. Noncovalent interaction surfaces (isovalue reduced density gradient = 0.5 a.u.) plotted by NCIPLOT; the blue colored regions indicate hydrogen-bonding interactions, whereas the green colored regions represent weak Van der Waals type interactions, such as dispersion.

Finally, we note that the internal hydrogen bond that can be formed between the furan's hydroxyl group and its ring oxygen is not more stable than the furan conformation with a linear sidechain, especially when entropic contributions are considered as well (cf. positive ΔG value).

Table S4. Hydrogen bond length (in Å) and complexation energies at 0 K ($\Delta E_{\text{complex}}$), complexation enthalpies at 298.15K ($\Delta H_{\text{complex}}$) and complexation Gibbs free energies at 298.15K ($\Delta G_{\text{complex}}$) for glycol coordination to furan and maleimide, indicating the hydrogen bond strength. ΔE, ΔH and ΔG values are given in kcal mol^{-1}, ΔS values in cal mol^{-1} K^{-1}

Complex	HB length	$\Delta E_{complex}$	$\Delta H_{complex}$	$\Delta S_{complex}$	$\Delta G_{complex}$
Maleimide_gly	1.914	-6.1	-5.7	-31.9	3.8
Maleimide_cocgly	1.870	-8.3	-8.3	-40.9	3.9
Furan_gly	2.078	-5.1	-4.8	-37.9	6.5
Furan_cocgly	1.891	-5.9	-5.8	-36.9	5.2
Furan_IHB[1]	2.039	0.5	0.2	-3.4	1.2

[1] The intramolecular hydrogen bond strength of **Furan_IHB** was estimated as $\Delta E = E_{\text{Furan\_IHB}} - E_{\text{Furan}}$.

References:

[1] Nobeli, I.; Price, S.L.; Lommerse, J.P.M.; Taylor, R. Hydrogen bonding properties of oxygen and nitrogen acceptors in aromatic heterocycles. *J. Comput. Chem.* **1997**, *18*, 2060–2074, doi:10.1002/(SICI)1096-987X(199712)18:16<2060::AID-JCC10>3.0.CO;2-S.

[2] Johnson, E.R.; Keinan, S.; Mori-Sánchez, P.; Contreras-García, J.; Cohen, A.J.; Yang, W. Revealing noncovalent interactions. *J. Am. Chem. Soc.* **2010**, *132*, 6498–6506, doi:10.1021/ja100936w.

[3] Contreras-garcía, J.; Johnson, E.R.; Keinan, S.; Chaudret, R.; Piquemal, J.; Beratan, D.N.; Yang, W. NCIPLOT: A Program for Plotting Noncovalent Interaction Regions. *J. Chem. Theory Comput.* **2011**, *7*, 625–632, doi:10.1021/ct100641a.

Table S5. HOMO and LUMO energies of furan and maleimide and their complexes with glycol. Values are given in eV. Orbital differences are relative to the respective uncoordinated molecules.

System	ε_{HOMO}	ε_{LUMO}	$\Delta\varepsilon_{HOMO}$	$\Delta\varepsilon_{LUMO}$
Furan	-7.32	1.62		
Furan_IHB	-7.89	0.96	-0.58	-0.66
Furan_gly	-7.60	1.26	-0.28	-0.36
Furan_cocgly	-7.49	1.38	-0.17	-0.24
Maleimide	-8.80	-1.38		
Maleimide_gly	-9.22[1]	-1.58	-0.42	-0.21
Maleimide_cocgly	-8.86	-1.24	-0.06	0.13

[1] The occupied orbital involved in the formation of cycloadduct is the HOMO-3 energy level.

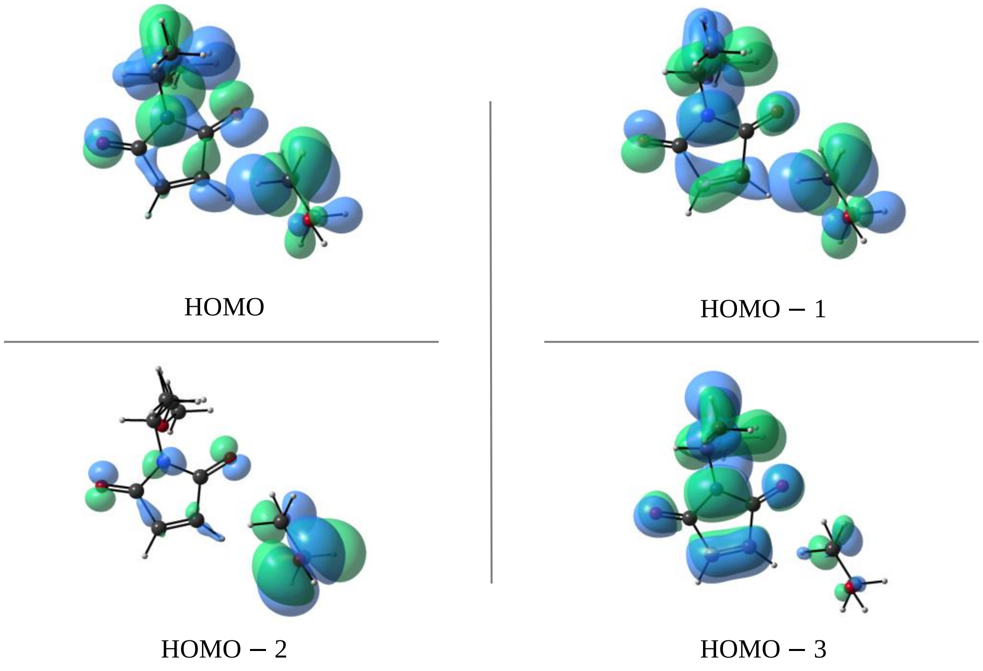

Figure S3. Visualization of the highest occupied orbitals, HOMO to HOMO − 3, of **Maleimide_gly**.

Table S6. The energetics of the Diels-Alder reactions at 0 K, with and without addition of ethylene glycol. Hydrogen bond (HB) is formed with **Furan** (F) or **Maleimide** (M) (C=O: carbonyl; -OH: hydroxyl; (O): ring oxygen; -O-: ether). Values are relative to the separate reagents and given in kcal mol^{-1}. R1_gly: reactant complex between first reactant and glycol; RC: reactant complex of R1_gly with second reactant R2; TS: transition state; P_gly: product complex with glycol; P: product.

HB?	Endo/Exo	R1_gly + R2	RC	TS	P_gly	P (+ gly)	TS - RC	TS - P(_gly)
no	Endo[a]	-	-7.7	12.5	-	-13.9	20.2	26.4
	Endo[b]	-	-8.4	11.1	-	-14.3	19.5	25.4
	Exo[a]	-	-8.6	12.5	-	-15.9	21.0	28.4
	Exo[b]	-	-9.5	11.2	-	-15.6	20.7	26.8
M[C=O] + F[-OH]	Endo[a]	-	-11.3	7.9	-	-17.2	29.2	25.1
	Endo[b]	-	-13.4	5.4	-	-18.5	18.9	24.0
	Exo[a]	-	-11.8	8.4	-	-19.8	20.2	28.2
	Exo[b]	-	-13.1	6.9	-	-19.9	20.0	26.8
F[(O)] + glycol	Endo[a]	-7.4[d]	-15.9[d]	7.8	-20.1	-13.6	23.7[d]	27.9
	Endo[b]	-7.1[d]	-17.1[d]	6.4	-20.5	-13.8	23.4[d]	26.9
	Exo[a]	-5.7	-13.6	9.2	-21.8	-15.4	22.8	31.0
	Exo[b,c]	-4.9	-15.1	5.5	-22.2	-14.6	20.5	27.6
F[-O-] + glycol	Endo[a]	-6.1	-14.6	5.9	-21.3	-13.9	20.5	27.2
	Endo[b]	-6.1	-16.9	4.2	-22.1	-14.3	21.1	26.3
	Exo[a]	-5.5	-15.5	5.7	-22.6	-15.9	21.2	28.2
	Exo[b]	-5.5	-16.8	5.0	-21.9	-15.2	21.8	26.9
M[C=O] + glycol	Endo[a]	-6.8	-15.8	3.0	-21.7	-13.9	18.8	24.7
	Endo[b]	-6.3	-16.1	1.8	-21.8	-14.3	17.9	23.6
	Exo[a]	-7.3	-15.8	3.4	-23.8	-15.8	19.2	27.2
	Exo[b]	-6.1	-16.5	2.5	-23.3	-15.6	19.1	25.8
M[-O-] + glycol	Endo[a]	-8.2	-15.9	4.2	-23.2	-13.9	20.1	27.4
	Endo[b]	-9.2	-14.9	4.2	-21.5	-14.3	19.1	25.6
	Exo[a]	-8.5	-17.1	3.7	-25.5	-15.8	20.8	29.2
	Exo[b]	-8.5	-17.0	2.4	-24.3	-15.2	19.4	26.7

[a] Conformation 1 with ether functionality of the maleimide's substituent pointing away from **Furan**; [b] conformation 2 with ether functionality of the maleimide's substituent pointing in the direction of **Furan**; [c] the glycol's hydroxyl group is forming a hydrogen bond with the furan's ring oxygen and the maleimide sidechain's ether group; [d] extra HB with other hydroxyl functionality of glycol.

Table S7. The Gibbs free energetics of the Diels-Alder reactions at 298.15 K, with and without addition of ethylene glycol. Hydrogen bond (HB) is formed with **Furan** (F) or **Maleimide** (M) (C=O: carbonyl; -OH: hydroxyl; (O): ring oxygen; -O-: ether). Values are relative to the separate reagents and given in kcal mol^{-1}. R1_gly: reactant complex between first reactant and glycol; RC: reactant complex of R1_gly with second reactant R2; TS: transition state; P_gly: product complex with glycol; P: product.

HB?	Endo/Exo	R1_gly + R2	RC	TS	P_gly	P (+ gly)	TS - RC	TS - P(_gly)
no	Endo[a]	-	5.4	26.8	-	0.7	21.4	26.1
	Endo[b]	-	5.9	26.8	-	1.2	20.8	25.6
	Exo[a]	-	4.7	27.7	-	-0.8	23.0	28.5
	Exo[b]	-	5.5	27.7	-	0.9	22.3	26.8
M[C=O] + F[-OH]	Endo[a]	-	4.4	24.6	-	-0.6	20.2	25.2
	Endo[b]	-	2.4	23.7	-	-0.2	21.3	23.9
	Exo[a]	-	2.3	24.5	-	-3.7	22.2	28.2
	Exo[b]	-	2.4	24.5	-	-2.3	22.1	26.8
F[(O)] + glycol	Endo[a]	5.6[d]	9.5[d]	31.8	4.6	1.1	22.3[d]	27.3
	Endo[b]	5.8[d]	8.1[d]	31.0	4.7	1.4	22.9[d]	26.4
	Exo[a]	5.0	10.5	35.1	3.8	-0.5	24.6	31.3
	Exo[b,c]	6.8	10.4	32.5	4.9	1.0	22.1	27.6
F[-O-] + glycol	Endo[a]	4.9	9.7	31.8	4.2	0.7	22.1	27.5
	Endo[b]	4.8	9.4	30.8	4.0	1.2	21.4	26.8
	Exo[a]	5.7	7.9	31.2	3.1	-0.8	23.3	28.1
	Exo[b]	5.7	8.1	30.6	4.1	0.5	22.5	26.5
M[C=O] + glycol	Endo[a]	5.3	7.7	27.5	3.0	0.7	19.8	24.4
	Endo[b]	3.2	8.7	27.6	4.0	1.2	18.8	23.6
	Exo[a]	4.7	7.6	28.6	1.1	-0.6	21.0	27.5
	Exo[b]	3.8	8.4	28.6	3.0	1.0	20.2	25.7
M[-O-] + glycol	Endo[a]	2.7	9.2	30.1	2.7	0.7	20.9	27.4
	Endo[b]	2.5	10.4	29.8	4.8	1.2	19.5	25.0
	Exo[a]	3.0	7.8	30.7	1.4	-0.6	22.9	29.3
	Exo[b]	2.9	9.4	30.2	3.4	0.5	20.8	26.8

[a] Conformation 1 with ether functionality of the maleimide's substituent pointing away from **Furan**; [b] conformation 2 with ether functionality of the maleimide's substituent pointing in the direction of **Furan**; [c] the glycol's hydroxyl group is forming a hydrogen bond with the furan's ring oxygen and the maleimide sidechain's ether group; [d] extra HB with other hydroxyl functionality of glycol.

Table S8. The energetics of the *endo* Diels-Alder reactions at 0 K (*E*) and 298.15 K (*H* and *G*), with and without addition of 2-methoxyethanol (cat). Hydrogen bond (HB) is formed with ring oxygen of **Furan**. Values are relative to the separate reagents and given in kcal mol^{-1}. R1_gly: reactant complex between first reactant and cat; RC: reactant complex of R1_gly with second reactant R2; TS: transition state; P_gly: product complex with cat; P: product.

HB?	Endo/Exo	Q	R1_gly + R2	RC	TS	P_gly	P (+ gly)	TS - RC	TS - P(_gly)
F[(O)] + cat	Endo[a]	*E*	-6.1	-15.0	7.8	-20.1	-13.6	22.8	27.9
		H	-6.5	-14.7	7.7	-20.7	-14.5	22.3	28.3
		G	7.6	10.3	31.9	4.9	1.1	21.6	26.9
	Endo[b]	*E*	-6.1	-15.9	6.4	-20.6	-13.8	22.3	27.0
		H	-6.5	-15.7	6.1	-21.2	-14.8	21.8	27.3
		G	7.6	10.5	31.8	4.7	1.4	21.3	27.0

[a] Conformation 1 with ether functionality of the maleimide's substituent pointing away from **Furan**; [b] conformation 2 with ether functionality of the maleimide's substituent pointing in the direction of **Furan**

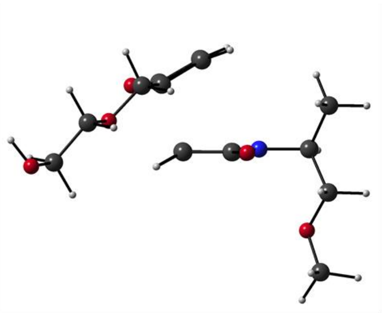

Endo TS (conf. A) w/o HB

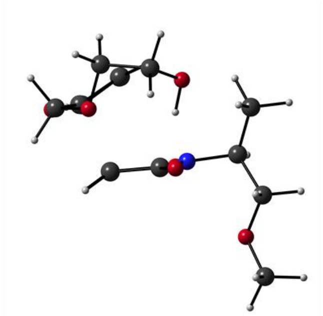

Endo TS (conf. A) with HB

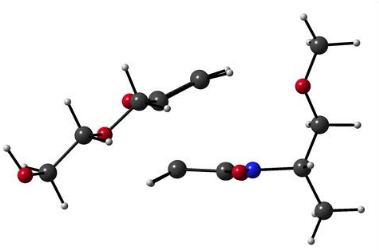

Endo TS (conf. B) w/o HB

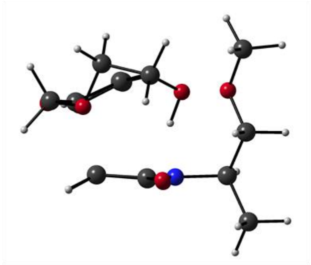

Endo TS (conf. B) with HB

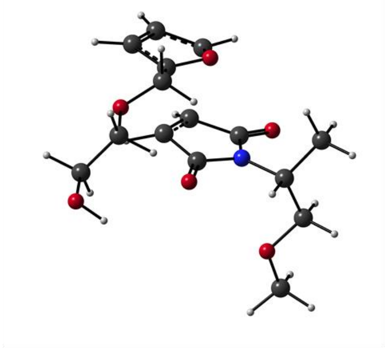

Exo TS (conf. A) w/o HB

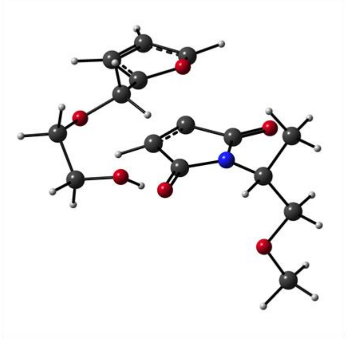

Exo TS (conf. A) with HB

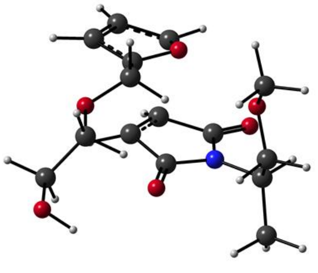

Exo TS (conf. B) w/o HB

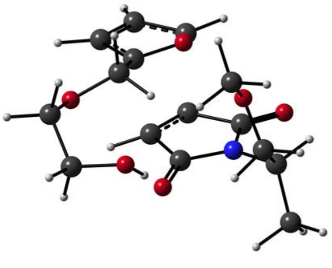

Exo TS (conf. B) with HB

Figure S4. Transition state structures for the uncoordinated and hydrogen-bond-assisted reactions, the latter originating from the furan's hydroxyl functionality and one of the maleimide's carbonyl groups.

Table S9. The energetics of the Diels-Alder reactions between **Maleimide** and ester-functionalized furan (with ester group instead of hydroxyl functionality) at 0 K (*E*) and 298.15 K (*G*). Values are relative to the separate reagents and given in kcal mol^{-1}. RC: reactant complex; TS: transition state; P: product.

Endo/Exo	Q	RC	TS	P	TS - RC	TS - P
Endo[a]	*E*	-8.2	12.3	-13.9	20.5	26.3
	G	5.6	27.4	3.7	21.8	23.8
Endo[b]	*E*	-8.0	11.1	-14.3	19.1	25.4
	G	6.5	27.2	3.8	20.7	23.4
Exo[a]	*E*	-8.5	12.6	-15.8	21.1	28.4
	G	5.5	28.8	2.2	23.3	26.6
Exo[b]	*E*	-9.4	11.5	-15.7	20.9	27.3
	G	6.6	29.2	3.3	22.6	25.9

[a] Conformation 1 with ether functionality of the maleimide's substituent pointing away from furan molecule; [b] conformation 2 with ether functionality of the maleimide's substituent pointing in the direction of furan molecule

Table S10. The computed kinetics of the Diels-Alder reactions between **Maleimide** and **Furan.** Δ*G* values (RC, TS, TS – RC) are given in kcal mol^{-1}. Rate constants at 20°C in s^{-1} (at the standard concentration of 1 mol L^{-1}) are computed via the Eyring-Polanyi equation from Transition State Theory. The rate constants of the individual reactions are then weighted via the Boltzmann population of the reactant complexes. RC: reactan complex; TS: transition state; P: product.

HB?	Endo/Exo	RC[c]	TS[c]	TS – RC	rate constant	Boltz. pop.	weighted rate constant
no	Endo[a]	5.4	26.8	21.4	7.20E-04	0.73	1.00E-03
	Endo[b]	5.9	26.8	20.8	1.76E-03	0.27	
no	Endo[a]	5.4	26.8	21.4	7.20E-04	0.00	9.15E-04
	Endo[b]	5.9	26.8	20.8	1.76E-03	0.00	
M[C=O] + F[-OH]	Endo[a]	4.4	24.6	20.2	5.32E-03	0.03	
	Endo[b]	2.4	23.7	21.3	7.82E-04	0.96	
no	Endo[a]	5.4	26.8	21.4	7.20E-04	0.00	3.48E-03
	Endo[b]	5.9	26.8	20.8	1.76E-03	0.00	
M[C=O] + F[-OH]	Endo[a]	4.4	24.6	20.2	5.32E-03	0.01	
	Endo[b]	2.4	23.7	21.3	7.82E-04	0.32	
F[(O)] + glycol	Endo[a]	3.9	26.2	22.3	1.45E-04	0.02	
	Endo[b]	2.3	25.2	22.9	5.16E-05	0.35	
F[-O-] + glycol	Endo[a]	4.8	26.9	22.1	2.04E-04	0.00	
	Endo[b]	4.6	26.0	21.4	6.78E-04	0.01	
M[C=O] + glycol	Endo[a]	2.4	22.2	19.8	1.06E-02	0.29	
	Endo[b]	5.5	24.4	18.9	4.95E-02	0.00	
M[-O-] + glycol	Endo[a]	6.5	27.4	20.9	1.60E-03	0.00	
	Endo[b]	7.9	27.3	19.4	2.10E-02	0.00	
no	Exo[a]	4.7	27.7	23.0	4.04E-05	0.80	6.38E-05
	Exo[b]	5.5	27.7	22.3	1.55E-04	0.20	
no	Exo[a]	4.7	27.7	23.0	4.04E-05	0.01	1.90E-04
	Exo[b]	5.5	27.7	22.3	1.55E-04	0.00	
M[C=O] + F[-OH]	Exo[a]	2.3	24.5	22.2	1.79E-04	0.53	
	Exo[b]	2.4	24.5	22.1	2.07E-04	0.45	
no	Exo[a]	4.7	27.7	23.0	4.04E-05	0.00	2.46E-04
	Exo[b]	5.5	27.7	22.3	1.55E-04	0.00	
M[C=O] + F[-OH]	Exo[a]	2.3	24.5	22.2	1.79E-04	0.22	
	Exo[b]	2.4	24.5	22.1	2.07E-04	0.19	
F[(O)] + glycol	Exo[a]	5.5	30.1	24.6	2.79E-06	0.00	
	Exo[b]	3.6	25.7	22.1	2.04E-04	0.02	
F[-O-] + glycol	Exo[a]	2.2	25.5	23.3	2.60E-05	0.27	
	Exo[b]	2.4	24.9	22.5	1.03E-04	0.19	
M[C=O] + glycol	Exo[a]	2.9	23.9	21.0	1.35E-03	0.08	
	Exo[b]	4.6	24.8	20.2	5.32E-03	0.00	
M[-O-] + glycol	Exo[a]	4.8	27.7	22.9	5.16E-05	0.00	
	Exo[b]	6.5	27.3	20.8	1.90E-03	0.00	

[a] Conformation 1 with ether functionality of the maleimide's substituent pointing away from furan molecule; [b] conformation 2 with ether functionality of the maleimide's substituent pointing in the direction of furan molecule; [c] for the reactions with glycol added, RC and TS are taken as the difference between the 3-component complex (R1_gly_R2) or transition state and the sum of reactant R1 in complex with glycol and reactant R2 (R1_gly+R2)

Table S11. The computed kinetics of the Diels-Alder reactions between **Maleimide** and ester-functionalized furan (with ester group instead of hydroxyl functionality). Δ*G* values (RC, TS, TS – RC) are given in kcal mol^{-1}. Rate constants at 20°C in s^{-1} (at the standard concentration of 1 mol L^{-1}) are computed via the Eyring-Polanyi equation from Transition State Theory. The rate constants of the individual reactions are then weighted via the Boltzmann population of the reactant complexes. RC: reactant complex; TS: transition state; P: product.

Endo/Exo	RC[c]	TS[c]	TS – RC	rate constant	Boltz. pop.	weighted rate constant
Endo[a]	5.6	27.4	21.8	3.41E-04	0.82	6.78E-04
Endo[b]	6.5	27.2	20.7	2.25E-03	0.18	
Exo[a]	5.5	28.8	23.3	2.60E-05	0.98	3.82E-05
Exo[b]	6.6	29.2	22.6	8.64E-05	0.15	

[a] Conformation 1 with ether functionality of the maleimide's substituent pointing away from furan molecule; [b] conformation 2 with ether functionality of the maleimide's substituent pointing in the direction of furan molecule

Table S12. Experimental[a] versus computed[b] Diels-Alder rate constants k_{DA} at 20°C. Exp. k_{DA} in kg mol^{-1} s^{-1}; comp. k_{DA} in s^{-1} (L mol^{-1}). Difference in associated $\Delta G^{\ddagger}$ given in kcal mol^{-1}.

Exp. system	Exp. k_{DA}	Comp. system	Comp. k_{DA}	Diff. in $\Delta G^{\ddagger}$
		No HB	1.06E-03	2.3
<Model Series>	2.29E-05	Poss. HB from **Furan**	1.10E-03	2.3
<4F400-2M400 + PPG425>	2.25E-05	Glycol additive	3.72E-03	3.0
4F400-ester-2M400	2.61E-05	Ester	7.16E-04	1.9
<FGE> (OH free)	2.18E-05			

[a] $k_{DA} = v_0$, normalized against the starting concentrations in mol kg^{-1} of the furan and maleimide functional groups; [b] with a standard concentration of 1 mol L^{-1} as reference and computed as the sum of *endo* and *exo* rate constants.

The computed rate constants are estimated to be approximately 42 times faster than the experimental ones for 4F400-2M400, which can be associated with a 2.2 kcal mol^{-1} deviation in $\Delta G^{\ddagger}$. The agreement is very reasonable, bearing in mind the difference in settings (computed gas phase vs. experimental liquid phase) and considering that the theoretical rate constants are an upper bound (only conformations leading to the lowest transition states were retained and the transmission coefficient was taken as 1).

Cartesian coordinates

Furan

6	0	-1.431128	-0.223420	0.000138
6	0	-1.767699	1.093007	0.000220
6	0	-3.205250	1.127041	-0.000000
6	0	-3.611207	-0.168913	0.000408
1	0	-1.069108	1.922142	0.000200
1	0	-3.847037	2.002242	-0.000176
1	0	-4.582479	-0.650544	0.000566
8	0	-2.537903	-1.003240	0.000283
6	0	-0.116710	-0.924972	0.000053
1	0	-0.035585	-1.579129	0.890700
1	0	-0.035783	-1.579302	-0.890485
8	0	0.887379	0.052782	-0.000148
6	0	2.178321	-0.506858	-0.000079
1	0	2.338164	-1.139803	-0.893405
1	0	2.338306	-1.139249	0.893616
6	0	3.174883	0.631636	-0.000498
1	0	3.000319	1.257945	0.892884
1	0	3.000554	1.257086	-0.894520
8	0	4.457241	0.042119	-0.000025
1	0	5.111644	0.750192	-0.001705

Maleimide

6	0	-2.406336	-1.028589	0.009646
6	0	-2.125698	-0.577156	1.231929
6	0	-0.927397	0.330590	1.141953
1	0	-3.192153	-1.704229	-0.319563
1	0	-2.617754	-0.781859	2.179783
6	0	-1.411158	-0.440963	-0.955097
8	0	-0.351729	0.882328	2.048745
8	0	-1.320093	-0.641509	-2.137982
7	0	-0.598061	0.412806	-0.205845
6	0	0.636252	0.990789	-0.727215
6	0	1.839017	0.307829	-0.084224
1	0	1.927121	0.611923	0.975835
1	0	2.757757	0.628372	-0.613870
8	0	1.654592	-1.080037	-0.187194
6	0	2.697846	-1.799750	0.417991
1	0	2.783101	-1.556236	1.492975
1	0	2.470309	-2.866923	0.307779
1	0	3.669660	-1.588163	-0.065169
6	0	0.672816	2.502039	-0.539273
1	0	-0.173427	2.972405	-1.056997
1	0	0.620268	2.754995	0.528567
1	0	1.604394	2.911816	-0.955242
1	0	0.622939	0.733259	-1.795992

Glycol

8	0	1.766679	-0.261008	0.000075
1	0	2.508788	0.354188	0.000806
6	0	0.573403	0.493538	-0.000413

1	0	0.483996	1.139013	0.892231
1	0	0.484171	1.138179	-0.893674
6	0	-0.573413	-0.493547	0.000071
1	0	-0.483737	-1.138501	0.893065
1	0	-0.484504	-1.138690	-0.892848
8	0	-1.766675	0.261024	0.000598
1	0	-2.508692	-0.354259	-0.002910

Furan_gly

6	0	-0.900758	-1.271129	-0.068030
6	0	-1.160187	-1.369919	1.261836
6	0	-2.584255	-1.532376	1.376853
6	0	-3.069754	-1.519956	0.109545
1	0	-0.418527	-1.323931	2.051441
1	0	-3.166608	-1.640799	2.286172
1	0	-4.062541	-1.612060	-0.316338
8	0	-2.052256	-1.360989	-0.785532
6	0	0.362106	-1.082813	-0.837179
1	0	0.609643	-2.000860	-1.405805
1	0	0.225201	-0.255086	-1.557614
8	0	1.375444	-0.788306	0.091612
6	0	2.615462	-0.555736	-0.535088
1	0	2.549753	0.297738	-1.235508
1	0	2.941561	-1.442299	-1.110499
6	0	3.630437	-0.255578	0.546221
1	0	3.662082	-1.107486	1.249046
1	0	3.301458	0.636764	1.109332
8	0	4.863406	-0.051267	-0.107801
1	0	5.530796	0.137592	0.561671
8	0	-1.551999	1.329995	-1.761936
1	0	-2.053985	0.508812	-1.829625
6	0	-1.775163	1.861410	-0.466593
1	0	-2.231091	1.119948	0.212250
1	0	-2.448775	2.734780	-0.515281
6	0	-0.447620	2.308774	0.113133
1	0	0.047266	2.974198	-0.619478
1	0	0.199114	1.425792	0.272437
8	0	-0.735689	2.975684	1.326377
1	0	0.101786	3.209906	1.741844

Furan_cocgly

6	0	-2.028952	0.483142	-0.256793
6	0	-2.128939	-0.513275	0.666594
6	0	-3.534886	-0.768819	0.812473
6	0	-4.163785	0.086890	-0.034702
1	0	-1.305971	-1.001519	1.182545
1	0	-4.008381	-1.494570	1.465600
1	0	-5.203948	0.270651	-0.279279
8	0	-3.258898	0.857564	-0.687910
6	0	-0.880350	1.203513	-0.878437
1	0	-0.503591	0.656785	-1.764845
1	0	-1.224838	2.196355	-1.218378
8	0	0.158574	1.332393	0.069953
6	0	1.310612	1.943300	-0.480143
1	0	1.079668	2.971249	-0.813065

1	0	1.675922	1.373669	-1.354678
6	0	2.380734	1.982710	0.591084
1	0	2.616961	0.950844	0.908185
1	0	1.983315	2.525507	1.468108
8	0	3.488443	2.636688	0.011944
1	0	4.207789	2.630694	0.653772
8	0	1.036921	-1.022931	1.282490
1	0	0.653821	-0.139845	1.148718
6	0	1.261608	-1.541299	-0.010746
1	0	0.333914	-1.560643	-0.614349
1	0	2.014572	-0.951194	-0.568161
6	0	1.776917	-2.955886	0.140501
1	0	2.686885	-2.934932	0.767711
1	0	1.016101	-3.557814	0.669949
8	0	2.034014	-3.442044	-1.160006
1	0	2.347592	-4.350238	-1.082596

Furan_IHB

6	0	0.910551	-0.503811	-0.407961
6	0	2.017505	-0.991416	0.215984
6	0	2.833680	0.142859	0.543014
6	0	2.158125	1.232990	0.094087
1	0	2.222726	-2.037592	0.420130
1	0	3.797724	0.144375	1.041217
1	0	2.362751	2.297712	0.101240
8	0	0.990317	0.856612	-0.484935
6	0	-0.343253	-1.128021	-0.934763
1	0	-0.824294	-0.412521	-1.628322
1	0	-0.094073	-2.030681	-1.506690
8	0	-1.232815	-1.553925	0.072588
6	0	-1.725226	-0.519889	0.907866
1	0	-0.900808	-0.021710	1.454424
1	0	-2.351663	-1.041797	1.643289
6	0	-2.549698	0.538713	0.174373
1	0	-3.368418	0.870939	0.829476
1	0	-3.015608	0.051094	-0.704893
8	0	-1.843074	1.707341	-0.179164
1	0	-0.953856	1.471410	-0.473388

Maleimide_gly

6	0	-0.555489	0.950746	-1.062290
6	0	0.200691	2.044687	-1.160551
6	0	1.378789	1.897386	-0.237538
1	0	-1.480318	0.671584	-1.565651
1	0	0.062492	2.930674	-1.775379
6	0	0.095295	0.023617	-0.070122
8	0	2.295639	2.655549	-0.069911
8	0	-0.267431	-1.094785	0.243912
7	0	1.204118	0.673749	0.427456
6	0	2.248460	0.056487	1.240472
6	0	1.739512	-0.307357	2.629138
1	0	0.917097	-1.032281	2.558002
1	0	2.551578	-0.750739	3.222895
1	0	1.378571	0.588100	3.151738
8	0	-2.807552	-1.137021	-0.962636

1	0	-1.937000	-1.418803	-0.633763
6	0	-3.494448	-0.587598	0.140473
1	0	-3.520354	-1.279279	1.000664
1	0	-3.032143	0.357181	0.489460
6	0	-4.912784	-0.298351	-0.300517
1	0	-5.382885	-1.245092	-0.622917
1	0	-4.882134	0.376278	-1.176436
8	0	-5.579615	0.285356	0.798743
1	0	-6.492109	0.454623	0.538282
1	0	3.028063	0.829016	1.303822
6	0	2.826718	-1.150101	0.508008
1	0	3.734674	-1.490472	1.043572
1	0	2.095944	-1.980805	0.505371
8	0	3.130796	-0.753766	-0.803485
6	0	3.642092	-1.812352	-1.573106
1	0	3.836029	-1.424308	-2.580263
1	0	4.586379	-2.201211	-1.149628
1	0	2.919572	-2.646360	-1.638743

Maleimide_cocgly

6	0	0.471709	-2.300575	-0.679618
6	0	0.548078	-2.193004	0.647478
6	0	-0.491184	-1.208500	1.109547
1	0	1.068385	-2.895414	-1.366612
1	0	1.228197	-2.673098	1.347380
6	0	-0.639953	-1.415458	-1.170134
8	0	-0.720388	-0.844521	2.239397
8	0	-1.058916	-1.282561	-2.289378
7	0	-1.151783	-0.775437	-0.034188
6	0	-2.261373	0.169599	-0.068374
6	0	-1.800377	1.541853	0.400804
1	0	-1.403945	1.480045	1.430715
1	0	-2.662805	2.234250	0.396205
8	0	-0.805343	2.009470	-0.485760
6	0	-0.325001	3.288643	-0.133974
1	0	0.096693	3.287830	0.886459
1	0	0.461630	3.549913	-0.852853
1	0	-1.131959	4.040227	-0.187812
6	0	-3.455415	-0.331812	0.738027
1	0	-3.769108	-1.318047	0.370993
1	0	-3.194776	-0.418139	1.801503
1	0	-4.301957	0.361005	0.632025
1	0	-2.523552	0.245546	-1.133995
8	0	1.389939	0.489384	-1.301208
1	0	0.539376	0.958889	-1.250986
6	0	2.054465	0.732914	-0.086536
1	0	2.215278	1.811907	0.096423
1	0	1.502827	0.337332	0.789989
6	0	3.403145	0.050332	-0.155088
1	0	3.973085	0.475937	-1.000803
1	0	3.244805	-1.024542	-0.363991
8	0	4.041292	0.264775	1.085972
1	0	4.923058	-0.121917	1.039692

Furan + Maleimide (no HB, conformation 1): Endo

- Reactant complex:

6	0	-0.557539	3.005498	-0.432466
6	0	0.526336	3.457765	0.257254
6	0	1.248937	1.788697	-0.946640
6	0	-0.080509	1.910226	-1.223628
6	0	-0.249547	0.951131	2.193509
6	0	0.710459	0.211170	1.636104
1	0	0.670229	4.257881	0.975437
1	0	-1.569721	3.392657	-0.371893
1	0	-0.648743	1.277428	-1.898328
8	0	1.628336	2.736741	-0.052784
6	0	-1.572922	0.528497	1.624507
6	0	0.068188	-0.743149	0.666778
1	0	1.787857	0.250897	1.765649
1	0	-0.172202	1.745846	2.931141
7	0	-1.293862	-0.467305	0.683325
8	0	0.600098	-1.598174	-0.007824
8	0	-2.670997	0.944511	1.894348
6	0	-2.325098	-1.314971	0.098921
1	0	-3.264959	-0.916773	0.507546
6	0	2.287837	0.845970	-1.442380
1	0	1.784517	0.118753	-2.104063
1	0	3.052751	1.381840	-2.036694
8	0	2.895796	0.191245	-0.353257
6	0	3.574126	-0.983083	-0.734671
1	0	2.915580	-1.635026	-1.335159
1	0	4.470662	-0.735239	-1.341092
6	0	3.981583	-1.713411	0.532560
1	0	4.520604	-1.013209	1.197477
1	0	3.069341	-2.044455	1.047841
8	0	4.733009	-2.870995	0.244376
1	0	5.575938	-2.586529	-0.131097
6	0	-2.158379	-2.752332	0.584916
1	0	-3.058273	-3.331883	0.298415
1	0	-1.278357	-3.217237	0.103352
6	0	-2.329230	-1.232964	-1.422594
1	0	-3.078021	-1.922144	-1.839604
1	0	-2.581417	-0.215934	-1.751967
1	0	-1.340813	-1.507004	-1.818717
8	0	-1.999814	-2.722598	1.978996
6	0	-1.734764	-3.996130	2.508796
1	0	-0.807446	-4.421503	2.083268
1	0	-1.614703	-3.884096	3.593159
1	0	-2.564920	-4.699235	2.310308

- Transition state:

6	0	-0.246205	-2.849795	0.318301
6	0	0.470640	-2.712116	-0.897783
6	0	1.404693	-1.351997	0.476604
6	0	0.349561	-1.976799	1.199096
6	0	-0.362580	-0.864248	-1.559028
6	0	0.276420	0.005239	-0.664933

1	0	0.454643	-3.359144	-1.770477
1	0	-1.167471	-3.411814	0.445368
1	0	0.006896	-1.682276	2.187897
8	0	1.657887	-2.099221	-0.637135
6	0	-1.806187	-0.923292	-1.175810
6	0	-0.745902	0.489847	0.307692
1	0	1.153487	0.611244	-0.877632
1	0	-0.082367	-1.022029	-2.597663
7	0	-1.933629	-0.164560	-0.004074
8	0	-0.594938	1.262837	1.231574
8	0	-2.709172	-1.522913	-1.707295
6	0	-3.203651	0.029785	0.681228
1	0	-3.922062	-0.558160	0.091974
6	0	2.538631	-0.570977	1.058561
1	0	2.116004	0.113338	1.818181
1	0	3.243275	-1.260635	1.563369
8	0	3.199537	0.136013	0.042659
6	0	4.306383	0.860597	0.525990
1	0	4.003408	1.576866	1.311904
1	0	5.052569	0.170122	0.970776
6	0	4.924782	1.607945	-0.642897
1	0	5.158711	0.888105	-1.447220
1	0	4.193306	2.324182	-1.041581
8	0	6.047535	2.355463	-0.235607
1	0	6.749995	1.733080	-0.008575
6	0	-3.621957	1.494042	0.612921
1	0	-4.641178	1.593211	1.037523
1	0	-2.932528	2.113206	1.215985
6	0	-3.143452	-0.475813	2.118459
1	0	-4.114726	-0.336834	2.614807
1	0	-2.899663	-1.547337	2.135977
1	0	-2.376711	0.074772	2.681897
8	0	-3.598272	1.890482	-0.732938
6	0	-3.898582	3.252518	-0.887261
1	0	-3.174076	3.888611	-0.345999
1	0	-3.848716	3.482961	-1.958464
1	0	-4.913578	3.489543	-0.516449

- Endo product:

6	0	-0.258440	-2.934977	0.391198
6	0	0.253019	-2.581480	-0.998355
6	0	1.267964	-1.244729	0.322525
6	0	0.374594	-2.098719	1.218424
6	0	-0.403419	-1.208354	-1.400301
6	0	0.327844	-0.252619	-0.455149
1	0	0.227361	-3.354514	-1.769810
1	0	-1.055586	-3.641364	0.615367
1	0	0.219733	-1.949363	2.286171
8	0	1.583674	-2.155652	-0.729724
6	0	-1.871154	-1.066879	-1.045667
6	0	-0.752341	0.371352	0.399557
1	0	0.944142	0.510560	-0.941538
1	0	-0.246668	-1.007018	-2.465629
7	0	-1.977087	-0.133340	-0.014087
8	0	-0.593995	1.142285	1.320184
8	0	-2.801863	-1.666836	-1.523543

6	0	-3.261354	0.217515	0.587351
1	0	-4.003008	-0.273867	-0.057717
6	0	2.486536	-0.625473	0.950405
1	0	2.160129	-0.018941	1.816883
1	0	3.153166	-1.431463	1.310543
8	0	3.136490	0.174041	-0.002304
6	0	4.293266	0.791109	0.506646
1	0	4.057803	1.423107	1.383479
1	0	5.026925	0.025399	0.833904
6	0	4.894248	1.644889	-0.596345
1	0	5.068124	1.013481	-1.485722
1	0	4.175334	2.425797	-0.879877
8	0	6.061128	2.303373	-0.158853
1	0	6.743965	1.633768	-0.025689
6	0	-3.484596	1.722725	0.515110
1	0	-4.519506	1.939160	0.847319
1	0	-2.785862	2.246711	1.191916
6	0	-3.362355	-0.311446	2.013722
1	0	-4.346182	-0.068936	2.440401
1	0	-3.242722	-1.403659	2.022190
1	0	-2.582091	0.138045	2.643620
8	0	-3.288192	2.128689	-0.814323
6	0	-3.427697	3.517594	-0.966302
1	0	-2.696847	4.065820	-0.343902
1	0	-3.249585	3.754397	-2.022325
1	0	-4.443628	3.856001	-0.689385

Furan + Maleimide (no HB, conformation 2): Endo

- Reactant complex:

6	0	-1.254782	2.547622	-0.021291
6	0	-0.302644	3.169723	0.728325
6	0	0.782657	1.923850	-0.695159
6	0	-0.540879	1.732681	-0.959194
6	0	-0.462869	0.283522	2.193596
6	0	0.660356	-0.124680	1.601474
1	0	-0.345384	3.875343	1.551286
1	0	-2.330299	2.638366	0.093434
1	0	-0.974585	1.062483	-1.695150
8	0	0.936905	2.806718	0.326637
6	0	-1.641179	-0.321960	1.480595
6	0	0.281897	-1.030169	0.464082
1	0	1.695211	0.131572	1.807348
1	0	-0.598452	0.947633	3.043407
7	0	-1.111689	-1.106262	0.457487
8	0	1.000680	-1.615147	-0.313113
8	0	-2.810392	-0.172435	1.745792
6	0	-1.819951	-1.926951	-0.522007
1	0	-1.196279	-1.885548	-1.428151
6	0	2.014064	1.345867	-1.296023
1	0	1.697453	0.619184	-2.064942
1	0	2.619817	2.130501	-1.788763
8	0	2.776384	0.709971	-0.294153
6	0	3.732600	-0.177805	-0.823751
1	0	3.260974	-0.881704	-1.531951
1	0	4.526869	0.381257	-1.362196

6	0	4.331828	-0.955418	0.334403
1	0	4.673982	-0.244129	1.109072
1	0	3.545377	-1.586801	0.770639
8	0	5.358603	-1.820313	-0.095800
1	0	6.087648	-1.272624	-0.413598
6	0	-3.191231	-1.355875	-0.849913
1	0	-3.625771	-1.958387	-1.671010
1	0	-3.853451	-1.431799	0.027384
6	0	-1.920271	-3.372789	-0.045958
1	0	-2.414132	-3.996079	-0.805388
1	0	-0.916190	-3.778931	0.134096
1	0	-2.500277	-3.429911	0.887287
8	0	-3.047766	-0.013856	-1.243159
6	0	-4.261721	0.693543	-1.162157
1	0	-4.612202	0.741413	-0.116059
1	0	-4.075343	1.709098	-1.534998
1	0	-5.045715	0.226415	-1.785921

- Transition state:

6	0	-0.929225	1.953176	0.599312
6	0	-0.068406	1.742745	1.706547
6	0	1.055879	1.139593	-0.019985
6	0	-0.212848	1.564925	-0.507300
6	0	-0.263699	-0.378757	1.828537
6	0	0.509858	-0.737359	0.713940
1	0	-0.171349	2.099477	2.727791
1	0	-1.988636	2.187214	0.654239
1	0	-0.597444	1.406737	-1.511455
8	0	1.207739	1.616761	1.251525
6	0	-1.693157	-0.653348	1.492433
6	0	-0.417356	-1.230378	-0.344536
1	0	1.539977	-1.084018	0.736940
1	0	0.057650	-0.434152	2.865768
7	0	-1.712736	-1.088989	0.165406
8	0	-0.152713	-1.642055	-1.451073
8	0	-2.676369	-0.513550	2.187629
6	0	-2.912650	-1.369171	-0.612998
1	0	-2.535605	-1.625475	-1.613832
6	0	2.293465	0.929942	-0.831355
1	0	2.003315	0.370734	-1.740653
1	0	2.703708	1.911008	-1.141353
8	0	3.244883	0.225447	-0.077213
6	0	4.447159	0.026480	-0.782798
1	0	4.271593	-0.523881	-1.725578
1	0	4.904790	1.002927	-1.044902
6	0	5.389672	-0.763717	0.108459
1	0	5.501511	-0.235542	1.072029
1	0	4.948379	-1.747566	0.319031
8	0	6.622868	-0.998658	-0.531486
1	0	7.081798	-0.152485	-0.607189
6	0	-3.783592	-0.127171	-0.740666
1	0	-4.696423	-0.392793	-1.308542
1	0	-4.091358	0.225255	0.261721
6	0	-3.693940	-2.541215	-0.025035
1	0	-4.555440	-2.783992	-0.663311
1	0	-3.049264	-3.428005	0.037011

1	0	-4.051904	-2.294819	0.983793
8	0	-3.062564	0.875353	-1.420435
6	0	-3.831375	2.035986	-1.608063
1	0	-4.177066	2.452896	-0.643103
1	0	-3.198401	2.777212	-2.112624
1	0	-4.720140	1.835415	-2.234138

- Endo product:

6	0	0.863904	-2.151778	0.234838
6	0	0.121457	-1.813295	1.517949
6	0	-1.054091	-0.957305	-0.045783
6	0	0.133209	-1.611934	-0.743428
6	0	0.350349	-0.274727	1.777177
6	0	-0.504000	0.335364	0.664056
1	0	0.261627	-2.458263	2.388505
1	0	1.853441	-2.600249	0.170803
1	0	0.393286	-1.495862	-1.794241
8	0	-1.234634	-1.799811	1.091188
6	0	1.761843	0.207934	1.499066
6	0	0.455508	1.132675	-0.189759
1	0	-1.351536	0.943530	0.996626
1	0	0.041890	-0.004750	2.793218
7	0	1.727990	1.030636	0.378367
8	0	0.202345	1.737119	-1.202803
8	0	2.763924	-0.081550	2.112333
6	0	2.918270	1.556082	-0.289235
1	0	2.522666	2.080469	-1.170438
6	0	-2.313786	-0.769253	-0.845426
1	0	-2.064197	-0.191552	-1.755859
1	0	-2.697326	-1.761829	-1.147609
8	0	-3.257042	-0.085231	-0.062424
6	0	-4.470532	0.127643	-0.740338
1	0	-4.314464	0.706026	-1.670277
1	0	-4.929639	-0.842365	-1.023464
6	0	-5.400960	0.887663	0.188779
1	0	-5.498648	0.328319	1.136217
1	0	-4.956302	1.864189	0.424810
8	0	-6.644413	1.142899	-0.423935
1	0	-7.099183	0.297531	-0.528576
6	0	3.795692	0.410171	-0.776672
1	0	4.650506	0.835026	-1.338813
1	0	4.195416	-0.156838	0.084402
6	0	3.685634	2.520087	0.608402
1	0	4.538532	2.944517	0.060085
1	0	3.033922	3.346144	0.922640
1	0	4.057632	2.002252	1.502415
8	0	3.021202	-0.423391	-1.603541
6	0	3.758457	-1.518325	-2.081982
1	0	4.159649	-2.129399	-1.251715
1	0	3.083003	-2.135454	-2.688249
1	0	4.607013	-1.192183	-2.711447

Furan + Maleimide (no HB, conformation 1): Exo

- Reactant complex:

6	0	-2.347480	-2.747274	0.444514
6	0	-1.956420	-1.820337	-0.475296
6	0	-0.234996	-3.134618	-0.159299
6	0	-1.220791	-3.605614	0.654456
1	0	-3.321568	-2.795957	0.920849
1	0	-1.151222	-4.461658	1.318010
6	0	-0.364887	0.039318	1.477696
1	0	-1.336420	0.326846	1.868685
6	0	0.508297	-0.867244	1.921205
1	0	0.454097	-1.514504	2.792826
1	0	0.787519	-3.442694	-0.352598
8	0	-0.670664	-2.055433	-0.851691
6	0	1.673900	-0.914802	0.972957
6	0	0.188963	0.646691	0.216646
7	0	1.408151	0.020661	-0.019419
8	0	-0.301791	1.501788	-0.483455
8	0	2.652041	-1.624062	1.030289
6	0	-2.622880	-0.627853	-1.072364
1	0	-3.543499	-0.922619	-1.609699
1	0	-1.928908	-0.170895	-1.800123
8	0	-2.935359	0.287572	-0.045673
6	0	-3.359749	1.532615	-0.556531
1	0	-4.366386	1.457341	-1.009526
1	0	-2.649585	1.881730	-1.329727
6	0	2.272442	0.327181	-1.151595
1	0	1.753502	1.143014	-1.675033
6	0	-3.375906	2.524405	0.592138
1	0	-2.361137	2.570117	1.025469
1	0	-4.068674	2.174486	1.370815
8	0	-3.836671	3.788864	0.170885
1	0	-3.160109	4.161668	-0.408970
6	0	3.612161	0.858563	-0.660697
1	0	4.151691	0.068460	-0.105295
1	0	4.226362	1.144521	-1.537832
6	0	2.431076	-0.880515	-2.069143
1	0	1.444387	-1.235856	-2.395599
1	0	3.019972	-0.610441	-2.957299
1	0	2.944698	-1.695980	-1.540234
8	0	3.364623	1.967714	0.162888
6	0	4.545256	2.490517	0.713760
1	0	5.239340	2.841956	-0.072542
1	0	4.266321	3.340698	1.348084
1	0	5.071201	1.737389	1.329037

- Transition state:

6	0	-2.090070	2.612280	-0.604767
6	0	-1.571128	1.666980	0.319517
6	0	0.013775	3.076499	-0.034626
6	0	-1.085956	3.527775	-0.813887
1	0	-3.047693	2.517629	-1.108424
1	0	-1.054150	4.339877	-1.534873
6	0	-0.364284	0.610366	-1.167513
1	0	-1.141653	0.367428	-1.885242
6	0	0.659985	1.559379	-1.313444
1	0	0.871591	2.143240	-2.205468
1	0	0.920609	3.606651	0.248148

8	0	-0.453092	2.183386	0.887486
6	0	1.816039	1.103190	-0.473157
6	0	0.147191	-0.464405	-0.269897
7	0	1.407338	-0.056417	0.174501
8	0	-0.396596	-1.492438	0.073141
8	0	2.894220	1.638745	-0.334207
6	0	-2.320661	0.619171	1.081434
1	0	-3.002339	1.091768	1.814711
1	0	-1.602922	-0.010686	1.634322
8	0	-3.030484	-0.125051	0.129894
6	0	-3.445814	-1.390298	0.601871
1	0	-4.320380	-1.302418	1.272560
1	0	-2.616933	-1.869746	1.153764
6	0	2.202345	-0.804477	1.139266
1	0	1.575300	-1.671277	1.392930
6	0	-3.792707	-2.235837	-0.610066
1	0	-2.904072	-2.279975	-1.263274
1	0	-4.608123	-1.758412	-1.171845
8	0	-4.250910	-3.513508	-0.227320
1	0	-3.492871	-3.995676	0.127319
6	0	3.475531	-1.324749	0.485087
1	0	4.119653	-0.477094	0.186247
1	0	4.030168	-1.939256	1.222420
6	0	2.494657	0.033305	2.379596
1	0	1.555731	0.397563	2.818695
1	0	3.023404	-0.568661	3.132239
1	0	3.116168	0.900377	2.116424
8	0	3.108675	-2.091722	-0.631738
6	0	4.225675	-2.558973	-1.340910
1	0	4.860356	-3.213565	-0.714264
1	0	3.856304	-3.136291	-2.197318
1	0	4.848515	-1.723099	-1.709662

- Exo product:

6	0	-1.931244	2.646355	-0.807097
6	0	-1.406222	1.554598	0.117955
6	0	0.206291	2.934974	-0.099841
6	0	-0.926494	3.515731	-0.934947
1	0	-2.898868	2.621913	-1.304428
1	0	-0.861319	4.398546	-1.567866
6	0	-0.411309	0.715915	-0.769276
1	0	-0.878352	0.368528	-1.695221
6	0	0.756824	1.706991	-0.896925
1	0	1.077556	1.959331	-1.913519
1	0	0.972665	3.606860	0.296710
8	0	-0.495688	2.268494	0.947535
6	0	1.900985	1.060181	-0.134863
6	0	0.161252	-0.462770	-0.001534
7	0	1.472148	-0.165191	0.351366
8	0	-0.412119	-1.484218	0.298540
8	0	2.998784	1.532757	0.047202
6	0	-2.403499	0.748809	0.911993
1	0	-3.087220	1.421666	1.460347
1	0	-1.863349	0.124794	1.644442
8	0	-3.090009	-0.044095	-0.023277
6	0	-3.615559	-1.234974	0.520384

1	0	-4.461233	-1.028872	1.202389
1	0	-2.824587	-1.764576	1.083566
6	0	2.315051	-1.094331	1.101535
1	0	1.682136	-1.982658	1.234251
6	0	-4.078174	-2.101292	-0.636742
1	0	-3.222650	-2.258345	-1.317550
1	0	-4.864689	-1.577857	-1.198286
8	0	-4.637987	-3.312282	-0.180180
1	0	-3.923088	-3.830636	0.211161
6	0	3.524498	-1.491238	0.264159
1	0	4.206002	-0.629245	0.144489
1	0	4.071011	-2.295660	0.795228
6	0	2.716291	-0.513808	2.452565
1	0	1.822617	-0.262536	3.039226
1	0	3.306895	-1.249289	3.017167
1	0	3.316331	0.395890	2.315871
8	0	3.061571	-1.936944	-0.984462
6	0	4.115935	-2.295807	-1.839674
1	0	4.712806	-3.127269	-1.420718
1	0	3.675391	-2.618442	-2.790899
1	0	4.792758	-1.441518	-2.024924

Furan + Maleimide (no HB, conformation 2): Exo

- Reactant complex:

6	0	-0.605390	3.182388	-0.059170
6	0	-0.400192	1.988715	0.564604
6	0	1.568589	2.680994	-0.097116
6	0	0.682799	3.634893	-0.494795
1	0	-1.567757	3.662931	-0.203179
1	0	0.920278	4.547653	-1.032069
6	0	-0.459973	0.042020	-1.989609
1	0	-1.460320	0.318034	-2.310315
6	0	0.718646	0.588109	-2.293738
1	0	0.947505	1.431161	-2.940537
1	0	2.641615	2.559543	-0.202305
8	0	0.923247	1.678329	0.550990
6	0	1.791083	-0.133104	-1.526223
6	0	-0.220967	-1.102995	-1.042135
7	0	1.153713	-1.150925	-0.821484
8	0	-1.029757	-1.863790	-0.566263
8	0	2.975080	0.110127	-1.496733
6	0	-1.333365	0.991436	1.163167
1	0	-1.801792	1.396441	2.081138
1	0	-0.758424	0.088117	1.433436
8	0	-2.334965	0.692814	0.211906
6	0	-3.244474	-0.269330	0.699962
1	0	-3.869567	0.151578	1.510506
1	0	-2.692984	-1.141271	1.100095
6	0	1.803821	-2.068671	0.109193
1	0	0.998702	-2.738948	0.443864
6	0	-4.120528	-0.715632	-0.455940
1	0	-3.469337	-1.114903	-1.252653
1	0	-4.670353	0.148076	-0.856382
8	0	-5.084000	-1.654987	-0.032032
1	0	-4.609611	-2.464259	0.198500

6	0	2.910915	-2.866216	-0.572686
1	0	3.733472	-2.206808	-0.879631
1	0	3.301031	-3.628811	0.115532
6	0	2.306856	-1.309999	1.329511
1	0	2.942316	-1.980958	1.940044
1	0	2.923921	-0.449877	1.007953
8	0	1.195262	-0.878983	2.073889
6	0	1.566184	-0.038641	3.138739
1	0	0.646453	0.260550	3.658550
1	0	2.225046	-0.565249	3.853469
1	0	2.083245	0.865058	2.771582
1	0	2.517607	-3.373453	-1.463848

- Transition state:

6	0	-0.795553	2.933738	0.175840
6	0	-0.526255	1.606384	0.603629
6	0	1.387229	2.485828	0.160830
6	0	0.427926	3.507802	-0.077549
1	0	-1.790791	3.327370	-0.009126
1	0	0.637844	4.475997	-0.523522
6	0	-0.138996	0.890759	-1.456084
1	0	-1.037901	1.237889	-1.956313
6	0	1.158460	1.413712	-1.584163
1	0	1.477463	2.164976	-2.302499
1	0	2.464841	2.568699	0.285545
8	0	0.789302	1.505528	0.906186
6	0	2.116476	0.310833	-1.228899
6	0	-0.016019	-0.549367	-1.116080
7	0	1.351030	-0.816537	-0.951953
8	0	-0.887994	-1.381196	-0.985031
8	0	3.323301	0.377609	-1.139511
6	0	-1.471378	0.627547	1.226234
1	0	-1.728962	0.954405	2.253318
1	0	-0.981937	-0.358836	1.285041
8	0	-2.605189	0.607822	0.399943
6	0	-3.402539	-0.546663	0.564894
1	0	-4.045333	-0.466991	1.460992
1	0	-2.753543	-1.435084	0.667751
6	0	1.845952	-2.027681	-0.306044
1	0	0.971563	-2.693259	-0.265620
6	0	-4.253271	-0.693849	-0.683596
1	0	-3.575761	-0.750161	-1.553004
1	0	-4.892882	0.192477	-0.801646
8	0	-5.111901	-1.809281	-0.589024
1	0	-4.558547	-2.599744	-0.631946
6	0	2.972923	-2.682865	-1.095920
1	0	3.859471	-2.036683	-1.121417
1	0	3.238925	-3.645380	-0.636702
6	0	2.249292	-1.702487	1.125469
1	0	2.704951	-2.594263	1.597924
1	0	3.002046	-0.891068	1.122503
8	0	1.094063	-1.307661	1.826148
6	0	1.396097	-0.745317	3.078092
1	0	0.445735	-0.470888	3.554764
1	0	1.924886	-1.466828	3.727567
1	0	2.017658	0.160933	2.968557

1	0	2.648349	-2.870609	-2.128113

- Exo product:

6	0	-0.899508	3.006111	0.023969
6	0	-0.579497	1.547686	0.327926
6	0	1.311667	2.488005	0.014677
6	0	0.283568	3.593560	-0.172715
1	0	-1.904344	3.405937	-0.097549
1	0	0.496601	4.612000	-0.491256
6	0	-0.172449	0.945612	-1.078364
1	0	-0.915933	1.176881	-1.845651
6	0	1.229192	1.554233	-1.235650
1	0	1.446169	2.057120	-2.184515
1	0	2.329105	2.750704	0.317547
8	0	0.682239	1.645149	0.979114
6	0	2.172746	0.381981	-1.002560
6	0	0.052853	-0.548895	-0.985167
7	0	1.425916	-0.785763	-0.926714
8	0	-0.789896	-1.413190	-0.941584
8	0	3.370969	0.452464	-0.857811
6	0	-1.567363	0.724973	1.115838
1	0	-1.820247	1.238483	2.061496
1	0	-1.104510	-0.249879	1.349975
8	0	-2.691484	0.575332	0.285288
6	0	-3.435234	-0.598449	0.531503
1	0	-4.050261	-0.502772	1.445332
1	0	-2.747636	-1.455704	0.653087
6	0	1.946607	-2.056891	-0.419191
1	0	1.089647	-2.742431	-0.475687
6	0	-4.323052	-0.837933	-0.675964
1	0	-3.677644	-0.900119	-1.569790
1	0	-5.006548	0.012793	-0.807577
8	0	-5.125920	-1.984936	-0.504164
1	0	-4.536400	-2.750028	-0.506669
6	0	3.109430	-2.590114	-1.244169
1	0	3.971638	-1.913880	-1.190757
1	0	3.403738	-3.579968	-0.867813
6	0	2.300983	-1.858392	1.051097
1	0	2.656343	-2.815014	1.479910
1	0	3.116432	-1.116148	1.133459
8	0	1.146572	-1.403677	1.714847
6	0	1.439720	-0.780074	2.942183
1	0	0.487075	-0.455272	3.379737
1	0	1.936838	-1.477851	3.640511
1	0	2.079990	0.106099	2.792711
1	0	2.810710	-2.698224	-2.295503

Furan + Maleimide (HB: maleimide C=O + furan -OH, conformation 1): Endo

- Reactant complex:

6	0	-1.872700	1.814926	1.283625
6	0	-2.690276	2.391588	0.361833
6	0	-2.528921	0.290649	-0.212271
6	0	-1.764268	0.433931	0.907286
6	0	0.129506	2.523314	-1.234770

6	0	-0.025514	1.333376	-1.814941
1	0	-3.070207	3.398101	0.224495
1	0	-1.393933	2.316000	2.119609
1	0	-1.180378	-0.351594	1.378732
8	0	-3.099762	1.473635	-0.551528
6	0	0.985636	2.336383	-0.013988
6	0	0.699044	0.299959	-0.996917
1	0	-0.575736	1.061192	-2.712623
1	0	-0.256295	3.497861	-1.522589
7	0	1.290947	0.973697	0.058373
8	0	0.727065	-0.892473	-1.217216
8	0	1.362486	3.169929	0.770622
6	0	2.214060	0.463994	1.077788
6	0	-2.855588	-0.884488	-1.088358
1	0	-2.687031	-0.611757	-2.140732
6	0	1.497466	0.133601	2.382315
1	0	2.237051	-0.088845	3.165477
6	0	2.997695	-0.732156	0.557501
1	0	2.334953	-1.608847	0.459456
1	0	3.778771	-0.978425	1.303065
1	0	2.914442	1.295633	1.245523
1	0	0.848791	-0.746279	2.258786
8	0	-2.035067	-1.985411	-0.812740
6	0	-2.389893	-2.729046	0.340115
1	0	-2.750795	-2.060810	1.140353
1	0	-3.186639	-3.457929	0.101665
6	0	-1.119925	-3.432924	0.783511
1	0	-0.823161	-4.162056	0.008351
1	0	-1.303492	-3.982944	1.717475
1	0	-3.927467	-1.142187	-0.986867
8	0	-0.089238	-2.502288	1.025174
1	0	0.077265	-2.043782	0.179436
8	0	3.570864	-0.388866	-0.677327
6	0	3.951435	-1.523841	-1.414764
1	0	4.404814	-1.171516	-2.349619
1	0	4.692482	-2.135105	-0.867089
1	0	3.071632	-2.149376	-1.648306
1	0	0.900978	0.994977	2.713118

- Transition state:

6	0	-1.038816	1.881259	1.493714
6	0	-1.141144	2.845377	0.455766
6	0	-2.108075	1.076680	-0.291844
6	0	-1.637638	0.740784	1.009044
6	0	0.372653	2.158902	-0.811181
6	0	-0.210981	0.976651	-1.294131
1	0	-0.997244	3.920815	0.518975
1	0	-0.448815	1.997207	2.398997
1	0	-1.619930	-0.268526	1.419115
8	0	-2.103082	2.430868	-0.422561
6	0	1.471589	1.766821	0.126236
6	0	0.503311	-0.165652	-0.660719
1	0	-0.661966	0.840095	-2.274075
1	0	0.462165	3.089358	-1.367195
7	0	1.410887	0.370732	0.236866
8	0	0.337938	-1.359473	-0.843722

8	0	2.243255	2.472340	0.727411
6	0	2.291089	-0.403433	1.104382
6	0	-3.096734	0.328726	-1.146617
1	0	-2.981218	0.637060	-2.195259
6	0	1.497012	-1.162447	2.162670
1	0	2.177238	-1.741555	2.804065
6	0	3.172764	-1.331575	0.276614
1	0	2.563806	-2.139886	-0.166526
1	0	3.927404	-1.790242	0.946430
1	0	2.937577	0.352939	1.572225
1	0	0.778045	-1.854140	1.699891
8	0	-2.866491	-1.050327	-1.060673
6	0	-3.605557	-1.712300	-0.045719
1	0	-3.742743	-1.049979	0.827450
1	0	-4.602338	-2.002995	-0.425836
6	0	-2.800252	-2.929038	0.366022
1	0	-2.700150	-3.605627	-0.501593
1	0	-3.338820	-3.470920	1.157405
1	0	-4.123713	0.594063	-0.834701
8	0	-1.543600	-2.555581	0.876008
1	0	-1.019165	-2.207903	0.129943
8	0	3.789702	-0.566833	-0.725758
6	0	4.561926	-1.357086	-1.591021
1	0	4.999725	-0.691421	-2.344937
1	0	5.378164	-1.872771	-1.051033
1	0	3.943641	-2.121003	-2.097459
1	0	0.943470	-0.459651	2.801112

- Endo product:

6	0	-1.166261	1.978293	1.422332
6	0	-0.963823	2.831393	0.178962
6	0	-1.844256	1.014142	-0.525280
6	0	-1.702906	0.834936	0.984208
6	0	0.243013	2.204689	-0.614151
6	0	-0.382100	0.894566	-1.111949
1	0	-0.934342	3.917867	0.289564
1	0	-0.807558	2.220656	2.421113
1	0	-1.856254	-0.105003	1.515613
8	0	-2.043056	2.420334	-0.650466
6	0	1.411047	1.783834	0.254671
6	0	0.473654	-0.198794	-0.503934
1	0	-0.419872	0.787107	-2.202636
1	0	0.576119	2.882920	-1.407285
7	0	1.462493	0.387988	0.258002
8	0	0.326199	-1.399119	-0.622990
8	0	2.153950	2.496299	0.880992
6	0	2.424295	-0.356152	1.071128
6	0	-2.931707	0.255085	-1.257812
1	0	-2.856431	0.467236	-2.335117
6	0	1.724954	-1.045908	2.238168
1	0	2.456836	-1.602871	2.840811
6	0	3.211863	-1.334802	0.208616
1	0	2.569448	-2.176978	-0.103283
1	0	4.045231	-1.738789	0.817135
1	0	3.115039	0.415528	1.438151
1	0	0.958420	-1.747292	1.878180

8	0	-2.779437	-1.129104	-1.062960
6	0	-3.629175	-1.694692	-0.079259
1	0	-3.835286	-0.962104	0.721024
1	0	-4.592208	-1.998842	-0.529574
6	0	-2.897481	-2.886213	0.506975
1	0	-2.746188	-3.643923	-0.283123
1	0	-3.511346	-3.340631	1.298482
1	0	-3.912364	0.618220	-0.907347
8	0	-1.675425	-2.489295	1.081104
1	0	-1.110860	-2.185049	0.347650
8	0	3.697584	-0.638590	-0.909732
6	0	4.393899	-1.481480	-1.791119
1	0	4.731011	-0.869904	-2.636925
1	0	5.276140	-1.937459	-1.304399
1	0	3.745026	-2.293695	-2.166856
1	0	1.243369	-0.300483	2.885890

Furan + Maleimide (HB: maleimide C=O + furan -OH, conformation 2): Endo

- Reactant complex:

6	0	0.390053	2.344594	0.482742
6	0	-0.244072	2.967971	-0.548443
6	0	-1.772486	1.748399	0.432818
6	0	-0.609898	1.547816	1.125841
6	0	0.059213	0.530719	-2.263611
6	0	-0.605906	-0.397992	-1.571742
1	0	0.086638	3.666183	-1.310321
1	0	1.443030	2.402811	0.737906
1	0	-0.480981	0.911022	1.996540
8	0	-1.551644	2.617397	-0.587797
6	0	1.490799	0.536967	-1.814302
6	0	0.372828	-1.071484	-0.649230
1	0	-1.655196	-0.690667	-1.595577
1	0	-0.304541	1.210461	-3.029874
7	0	1.589202	-0.445281	-0.814059
8	0	0.167284	-2.015949	0.095570
8	0	2.406580	1.211018	-2.208644
6	0	2.850332	-0.828223	-0.187121
6	0	-3.174814	1.239093	0.562168
1	0	-3.824349	1.894835	-0.031600
6	0	2.740310	-0.832087	1.331250
1	0	3.730336	-1.103437	1.747258
6	0	3.333628	-2.177421	-0.711755
1	0	2.612063	-2.966968	-0.457631
1	0	3.447402	-2.140631	-1.803350
1	0	4.307803	-2.433648	-0.271817
1	0	3.550586	-0.032316	-0.479308
8	0	2.357287	0.447621	1.769455
6	0	2.236957	0.513132	3.168029
1	0	3.202825	0.312064	3.666826
1	0	1.906256	1.528116	3.423753
1	0	1.494132	-0.215676	3.542187
1	0	2.007745	-1.591313	1.659283
8	0	-3.366798	-0.072221	0.074401
6	0	-2.976203	-1.064119	1.001453
1	0	-1.895411	-0.985233	1.228419

1	0	-3.538275	-0.934745	1.947904
6	0	-3.271555	-2.429649	0.412670
1	0	-4.314885	-2.443094	0.064491
1	0	-3.173634	-3.181928	1.218086
1	0	-3.492344	1.296625	1.618745
8	0	-2.463649	-2.740576	-0.694744
1	0	-1.541212	-2.730113	-0.384042

- Transition state:

6	0	0.170066	1.314958	1.757095
6	0	0.088558	2.632614	1.231399
6	0	-1.482448	1.523611	0.271494
6	0	-0.821809	0.600752	1.128082
6	0	1.000431	2.321451	-0.608750
6	0	0.026708	1.543027	-1.259130
1	0	0.533829	3.544382	1.621320
1	0	0.983079	0.910711	2.354028
1	0	-0.964188	-0.477896	1.111261
8	0	-1.134593	2.786695	0.635201
6	0	2.146850	1.420846	-0.275865
6	0	0.539972	0.147244	-1.323220
1	0	-0.657691	1.889411	-2.029822
1	0	1.199942	3.371812	-0.809045
7	0	1.753056	0.127800	-0.651963
8	0	0.025061	-0.831068	-1.838560
8	0	3.205159	1.693136	0.235732
6	0	2.554016	-1.062650	-0.381440
6	0	-2.807265	1.364221	-0.423952
1	0	-2.864929	2.060579	-1.272325
6	0	1.762798	-2.089211	0.418363
1	0	2.419334	-2.962045	0.604675
6	0	3.102944	-1.661201	-1.673360
1	0	2.279204	-1.993572	-2.319886
1	0	3.697437	-0.912246	-2.213460
1	0	3.750605	-2.520709	-1.449397
1	0	3.377969	-0.696158	0.247835
8	0	1.358011	-1.512762	1.637530
6	0	0.573280	-2.408383	2.393912
1	0	1.155302	-3.304036	2.679905
1	0	0.262271	-1.881887	3.305959
1	0	-0.317416	-2.725202	1.823189
1	0	0.885179	-2.439341	-0.154372
8	0	-2.938394	0.056271	-0.911402
6	0	-3.576176	-0.844421	-0.019905
1	0	-3.309299	-0.608870	1.025708
1	0	-4.674715	-0.776293	-0.126807
6	0	-3.081016	-2.234602	-0.364575
1	0	-3.367676	-2.472426	-1.404408
1	0	-3.564686	-2.967898	0.297184
1	0	-3.623413	1.624780	0.274805
8	0	-1.689232	-2.340289	-0.179244
1	0	-1.261068	-1.783091	-0.857540

- Endo product:

6	0	0.030746	1.439113	1.773672

6	0	0.097801	2.706065	0.937396
6	0	-1.372768	1.481809	-0.017063
6	0	-0.879773	0.668008	1.173164
6	0	0.850738	2.320747	-0.391991
6	0	-0.197558	1.433058	-1.075451
1	0	0.432421	3.632995	1.408951
1	0	0.724486	1.163375	2.565453
1	0	-1.093333	-0.388030	1.337366
8	0	-1.230600	2.822538	0.443195
6	0	2.056435	1.425933	-0.183217
6	0	0.477374	0.084211	-1.228955
1	0	-0.549329	1.798431	-2.047912
1	0	1.126785	3.219849	-0.953975
7	0	1.752905	0.163498	-0.706610
8	0	0.001309	-0.924464	-1.712780
8	0	3.097763	1.711165	0.349929
6	0	2.631100	-0.993339	-0.513024
6	0	-2.769565	1.238793	-0.549430
1	0	-2.935405	1.866380	-1.438096
6	0	1.947730	-2.029618	0.372832
1	0	2.672584	-2.842504	0.577601
6	0	3.080468	-1.583671	-1.844320
1	0	2.217559	-1.957474	-2.411368
1	0	3.598959	-0.823644	-2.443911
1	0	3.776709	-2.415565	-1.667286
1	0	3.490479	-0.584929	0.036845
8	0	1.550099	-1.400415	1.563974
6	0	0.799144	-2.266679	2.385849
1	0	1.402269	-3.136703	2.705614
1	0	0.504030	-1.696014	3.276389
1	0	-0.102602	-2.624838	1.858744
1	0	1.076507	-2.471233	-0.144348
8	0	-2.919511	-0.109969	-0.921962
6	0	-3.587816	-0.923899	0.025918
1	0	-3.363580	-0.586646	1.053267
1	0	-4.682129	-0.876310	-0.126213
6	0	-3.072543	-2.336755	-0.163416
1	0	-3.337517	-2.687052	-1.177072
1	0	-3.554743	-3.005517	0.564248
1	0	-3.499095	1.539436	0.221068
8	0	-1.681363	-2.397159	0.051538
1	0	-1.266048	-1.864725	-0.652002

Furan + Maleimide (HB: maleimide C=O + furan -OH, conformation 1): Exo

- Reactant complex:

6	0	-2.487558	2.493237	-0.367479
6	0	-2.199661	1.418573	0.420482
6	0	-0.512374	2.797775	0.623318
6	0	-1.383535	3.394918	-0.237550
1	0	-3.377236	2.608715	-0.978233
1	0	-1.251970	4.360248	-0.716068
6	0	-0.294120	0.070937	-1.553996
1	0	-1.239831	-0.236299	-1.992607
6	0	0.524128	1.077273	-1.869194
1	0	0.444969	1.811234	-2.667022

1	0	0.450600	3.085841	1.032641
8	0	-0.998570	1.601906	1.032662
6	0	1.659370	1.100333	-0.884279
6	0	0.267309	-0.621111	-0.344458
7	0	1.428228	0.047641	-0.000031
8	0	-0.185962	-1.575545	0.251203
8	0	2.589633	1.868718	-0.832701
6	0	-2.908597	0.131809	0.677861
1	0	-3.865203	0.318545	1.200412
1	0	-2.286905	-0.500062	1.328072
8	0	-3.170407	-0.480682	-0.572747
6	0	2.276614	-0.321950	1.126350
1	0	1.824166	-1.245394	1.514652
6	0	2.270091	0.763693	2.196695
1	0	1.241242	0.950221	2.534246
1	0	2.870304	0.450836	3.062962
1	0	2.693038	1.696312	1.795669
6	0	4.830179	-1.960355	-0.903673
1	0	4.661944	-2.714180	-1.682314
1	0	5.323017	-1.078926	-1.353564
1	0	5.505231	-2.382011	-0.135749
6	0	-3.977500	-1.645226	-0.486956
1	0	-4.719580	-1.531582	0.324404
1	0	-4.515053	-1.721183	-1.445017
6	0	-3.157701	-2.902210	-0.256143
1	0	-3.830511	-3.774160	-0.325337
1	0	-2.409876	-2.991148	-1.064333
8	0	-2.544392	-2.915516	1.011190
1	0	-1.655446	-2.549267	0.887054
8	0	3.578920	-1.625345	-0.361541
6	0	3.682749	-0.650292	0.642276
1	0	4.274414	-1.025153	1.501030
1	0	4.174960	0.261211	0.255274

- Transition state:

6	0	2.468299	-2.285111	-0.541626
6	0	1.900819	-1.247571	0.244797
6	0	0.528579	-2.899828	0.366491
6	0	1.607234	-3.351531	-0.441754
1	0	3.348112	-2.166323	-1.166644
1	0	1.635542	-4.294244	-0.981164
6	0	0.383882	-0.701834	-1.259200
1	0	1.041902	-0.489601	-2.096029
6	0	-0.467694	-1.806823	-1.098512
1	0	-0.650243	-2.588026	-1.831737
1	0	-0.250512	-3.477191	0.859443
8	0	0.932050	-1.776566	1.031210
6	0	-1.609283	-1.370203	-0.229344
6	0	-0.222498	0.432199	-0.514260
7	0	-1.346041	-0.055164	0.144584
8	0	0.167026	1.582428	-0.436084
8	0	-2.566372	-2.018759	0.130140
6	0	2.574153	0.008048	0.697841
1	0	3.313259	-0.235429	1.486706
1	0	1.844237	0.709021	1.126940
8	0	3.217044	0.519537	-0.442911

6	0	-2.169766	0.743338	1.044006
1	0	-1.685832	1.730175	1.052813
6	0	-2.194514	0.142616	2.445145
1	0	-1.170111	0.031736	2.826043
1	0	-2.754015	0.795118	3.130271
1	0	-2.671350	-0.847214	2.427769
6	0	-4.683156	1.607142	-1.458116
1	0	-4.496659	2.023868	-2.455437
1	0	-5.200853	0.635785	-1.562751
1	0	-5.345860	2.295091	-0.900235
6	0	3.962437	1.704964	-0.208619
1	0	4.440552	1.662391	0.787314
1	0	4.750977	1.725211	-0.976171
6	0	3.111551	2.957952	-0.315255
1	0	3.783739	3.833104	-0.295285
1	0	2.594386	2.947546	-1.289875
8	0	2.196081	3.078656	0.749158
1	0	1.356251	2.712423	0.430819
6	0	-3.568206	0.915371	0.465371
1	0	-4.145205	1.592165	1.126875
1	0	-4.086763	-0.060488	0.430577
8	0	-3.440866	1.459535	-0.822026

- Exo product:

6	0	-2.289699	2.388355	-0.691175
6	0	-1.692955	1.210794	0.071595
6	0	-0.295622	2.807882	0.303166
6	0	-1.425432	3.392283	-0.532280
1	0	-3.195557	2.332107	-1.290893
1	0	-1.440834	4.387316	-0.972594
6	0	-0.503494	0.702191	-0.826875
1	0	-0.825589	0.467944	-1.845901
6	0	0.500319	1.855027	-0.648845
1	0	0.847791	2.348888	-1.562635
1	0	0.324274	3.478900	0.904418
8	0	-0.974638	1.855088	1.118790
6	0	1.674622	1.242524	0.092176
6	0	0.190479	-0.499908	-0.214975
7	0	1.405611	-0.102990	0.310062
8	0	-0.233324	-1.635705	-0.158268
8	0	2.669744	1.816495	0.464790
6	0	-2.661588	0.170764	0.574340
1	0	-3.423515	0.664758	1.205938
1	0	-2.147260	-0.588307	1.182423
8	0	-3.241411	-0.380536	-0.586138
6	0	2.326190	-1.031931	0.965494
1	0	1.842436	-2.011959	0.855456
6	0	2.506608	-0.684833	2.438368
1	0	1.538217	-0.703140	2.955658
1	0	3.169933	-1.418632	2.917889
1	0	2.947119	0.315725	2.544938
6	0	4.545149	-1.286174	-1.924707
1	0	4.245714	-1.438030	-2.968832
1	0	5.095463	-0.331134	-1.840437
1	0	5.222859	-2.106238	-1.623092
6	0	-4.144578	-1.447004	-0.339231

1	0	-4.650015	-1.300591	0.632296
1	0	-4.902259	-1.408120	-1.137380
6	0	-3.446274	-2.795016	-0.359103
1	0	-4.212416	-3.586159	-0.297686
1	0	-2.925520	-2.904673	-1.327082
8	0	-2.562081	-2.961335	0.723295
1	0	-1.704892	-2.602703	0.444353
8	0	3.375942	-1.272278	-1.146448
6	0	3.652054	-1.065121	0.214688
1	0	4.265796	-1.890659	0.626106
1	0	4.200452	-0.117196	0.362641

Furan + Maleimide (HB: maleimide C=O + furan -OH, conformation 2): Exo

- Reactant complex:

6	0	-1.168824	2.952126	-0.050631
6	0	-1.189111	1.731393	0.556360
6	0	0.922439	2.287341	0.368111
6	0	0.210243	3.314738	-0.176056
1	0	-2.040013	3.509887	-0.379578
1	0	0.623002	4.218897	-0.612461
6	0	-0.413477	0.084808	-1.879138
1	0	-1.483918	0.204389	-2.026587
6	0	0.615508	0.765962	-2.388162
1	0	0.618618	1.584464	-3.103352
1	0	1.978941	2.078545	0.501934
8	0	0.082383	1.325302	0.821237
6	0	1.887178	0.233969	-1.790466
6	0	0.131028	-0.924400	-0.908498
7	0	1.505222	-0.793875	-0.915748
8	0	-0.491848	-1.720378	-0.230964
8	0	3.019979	0.582769	-1.997733
6	0	-2.292544	0.796858	0.920297
1	0	-2.971886	1.272795	1.651420
1	0	-1.865830	-0.101814	1.389404
8	0	-3.020074	0.485530	-0.256825
6	0	2.440128	-1.541145	-0.078414
1	0	3.429971	-1.156674	-0.364646
6	0	2.352673	-3.040038	-0.347320
1	0	2.492863	-3.241344	-1.417759
1	0	3.139204	-3.567855	0.209541
1	0	1.374301	-3.434088	-0.041205
6	0	2.307820	0.544053	2.922443
1	0	2.634433	1.587234	3.020626
1	0	1.222902	0.485200	3.120104
1	0	2.843296	-0.071346	3.668448
6	0	-4.174303	-0.308329	-0.025984
1	0	-4.607455	-0.067567	0.961799
1	0	-4.904150	-0.035014	-0.804111
6	0	-3.869326	-1.793867	-0.102428
1	0	-4.817531	-2.351049	-0.016001
1	0	-3.441442	-2.017289	-1.096865
8	0	-3.013308	-2.218138	0.930743
1	0	-2.108040	-2.123351	0.594378
8	0	2.605635	0.127089	1.611432
6	0	2.222007	-1.207368	1.390482

1	0	2.835384	-1.891043	2.009277
1	0	1.160027	-1.359850	1.659166

- Transition state:

6	0	-0.372850	3.187322	0.150762
6	0	-0.439363	1.813719	0.504615
6	0	1.593223	2.415247	0.864700
6	0	0.917848	3.583014	0.412005
1	0	-1.178773	3.735901	-0.327224
1	0	1.391161	4.532835	0.179037
6	0	0.613830	1.172199	-1.366120
1	0	0.007086	1.674644	-2.112950
6	0	1.913109	1.511842	-0.952985
1	0	2.557113	2.254131	-1.418297
1	0	2.546877	2.330294	1.381272
8	0	0.652498	1.493538	1.228849
6	0	2.547133	0.252951	-0.428325
6	0	0.448498	-0.285951	-1.172882
7	0	1.585328	-0.755046	-0.532833
8	0	-0.491825	-0.991052	-1.505076
8	0	3.655153	0.101250	0.026485
6	0	-1.651182	0.958684	0.676075
1	0	-2.124182	1.194271	1.651225
1	0	-1.355842	-0.098373	0.692089
8	0	-2.517588	1.278899	-0.384203
6	0	1.793632	-2.106328	-0.025653
1	0	2.645281	-2.005966	0.662270
6	0	2.145673	-3.066677	-1.157199
1	0	2.303267	-4.081915	-0.764726
1	0	1.329552	-3.095567	-1.893561
1	0	3.067249	-2.742185	-1.658409
6	0	-0.917820	-2.031759	2.447075
1	0	-1.134163	-1.224694	3.158984
1	0	-1.806585	-2.213229	1.816355
1	0	-0.675206	-2.950124	3.012501
6	0	-3.751727	0.581291	-0.338730
1	0	-4.081814	0.462703	0.710127
1	0	-4.482767	1.214323	-0.864573
6	0	-3.685408	-0.777800	-1.013779
1	0	-4.708194	-1.187564	-1.066800
1	0	-3.317777	-0.639821	-2.044660
8	0	-2.878570	-1.700603	-0.312963
1	0	-1.988960	-1.624408	-0.697503
6	0	0.587210	-2.607309	0.763098
1	0	0.894188	-3.524144	1.304239
1	0	-0.238717	-2.882007	0.081884
8	0	0.180716	-1.612838	1.665985

- Exo product:

6	0	-0.840296	3.170207	-0.000197
6	0	-0.612386	1.681543	0.237310
6	0	1.270429	2.608357	0.622247
6	0	0.338083	3.749957	0.241463
1	0	-1.764052	3.599240	-0.382879
1	0	0.627002	4.791268	0.113359

6	0	0.216202	1.218018	-1.030254
1	0	-0.257894	1.536564	-1.962808
6	0	1.590956	1.818044	-0.687055
1	0	2.072514	2.420276	-1.465515
1	0	2.139784	2.817020	1.251878
8	0	0.385870	1.684326	1.247141
6	0	2.447118	0.612209	-0.325982
6	0	0.440386	-0.276818	-1.039624
7	0	1.708651	-0.545367	-0.578468
8	0	-0.354563	-1.130203	-1.387704
8	0	3.564703	0.624595	0.121749
6	0	-1.787184	0.825495	0.630712
1	0	-2.240636	1.242396	1.549799
1	0	-1.439811	-0.196427	0.846239
8	0	-2.693080	0.869166	-0.447392
6	0	2.167017	-1.875256	-0.176687
1	0	3.061213	-1.673396	0.429058
6	0	2.525623	-2.731524	-1.384551
1	0	3.342992	-2.271414	-1.955408
1	0	2.852832	-3.728461	-1.055334
1	0	1.650092	-2.846952	-2.038872
6	0	-0.403398	-2.143161	2.428154
1	0	-0.703769	-1.346968	3.120876
1	0	-1.280807	-2.463503	1.837934
1	0	-0.019724	-3.000505	3.010855
6	0	-3.838402	0.053119	-0.263245
1	0	-4.090770	-0.009930	0.810866
1	0	-4.670134	0.550113	-0.786752
6	0	-3.644957	-1.341820	-0.832120
1	0	-4.598776	-1.888959	-0.751433
1	0	-3.393930	-1.252909	-1.903479
8	0	-2.660175	-2.082272	-0.144677
1	0	-1.811383	-1.853489	-0.558501
8	0	0.599948	-1.604545	1.595009
6	0	1.115069	-2.555304	0.702765
1	0	1.607999	-3.385037	1.246713
1	0	0.309520	-2.988287	0.082058

Furan + Maleimide (HB: furan (O) + glycol, conformation 1): Endo

- Reactant 1 + glycol:

6	0	-4.303476	0.288474	-0.586805
6	0	-3.373755	1.275914	-0.648737
6	0	-2.373579	-0.435466	0.291060
6	0	-3.647212	-0.831676	0.027353
1	0	-3.386075	2.292838	-1.024857
1	0	-5.328712	0.346944	-0.937088
1	0	-4.065901	-1.810103	0.241114
8	0	-2.197964	0.853745	-0.117399
6	0	-1.196281	-1.101824	0.905441
1	0	-0.767599	-0.452502	1.688814
1	0	-1.526915	-2.048385	1.369538
8	0	-0.226057	-1.338200	-0.094973
6	0	1.041247	-1.614742	0.455460
1	0	1.393528	-0.745946	1.040494
1	0	0.989132	-2.489861	1.133868

6	0	1.999956	-1.864414	-0.704537
1	0	1.979399	-2.919431	-1.023651
1	0	1.666516	-1.254820	-1.554948
8	0	3.323609	-1.446369	-0.412693
1	0	3.663863	-1.967484	0.326157
8	0	0.434478	1.610872	0.944969
1	0	-0.459475	1.620896	0.575701
6	0	1.344292	1.611610	-0.138429
1	0	1.214561	0.710507	-0.765141
1	0	1.205323	2.489140	-0.795284
6	0	2.755649	1.629849	0.432420
1	0	2.959825	2.617135	0.873652
1	0	2.809306	0.893693	1.257612
8	0	3.721456	1.387991	-0.558634
1	0	3.727997	0.426723	-0.695509

- Reactant complex:

6	0	0.926661	-2.862757	1.005745
6	0	-0.022402	-2.343807	1.828956
6	0	-0.581212	-1.724034	-0.199709
6	0	0.554036	-2.463894	-0.321794
6	0	1.540268	0.518643	1.809949
6	0	0.793389	0.921152	0.779841
1	0	-0.188386	-2.389237	2.899672
1	0	1.782780	-3.458734	1.306110
1	0	1.061283	-2.688891	-1.255066
8	0	-0.946036	-1.652663	1.112103
6	0	2.829709	-0.045211	1.282003
6	0	1.546091	0.621794	-0.488330
1	0	-0.199820	1.372119	0.784854
1	0	1.330102	0.563900	2.876016
7	0	2.743330	0.016569	-0.104583
8	0	1.197267	0.826561	-1.625431
8	0	3.770186	-0.465464	1.914244
6	0	3.843072	-0.242856	-1.024906
1	0	3.426148	-0.016154	-2.016989
6	0	-1.401419	-0.956825	-1.176060
1	0	-1.250900	0.127234	-1.026987
1	0	-1.050211	-1.201978	-2.194369
8	0	-2.764749	-1.277128	-0.998506
6	0	-3.590402	-0.337867	-1.647887
1	0	-3.425885	0.664396	-1.212571
1	0	-3.344450	-0.286011	-2.726765
6	0	-5.044449	-0.740935	-1.424400
1	0	-5.401034	-1.427130	-2.209477
1	0	-5.105539	-1.274876	-0.466820
8	0	-5.899200	0.387261	-1.311708
1	0	-5.904014	0.858620	-2.155442
6	0	4.312534	-1.691332	-0.956137
1	0	4.676625	-1.928021	0.052767
1	0	3.489109	-2.374545	-1.202855
6	0	4.981846	0.733988	-0.753566
1	0	5.760111	0.606077	-1.531924
1	0	5.438916	0.515463	0.230474
8	0	-2.353008	0.902341	1.156148
1	0	-2.022574	0.009223	1.336426

6	0	-3.741516	0.909529	1.433049
1	0	-4.242580	0.064964	0.927714
1	0	-3.944719	0.813526	2.514421
6	0	-4.347139	2.207418	0.919284
1	0	-4.017919	3.048966	1.546610
1	0	-3.963227	2.398746	-0.101760
8	0	-5.749783	2.161899	0.965882
1	0	-6.017370	1.524585	0.282794
1	0	5.125914	-1.858963	-1.676632
8	0	4.451998	2.032929	-0.778512
6	0	5.419530	3.006983	-0.483754
1	0	6.239307	3.001988	-1.226030
1	0	4.922463	3.984233	-0.508501
1	0	5.857565	2.848691	0.518937

- Transition state:

6	0	0.497565	-1.327619	2.270297
6	0	-0.040184	-0.032428	2.461109
6	0	-0.978399	-0.799371	0.672598
6	0	-0.103829	-1.817561	1.133047
6	0	1.063226	1.021582	0.936150
6	0	0.417754	0.519673	-0.199050
1	0	0.011538	0.601330	3.342186
1	0	1.319694	-1.755296	2.837619
1	0	0.122808	-2.728743	0.586051
8	0	-1.174324	0.079647	1.711366
6	0	2.429054	0.417989	0.983724
6	0	1.358370	-0.418403	-0.880300
1	0	-0.334761	1.062649	-0.768211
1	0	0.889542	2.001240	1.375513
7	0	2.494499	-0.494699	-0.070010
8	0	1.185620	-1.049785	-1.896890
8	0	3.321133	0.626950	1.776335
6	0	3.662232	-1.296506	-0.410020
1	0	3.376538	-1.809923	-1.339524
6	0	-2.049351	-0.918474	-0.359616
1	0	-2.542418	0.062983	-0.481506
1	0	-1.558888	-1.182204	-1.316472
8	0	-2.964430	-1.915223	0.029987
6	0	-3.978735	-2.101370	-0.928134
1	0	-4.548818	-1.168205	-1.096226
1	0	-3.539946	-2.402235	-1.902475
6	0	-4.911094	-3.186246	-0.418422
1	0	-4.324608	-4.099472	-0.212752
1	0	-5.356557	-2.859481	0.531049
8	0	-5.975362	-3.413308	-1.314086
1	0	-5.606125	-3.804017	-2.116287
6	0	3.983429	-2.305948	0.686490
1	0	4.203739	-1.785506	1.629192
1	0	3.132391	-2.983450	0.843007
6	0	4.850994	-0.392494	-0.716043
1	0	5.689181	-1.018179	-1.083052
1	0	5.179901	0.124540	0.204147
8	0	-1.975850	2.374556	0.210935
1	0	-1.902412	1.799375	0.986448
6	0	-2.801402	3.479405	0.520161

1	0	-3.824583	3.167756	0.793639
1	0	-2.393727	4.077821	1.353759
6	0	-2.869745	4.352800	-0.713813
1	0	-1.843788	4.658956	-0.988059
1	0	-3.279355	3.756604	-1.549633
8	0	-3.690018	5.453932	-0.388600
1	0	-3.744592	6.025747	-1.162751
1	0	4.856621	-2.911690	0.404468
8	0	4.451264	0.533677	-1.691661
6	0	5.461493	1.463166	-1.983159
1	0	6.365776	0.968223	-2.384512
1	0	5.068086	2.152548	-2.740160
1	0	5.750729	2.038768	-1.084586

- *Endo product + glycol:

6	0	0.214179	-1.569347	2.110271
6	0	-0.004010	-0.066123	2.190569
6	0	-0.952916	-0.682125	0.364051
6	0	-0.382541	-1.952908	0.979092
6	0	0.926125	0.612022	1.121722
6	0	0.255510	0.160837	-0.177973
1	0	-0.005174	0.400839	3.178289
1	0	0.815078	-2.158260	2.800422
1	0	-0.403014	-2.933617	0.508532
8	0	-1.273648	0.077854	1.544505
6	0	2.334153	0.051828	1.053597
6	0	1.300571	-0.653811	-0.906822
1	0	-0.113239	0.989245	-0.795180
1	0	0.941611	1.699439	1.257171
7	0	2.463170	-0.648992	-0.139198
8	0	1.163837	-1.249508	-1.948065
8	0	3.199636	0.154401	1.892057
6	0	3.671768	-1.359855	-0.550802
1	0	3.443798	-1.709392	-1.567442
6	0	-2.097831	-0.796830	-0.601451
1	0	-2.527777	0.210903	-0.757604
1	0	-1.688323	-1.151597	-1.567771
8	0	-3.045428	-1.694373	-0.088296
6	0	-4.156132	-1.845546	-0.937558
1	0	-4.667516	-0.878833	-1.104036
1	0	-3.836263	-2.227269	-1.929973
6	0	-5.115265	-2.826102	-0.286052
1	0	-4.583293	-3.773305	-0.085276
1	0	-5.440987	-2.419500	0.680976
8	0	-6.274801	-3.008840	-1.066343
1	0	-6.014442	-3.462796	-1.877877
6	0	3.953963	-2.542935	0.368326
1	0	4.129790	-2.195813	1.395908
1	0	3.101100	-3.235608	0.366864
6	0	4.849320	-0.396642	-0.629022
1	0	5.711677	-0.933557	-1.072038
1	0	5.134728	-0.056881	0.382954
8	0	-1.623723	2.487189	0.187730
1	0	-1.706784	1.796023	0.867242
6	0	-2.460750	3.575437	0.515333
1	0	-3.523104	3.279401	0.573096

1	0	-2.185362	4.033642	1.481937
6	0	-2.303107	4.614649	-0.573482
1	0	-1.237608	4.902896	-0.635599
1	0	-2.586547	4.161424	-1.541107
8	0	-3.133239	5.702591	-0.228089
1	0	-3.049596	6.372921	-0.915660
1	0	4.842802	-3.089042	0.021417
8	0	4.468399	0.690917	-1.430840
6	0	5.483982	1.655907	-1.525943
1	0	6.397079	1.239248	-1.990618
1	0	5.106554	2.472634	-2.153247
1	0	5.752234	2.057224	-0.531405

- Endo product:

6	0	-0.449775	1.896694	1.123917
6	0	-0.288070	2.353254	-0.319526
6	0	-1.352710	0.505903	-0.437738
6	0	-1.115507	0.741954	1.049761
6	0	0.797881	1.425980	-0.983368
6	0	0.028284	0.106242	-1.075084
1	0	-0.169057	3.421923	-0.512284
1	0	-0.015414	2.381308	1.996288
1	0	-1.376607	0.043166	1.841813
8	0	-1.464549	1.839369	-0.934822
6	0	2.002067	1.116398	-0.114084
6	0	0.828374	-0.885435	-0.259502
1	0	-0.123048	-0.267414	-2.095474
1	0	1.119366	1.837005	-1.946655
7	0	1.942220	-0.224061	0.249412
8	0	0.546485	-2.038440	-0.037639
8	0	2.861830	1.889290	0.240219
6	0	2.920896	-0.890377	1.105910
1	0	2.612056	-1.944991	1.094994
6	0	-2.492835	-0.388541	-0.836672
1	0	-2.698193	-0.246706	-1.915071
1	0	-2.175981	-1.438268	-0.678371
8	0	-3.616708	-0.075374	-0.059339
6	0	-4.730036	-0.874444	-0.373675
1	0	-5.022569	-0.756316	-1.434067
1	0	-4.495684	-1.947614	-0.211054
6	0	-5.880041	-0.449987	0.522769
1	0	-5.564848	-0.531129	1.578466
1	0	-6.114123	0.605443	0.328573
8	0	-7.048662	-1.188048	0.246110
1	0	-6.890400	-2.101414	0.516613
6	0	2.877732	-0.337011	2.525704
1	0	3.134722	0.731255	2.528149
1	0	1.872663	-0.462783	2.951173
6	0	4.311495	-0.793010	0.491162
1	0	5.001109	-1.425946	1.084392
1	0	4.676378	0.249219	0.530284
1	0	3.592828	-0.875432	3.163976
8	0	4.232413	-1.243174	-0.836423
6	0	5.468822	-1.155103	-1.496307
1	0	6.235982	-1.782734	-1.005667
1	0	5.321913	-1.510993	-2.523317

1	0	5.837432	-0.113227	-1.523100

Furan + Maleimide (HB: furan (O) + glycol, conformation 2): Endo

- Reactant 1 + glycol:

6	0	-4.216812	0.157355	-0.661328
6	0	-3.341572	1.193729	-0.601581
6	0	-2.295195	-0.528731	0.264462
6	0	-3.530500	-0.969431	-0.094486
1	0	-3.392920	2.234298	-0.902352
1	0	-5.225064	0.188407	-1.060965
1	0	-3.903745	-1.981649	0.025688
8	0	-2.172073	0.795635	-0.039362
6	0	-1.111261	-1.179945	0.882230
1	0	-0.706208	-0.526862	1.674869
1	0	-1.425213	-2.137300	1.334830
8	0	-0.121842	-1.388726	-0.107287
6	0	1.158121	-1.561771	0.453784
1	0	1.406797	-0.700945	1.100366
1	0	1.195331	-2.479566	1.076519
6	0	2.157938	-1.638918	-0.690514
1	0	2.076375	-2.601311	-1.222391
1	0	1.939918	-0.834356	-1.405245
8	0	3.483070	-1.399044	-0.243393
1	0	3.740176	-2.091443	0.378501
8	0	0.347816	1.713467	1.156301
1	0	-0.588062	1.714771	0.913619
6	0	1.083144	1.696320	-0.055902
1	0	0.828934	0.805775	-0.655179
1	0	0.868184	2.584658	-0.677487
6	0	2.563822	1.675065	0.300956
1	0	2.858970	2.661885	0.694521
1	0	2.716465	0.947326	1.116988
8	0	3.351783	1.364404	-0.827328
1	0	3.693908	0.468385	-0.689460

- Reactant complex:

6	0	-1.388233	1.116898	1.976706
6	0	-0.358905	0.453717	2.567689
6	0	0.346418	1.006246	0.565476
6	0	-0.921171	1.486542	0.672287
6	0	-1.513216	-2.097282	0.861058
6	0	-0.682097	-1.803641	-0.141090
1	0	-0.221531	0.004293	3.545419
1	0	-2.365092	1.312297	2.407678
1	0	-1.486082	2.000158	-0.098835
8	0	0.703532	0.373676	1.724062
6	0	-2.867448	-1.520066	0.552835
6	0	-1.437865	-0.984985	-1.150970
1	0	0.368754	-2.067987	-0.264175
1	0	-1.329876	-2.668361	1.768188
7	0	-2.738620	-0.851030	-0.661311
8	0	-1.032009	-0.522708	-2.190019
8	0	-3.881547	-1.623774	1.201267
6	0	-3.822533	-0.243840	-1.427501

1	0	-3.313341	0.326699	-2.217999
6	0	1.316465	0.978812	-0.559311
1	0	1.562408	-0.069862	-0.811889
1	0	0.836639	1.436886	-1.441416
8	0	2.502740	1.657809	-0.192019
6	0	3.534716	1.419707	-1.115316
1	0	3.625797	0.335934	-1.320192
1	0	3.327065	1.923017	-2.081211
6	0	4.848766	1.891589	-0.512225
1	0	4.938432	2.989562	-0.544626
1	0	4.884147	1.582079	0.541277
8	0	5.951008	1.259507	-1.143205
1	0	5.969012	1.518316	-2.074170
6	0	-4.642644	0.726218	-0.587884
1	0	-5.146582	0.188496	0.233724
6	0	-4.713136	-1.319455	-2.044551
1	0	-5.503933	-0.862961	-2.656663
1	0	-5.183306	-1.922550	-1.254056
8	0	2.343065	-1.836502	0.814198
1	0	1.749209	-1.292378	1.351602
6	0	3.648199	-1.306696	0.989027
1	0	3.628740	-0.205960	0.903379
1	0	4.058008	-1.556207	1.983986
6	0	4.559953	-1.902076	-0.073160
1	0	4.607650	-2.992144	0.072546
1	0	4.108309	-1.736283	-1.070431
8	0	5.867319	-1.396323	0.027247
1	0	5.915581	-0.542686	-0.434083
1	0	-5.418371	1.170860	-1.241729
8	0	-3.799828	1.729227	-0.077150
6	0	-4.496510	2.641756	0.734077
1	0	-3.769343	3.376232	1.104034
1	0	-5.283100	3.169908	0.164462
1	0	-4.969969	2.133246	1.593839
1	0	-4.117683	-1.981372	-2.687413

- Transition state:

6	0	1.250732	0.495249	-1.839793
6	0	0.317327	-0.532932	-2.113722
6	0	-0.527480	0.612488	-0.488973
6	0	0.712210	1.226207	-0.807087
6	0	0.761139	-1.809423	-0.440303
6	0	0.174836	-1.053520	0.583409
1	0	0.262836	-1.193772	-2.974710
1	0	2.254105	0.564151	-2.250248
1	0	1.205561	1.993017	-0.216578
8	0	-0.876772	-0.196539	-1.545607
6	0	2.243515	-1.700762	-0.291100
6	0	1.273998	-0.451414	1.392422
1	0	-0.788245	-1.279990	1.037650
1	0	0.326197	-2.699980	-0.888238
7	0	2.471133	-0.822552	0.771743
8	0	1.192234	0.250916	2.373973
8	0	3.113470	-2.225684	-0.951428
6	0	3.778746	-0.348317	1.208527
1	0	3.555585	0.357182	2.022079

6	0	-1.623088	1.154482	0.366815
1	0	-2.437002	0.409030	0.425100
1	0	-1.208937	1.297844	1.383400
8	0	-2.076043	2.374454	-0.170848
6	0	-3.086900	2.954134	0.618132
1	0	-3.961985	2.282909	0.707658
1	0	-2.712347	3.149634	1.644752
6	0	-3.505907	4.256386	-0.041029
1	0	-2.617373	4.901055	-0.163981
1	0	-3.898387	4.040722	-1.044131
8	0	-4.541226	4.888076	0.677262
1	0	-4.176539	5.180136	1.522374
6	0	4.480502	0.419606	0.097050
1	0	4.626036	-0.234659	-0.783048
6	0	4.641031	-1.497025	1.724807
1	0	5.593703	-1.112489	2.115942
1	0	4.846740	-2.213865	0.918305
8	0	-2.617788	-2.004088	-0.185944
1	0	-2.241880	-1.513752	-0.931849
6	0	-3.752635	-2.725773	-0.620255
1	0	-4.541836	-2.060300	-1.011616
1	0	-3.500120	-3.454897	-1.410136
6	0	-4.295446	-3.477401	0.575828
1	0	-3.500917	-4.137374	0.969221
1	0	-4.552437	-2.749409	1.366934
8	0	-5.421013	-4.200580	0.127154
1	0	-5.781709	-4.688482	0.876224
1	0	5.477670	0.734442	0.461422
8	0	3.708229	1.547931	-0.243905
6	0	4.329688	2.327538	-1.233882
1	0	3.660095	3.165193	-1.467430
1	0	5.298984	2.727545	-0.883921
1	0	4.511362	1.742030	-2.155129
1	0	4.120553	-2.021084	2.537430

- Endo product + glycol:

6	0	1.044714	0.733959	-1.837005
6	0	0.243269	-0.553472	-1.929242
6	0	-0.561009	0.498300	-0.237553
6	0	0.543622	1.388037	-0.787396
6	0	0.709818	-1.477721	-0.744763
6	0	0.137111	-0.724954	0.458476
1	0	0.155904	-1.043429	-2.901829
1	0	1.920402	0.977270	-2.435131
1	0	0.909106	2.293492	-0.308406
8	0	-1.035345	-0.139856	-1.437466
6	0	2.210238	-1.494697	-0.518978
6	0	1.332572	-0.343576	1.302477
1	0	-0.600297	-1.299708	1.032019
1	0	0.313297	-2.492591	-0.863320
7	0	2.476551	-0.855649	0.687875
8	0	1.327800	0.303917	2.320678
8	0	3.051748	-1.951291	-1.258038
6	0	3.821527	-0.536421	1.166413
1	0	3.649459	0.033386	2.090365
6	0	-1.656685	1.119344	0.580820

1	0	-2.470615	0.378642	0.699145
1	0	-1.242195	1.340985	1.583991
8	0	-2.103460	2.285942	-0.056184
6	0	-3.133148	2.925761	0.655984
1	0	-4.007961	2.260126	0.781031
1	0	-2.784222	3.209595	1.671220
6	0	-3.540104	4.165606	-0.120613
1	0	-2.650294	4.801000	-0.278617
1	0	-3.909452	3.863494	-1.109980
8	0	-4.592119	4.852723	0.518300
1	0	-4.248188	5.211543	1.346218
6	0	4.526651	0.378437	0.173031
1	0	4.702754	-0.155608	-0.779073
6	0	4.628500	-1.796606	1.456458
1	0	5.610354	-1.525472	1.869534
1	0	4.776363	-2.380769	0.538545
8	0	-2.457375	-2.141103	-0.120485
1	0	-2.182977	-1.501114	-0.800153
6	0	-3.661129	-2.762615	-0.516409
1	0	-4.482553	-2.034988	-0.641193
1	0	-3.550333	-3.312287	-1.468063
6	0	-4.043040	-3.746938	0.567834
1	0	-3.217028	-4.470626	0.695283
1	0	-4.160442	-3.198322	1.520418
8	0	-5.241559	-4.368830	0.157003
1	0	-5.501145	-4.998460	0.839123
1	0	5.509443	0.666757	0.595265
8	0	3.720565	1.511544	-0.034564
6	0	4.289107	2.398542	-0.962685
1	0	3.590518	3.234608	-1.094721
1	0	5.257839	2.794088	-0.605300
1	0	4.456123	1.908346	-1.940204
1	0	4.106160	-2.420713	2.193678

- Endo product:

6	0	0.185012	-0.235102	-1.852033
6	0	0.124520	-1.737585	-1.623364
6	0	-1.078549	-0.891339	-0.075709
6	0	-0.561720	0.293911	-0.881094
6	0	1.013052	-2.037375	-0.355251
6	0	0.137705	-1.448581	0.753335
1	0	0.289337	-2.402154	-2.474639
1	0	0.819344	0.272287	-2.576183
1	0	-0.688227	1.338585	-0.606248
8	0	-1.173552	-1.912399	-1.067936
6	0	2.308124	-1.248913	-0.288502
6	0	0.977969	-0.374328	1.407474
1	0	-0.215961	-2.173715	1.496894
1	0	1.210784	-3.111152	-0.263562
7	0	2.215132	-0.342812	0.764296
8	0	0.650295	0.353105	2.312930
8	0	3.260877	-1.352438	-1.026007
6	0	3.208551	0.693611	1.046033
1	0	2.775492	1.260181	1.882396
6	0	-2.333969	-0.709486	0.730162
1	0	-2.707724	-1.704133	1.040808

1	0	-2.075172	-0.135213	1.641523
8	0	-3.283995	-0.029624	-0.044752
6	0	-4.488008	0.188183	0.647198
1	0	-4.947693	-0.766838	0.964681
1	0	-4.306059	0.789844	1.562650
6	0	-5.435909	0.926282	-0.281679
1	0	-4.951126	1.856533	-0.628069
1	0	-5.626559	0.304256	-1.166651
8	0	-6.682841	1.159528	0.333256
1	0	-6.542921	1.790743	1.050510
6	0	3.333802	1.634760	-0.145034
1	0	3.737215	1.091152	-1.019409
6	0	4.551039	0.092194	1.445267
1	0	5.254456	0.893051	1.713484
1	0	4.974500	-0.492801	0.618242
1	0	4.043877	2.443695	0.117160
8	0	2.061287	2.159866	-0.429140
6	0	2.081198	3.012171	-1.544901
1	0	1.053025	3.352428	-1.722058
1	0	2.725540	3.893670	-1.370444
1	0	2.449420	2.488333	-2.446849
1	0	4.429068	-0.563786	2.317475

Furan + Maleimide (HB: furan (O) + glycol, conformation 1): Exo

- Reactant 1 + glycol:

6	0	3.380934	0.973451	0.383686
6	0	2.050024	0.694068	0.396745
6	0	2.995266	-0.890462	-0.779880
6	0	4.002540	-0.068765	-0.385234
1	0	3.860334	1.822201	0.861306
1	0	5.056649	-0.185138	-0.614489
1	0	2.957793	-1.797369	-1.373281
8	0	1.805210	-0.442215	-0.309243
6	0	0.859111	1.343290	1.002804
1	0	1.182884	2.220986	1.589495
1	0	0.347369	0.634817	1.680986
8	0	-0.025384	1.716144	-0.034496
6	0	-1.276433	2.156598	0.456848
1	0	-1.682282	1.433912	1.185946
1	0	-1.161723	3.139458	0.958421
6	0	-2.225301	2.271424	-0.724084
1	0	-1.745128	2.871871	-1.517834
1	0	-2.411598	1.263551	-1.119064
8	0	-3.473771	2.794660	-0.328094
1	0	-3.337464	3.715950	-0.073306
6	0	-0.828162	-2.135897	-0.181299
1	0	-0.552329	-2.449860	-1.204362
1	0	0.031329	-2.352300	0.477588
6	0	-2.015384	-2.955968	0.274176
1	0	-2.303467	-2.627028	1.289627
1	0	-2.867986	-2.753688	-0.399271
8	0	-1.207257	-0.779290	-0.125866
1	0	-0.494482	-0.237481	-0.496218
8	0	-1.619343	-4.311703	0.243944
1	0	-2.371103	-4.847088	0.521933

- Reactant complex:

6	0	2.226847	-0.156954	2.850767
6	0	2.056483	-0.062895	1.502594
6	0	0.567848	1.270039	2.412100
6	0	1.253347	0.710744	3.445975
1	0	2.958782	-0.779447	3.355683
1	0	1.088242	0.899239	4.501812
6	0	-0.613756	-1.795357	1.226535
1	0	0.109308	-2.604649	1.274085
6	0	-1.446421	-1.323716	2.156078
1	0	-1.612262	-1.663019	3.175562
1	0	-0.247116	1.984871	2.351439
8	0	1.046042	0.812239	1.229818
6	0	-2.188899	-0.146998	1.584665
6	0	-0.753280	-0.938859	-0.001965
7	0	-1.701089	0.033178	0.294031
8	0	-0.144462	-1.020534	-1.045029
8	0	-3.034424	0.529563	2.120669
6	0	2.691747	-0.732976	0.331892
1	0	3.791972	-0.712168	0.435530
1	0	2.422190	-0.177332	-0.584291
8	0	2.235360	-2.066646	0.246238
6	0	-2.195162	1.008536	-0.674058
1	0	-1.612763	0.812104	-1.586023
6	0	-1.949964	2.435525	-0.200486
1	0	-0.872547	2.600223	-0.066392
1	0	-2.306065	3.149621	-0.956685
1	0	-2.477141	2.626420	0.744899
6	0	-5.123938	-0.958172	-1.613676
1	0	-5.137357	-2.020923	-1.884250
1	0	-5.768511	-0.801489	-0.729059
1	0	-5.537207	-0.368715	-2.453222
6	0	2.496799	-2.641050	-1.015962
1	0	2.159862	-1.970279	-1.824948
1	0	3.584881	-2.823941	-1.140016
6	0	1.732531	-3.950707	-1.092630
1	0	1.955314	-4.552935	-0.191864
1	0	0.656776	-3.725650	-1.097258
8	0	2.008027	-4.644921	-2.287524
1	0	2.936302	-4.910042	-2.264559
6	0	1.769354	2.660862	-1.012460
1	0	1.243023	3.188624	-0.195462
1	0	2.681793	2.213365	-0.576562
6	0	2.173544	3.671802	-2.063352
1	0	2.711582	3.146668	-2.873372
1	0	1.258728	4.114202	-2.498646
8	0	0.953871	1.703303	-1.649258
1	0	0.720165	1.007639	-1.018835
8	0	2.980639	4.635743	-1.420172
1	0	3.246662	5.288280	-2.077819
6	0	-3.664401	0.740107	-0.971218
1	0	-3.991281	1.412442	-1.788819
1	0	-4.279504	0.958838	-0.077580
8	0	-3.791586	-0.606719	-1.344786

- Transition state:

6	0	-1.369096	0.346408	3.042365
6	0	-1.261355	0.006603	1.669053
6	0	0.095449	-1.302631	2.722165
6	0	-0.518286	-0.499287	3.716474
1	0	-1.928652	1.196945	3.420617
1	0	-0.239091	-0.477334	4.766137
6	0	0.623050	1.021664	1.354722
1	0	0.313505	2.015768	1.661153
6	0	1.479718	0.147473	2.037465
1	0	1.999832	0.350264	2.969745
1	0	0.668076	-2.220765	2.832507
8	0	-0.647098	-1.207213	1.578915
6	0	2.183347	-0.683990	1.010416
6	0	0.782155	0.748977	-0.104947
7	0	1.598803	-0.393368	-0.226909
8	0	0.300367	1.348286	-1.036710
8	0	3.082207	-1.475314	1.186671
6	0	-2.148730	0.437818	0.540149
1	0	-3.165466	0.017315	0.673563
1	0	-1.739075	0.064097	-0.412365
8	0	-2.173605	1.841346	0.582430
6	0	2.231653	-0.728974	-1.505235
1	0	1.619192	-0.203473	-2.251010
6	0	2.244809	-2.227058	-1.782626
1	0	1.220763	-2.606858	-1.881143
1	0	2.776618	-2.418706	-2.726456
1	0	2.764929	-2.759083	-0.974597
6	0	4.877648	1.747215	-0.979258
1	0	4.755902	2.760627	-0.577799
1	0	5.546810	1.170894	-0.313945
1	0	5.349512	1.811211	-1.977225
6	0	-2.619879	2.422625	-0.624899
1	0	-2.152115	1.921775	-1.488737
1	0	-3.722482	2.332453	-0.713852
6	0	-2.209506	3.884225	-0.604397
1	0	-2.541532	4.340594	0.346466
1	0	-1.112997	3.935151	-0.642757
8	0	-2.695099	4.574545	-1.733528
1	0	-3.658305	4.598045	-1.669702
6	0	-2.016555	-2.770346	-0.806389
1	0	-1.717515	-3.823566	-0.660527
1	0	-2.460249	-2.415815	0.141167
6	0	-3.062780	-2.703539	-1.898055
1	0	-3.376003	-1.650431	-2.029179
1	0	-2.607123	-3.038024	-2.847597
8	0	-0.929506	-1.964572	-1.212846
1	0	-0.350553	-1.823924	-0.448682
8	0	-4.132284	-3.531726	-1.493949
1	0	-4.790302	-3.534796	-2.198420
8	0	3.602887	1.163861	-1.049016
6	0	3.647140	-0.152668	-1.536001
1	0	4.019992	-0.178086	-2.578787
1	0	4.319060	-0.771486	-0.911967

- Exo product + glycol:

6	0	-1.193565	-0.637209	-3.120039
6	0	-0.954319	-0.192788	-1.683286
6	0	0.595607	0.735692	-2.843240
6	0	-0.236824	-0.051974	-3.843781
1	0	-1.951359	-1.357920	-3.420367
1	0	-0.010122	-0.168368	-4.901581
6	0	0.308420	-0.994011	-1.207296
1	0	0.180612	-2.072048	-1.340031
6	0	1.415470	-0.312405	-2.026060
1	0	2.026150	-0.962644	-2.661685
1	0	1.162831	1.603730	-3.190492
8	0	-0.389896	1.112683	-1.873109
6	0	2.299284	0.372124	-0.993794
6	0	0.656545	-0.694349	0.242889
7	0	1.764713	0.150785	0.264757
8	0	0.073223	-1.092800	1.220187
8	0	3.294310	1.018111	-1.227537
6	0	-2.121714	-0.195753	-0.726666
1	0	-2.963066	0.383827	-1.150902
1	0	-1.808164	0.265710	0.223936
8	0	-2.466557	-1.548568	-0.551021
6	0	2.417482	0.543791	1.515665
1	0	1.754016	0.145259	2.295390
6	0	2.549575	2.055874	1.647911
1	0	1.558238	2.521304	1.585076
1	0	2.997686	2.299182	2.622317
1	0	3.193364	2.456689	0.853397
6	0	4.771466	-2.253356	1.434684
1	0	4.537378	-3.302495	1.216323
1	0	5.505253	-1.883890	0.694543
1	0	5.230162	-2.190292	2.438939
6	0	-3.104485	-1.797242	0.682856
1	0	-2.483205	-1.426859	1.515920
1	0	-4.089235	-1.285818	0.719128
6	0	-3.294641	-3.298035	0.807896
1	0	-3.815850	-3.674600	-0.091251
1	0	-2.306853	-3.777162	0.847896
8	0	-3.962397	-3.637504	2.001992
1	0	-4.860416	-3.287223	1.944467
6	0	-1.715276	3.051990	0.501528
1	0	-1.466027	4.122271	0.390528
1	0	-2.279449	2.746475	-0.397416
6	0	-2.592302	2.880581	1.724015
1	0	-2.891431	1.818200	1.810253
1	0	-1.998699	3.133521	2.621446
8	0	-0.557673	2.261052	0.681230
1	0	-0.228824	2.024274	-0.201234
8	0	-3.705194	3.733859	1.559062
1	0	-4.248818	3.672154	2.352713
6	0	3.766012	-0.159634	1.618922
1	0	4.173547	0.005579	2.635626
1	0	4.475541	0.271172	0.887664
8	0	3.570852	-1.528745	1.370803

- Exo product:

6	0	1.885454	-2.618056	-0.800997
6	0	1.364397	-1.521836	0.121376
6	0	-0.249708	-2.902129	-0.085107
6	0	0.880128	-3.487830	-0.920818
1	0	2.851052	-2.595848	-1.302216
1	0	0.812115	-4.373240	-1.549835
6	0	0.365784	-0.685729	-0.762011
1	0	0.828590	-0.334133	-1.688581
6	0	-0.800941	-1.678720	-0.888705
1	0	-1.117631	-1.937102	-1.905023
1	0	-1.015523	-3.571394	0.316907
8	0	0.456293	-2.231831	0.957356
6	0	-1.949565	-1.029978	-0.134798
6	0	-0.207406	0.489201	0.011458
7	0	-1.523129	0.194966	0.351933
8	0	0.369482	1.503442	0.325265
8	0	-3.048385	-1.503031	0.041356
6	0	2.370060	-0.719515	0.907522
1	0	3.033057	-1.396745	1.476376
1	0	1.840774	-0.064636	1.619718
8	0	3.087883	0.033569	-0.038624
6	0	-2.368834	1.126542	1.095650
1	0	-1.736998	2.015982	1.226196
6	0	-2.773049	0.551839	2.448259
1	0	-1.880695	0.308081	3.040047
1	0	-3.369294	1.287757	3.006378
1	0	-3.368617	-0.361224	2.313862
6	0	-4.166382	2.304935	-1.857175
1	0	-3.725002	2.615914	-2.811896
1	0	-4.848603	1.452989	-2.033521
1	0	-4.757699	3.143985	-1.445524
6	0	3.663886	1.202109	0.500969
1	0	2.888602	1.826559	0.976764
1	0	4.427392	0.942151	1.263657
6	0	4.310651	1.964711	-0.641147
1	0	5.008352	1.294864	-1.176315
1	0	3.528796	2.268931	-1.350399
8	0	4.934134	3.145162	-0.188592
1	0	5.672742	2.887362	0.377506
6	0	-3.577181	1.517444	0.253606
1	0	-4.121183	2.329327	0.775807
1	0	-4.261133	0.656129	0.143280
8	0	-3.113276	1.947888	-0.999913

Furan + Maleimide (HB: furan (O) + glycol, conformation 2): Exo

- Reactant 1 + glycol:

6	0	-1.184973	1.362254	-1.261104
6	0	-0.916703	1.271244	0.067508
6	0	-3.090366	1.482527	-0.098289
6	0	-2.612254	1.500078	-1.368355
1	0	-0.447258	1.327440	-2.055046
1	0	-3.201308	1.596414	-2.274697
1	0	-4.082182	1.558985	0.332932
8	0	-2.065393	1.342833	0.791485
6	0	0.352918	1.107105	0.830855

1	0	0.228334	0.288783	1.564226
1	0	0.596571	2.036123	1.382900
8	0	1.364080	0.806195	-0.099086
6	0	2.612106	0.605940	0.520116
1	0	2.939419	1.513107	1.061829
1	0	2.545790	-0.220106	1.258122
6	0	3.624149	0.265039	-0.559469
1	0	3.281964	-0.629137	-1.111437
1	0	3.676508	1.095498	-1.276768
8	0	4.916359	0.106802	-0.020709
1	0	4.908573	-0.680638	0.538338
6	0	-1.740403	-1.874191	0.478821
1	0	-2.401251	-2.756564	0.538235
1	0	-2.215319	-1.140340	-0.195124
6	0	-0.415046	-2.303172	-0.119277
1	0	0.217290	-1.410867	-0.283860
1	0	0.098029	-2.964631	0.604820
8	0	-1.506261	-1.336947	1.769960
1	0	-2.026163	-0.527601	1.845886
8	0	-0.711029	-2.970448	-1.329967
1	0	0.122385	-3.174895	-1.768651

- Reactant complex:

6	0	1.415439	0.970769	-2.489455
6	0	0.968198	0.734273	-1.224427
6	0	1.585260	-1.207443	-2.034335
6	0	1.817335	-0.298352	-3.020320
1	0	1.439038	1.938198	-2.980623
1	0	2.229855	-0.508154	-4.002048
6	0	-2.015341	-0.208149	-1.727479
1	0	-2.311474	0.757224	-2.127502
6	0	-1.555239	-1.292801	-2.352477
1	0	-1.382080	-1.466151	-3.411371
1	0	1.717054	-2.281471	-1.955860
8	0	1.069175	-0.592423	-0.939357
6	0	-1.240152	-2.329932	-1.316306
6	0	-2.064049	-0.487582	-0.246182
7	0	-1.579383	-1.780850	-0.078065
8	0	-2.461049	0.253804	0.623259
8	0	-0.774876	-3.433569	-1.470866
6	0	0.374766	1.614442	-0.177677
1	0	1.119665	2.348575	0.180205
1	0	0.073908	0.988717	0.677066
8	0	-0.745705	2.278135	-0.730512
6	0	-1.363144	-2.544655	1.149735
1	0	-0.427893	-3.099488	0.978464
6	0	-2.502453	-3.535969	1.369417
1	0	-2.596233	-4.194555	0.496239
1	0	-2.306408	-4.159972	2.253070
1	0	-3.453095	-3.002364	1.518218
6	0	-0.022984	0.191250	3.193822
1	0	0.873156	0.793861	3.005717
1	0	-0.925494	0.827930	3.182730
1	0	0.063034	-0.293035	4.183059
6	0	-1.382804	3.097993	0.222203
1	0	-1.525398	2.555682	1.173444

1	0	-0.767704	4.000094	0.425787
6	0	-2.736121	3.499092	-0.335938
1	0	-2.601412	3.895253	-1.359772
1	0	-3.367505	2.601754	-0.393635
8	0	-3.399381	4.409694	0.512321
1	0	-2.884388	5.226541	0.522065
6	0	3.607019	-0.068700	0.656699
1	0	3.906331	-1.127699	0.750912
1	0	3.447125	0.144232	-0.416656
6	0	4.737063	0.802773	1.160989
1	0	4.425795	1.861471	1.085840
1	0	4.913372	0.578622	2.228956
8	0	2.467794	0.231431	1.434331
1	0	1.775193	-0.424848	1.256754
8	0	5.866470	0.521282	0.361828
1	0	6.594566	1.072688	0.669750
8	0	-0.093541	-0.777853	2.164980
6	0	-1.177513	-1.653898	2.365000
1	0	-0.979834	-2.310701	3.233737
1	0	-2.096269	-1.079122	2.564424

- Transition state:

6	0	0.901967	0.925043	-2.640621
6	0	0.545795	0.642007	-1.295717
6	0	1.413641	-1.170191	-2.078542
6	0	1.480652	-0.221343	-3.133341
1	0	0.640619	1.840395	-3.163381
1	0	1.788646	-0.436197	-4.152724
6	0	-1.238019	-0.520800	-1.834647
1	0	-1.756069	0.200029	-2.459679
6	0	-0.590242	-1.694793	-2.252096
1	0	-0.592247	-2.106689	-3.258060
1	0	1.938890	-2.116603	-1.969318
8	0	1.178828	-0.495018	-0.912804
6	0	-0.603309	-2.641222	-1.089299
6	0	-1.727112	-0.741516	-0.444426
7	0	-1.209148	-1.970147	-0.028101
8	0	-2.432861	-0.020746	0.230356
8	0	-0.155158	-3.762796	-1.022598
6	0	0.110902	1.600940	-0.232093
1	0	0.955945	2.244719	0.074103
1	0	-0.219622	1.030170	0.650474
8	0	-0.939875	2.345253	-0.794664
6	0	-1.308317	-2.575098	1.294808
1	0	-0.434461	-3.239932	1.362397
6	0	-2.585239	-3.402358	1.416543
1	0	-2.596560	-4.184040	0.645660
1	0	-2.642026	-3.886517	2.402323
1	0	-3.468018	-2.758978	1.287010
6	0	-0.028407	0.256052	3.263830
1	0	0.887676	0.834297	3.101542
1	0	-0.911411	0.918669	3.209774
1	0	-0.000077	-0.212087	4.264226
6	0	-1.708269	3.026651	0.171310
1	0	-1.917151	2.368327	1.031921
1	0	-1.168432	3.925134	0.536263

6	0	-3.018528	3.424490	-0.483710
1	0	-2.805749	3.959034	-1.427836
1	0	-3.572482	2.508167	-0.728079
8	0	-3.827944	4.176795	0.392422
1	0	-3.381991	5.018363	0.551507
6	0	3.534177	0.360260	0.654514
1	0	3.869658	-0.690366	0.605134
1	0	3.376567	0.709227	-0.382697
6	0	4.626411	1.194466	1.288902
1	0	4.268078	2.237191	1.376151
1	0	4.817799	0.818166	2.310260
8	0	2.373853	0.510839	1.443607
1	0	1.751641	-0.206780	1.244887
8	0	5.762370	1.088831	0.457080
1	0	6.472076	1.606925	0.853320
8	0	-0.085119	-0.730348	2.251166
6	0	-1.227164	-1.543193	2.409200
1	0	-1.169462	-2.091431	3.369291
1	0	-2.134188	-0.917011	2.418030

- Exo product + glycol:

6	0	-1.276882	0.131873	2.711945
6	0	-0.573309	0.127649	1.361704
6	0	-0.605472	-1.919162	1.998672
6	0	-1.296038	-1.143274	3.109070
1	0	-1.588190	1.029825	3.241906
1	0	-1.640232	-1.560408	4.053172
6	0	0.948992	-0.107902	1.712437
1	0	1.300662	0.593106	2.473665
6	0	0.921231	-1.601364	2.076939
1	0	1.386150	-1.887552	3.026587
1	0	-0.846691	-2.976223	1.857323
8	0	-0.940724	-1.150310	0.841559
6	0	1.605035	-2.286868	0.902284
6	0	1.812910	-0.020450	0.468440
7	0	2.061190	-1.310887	0.024171
8	0	2.211137	0.985508	-0.073098
8	0	1.714053	-3.474909	0.725364
6	0	-0.833576	1.249582	0.386034
1	0	-1.917754	1.383053	0.222008
1	0	-0.366192	0.994648	-0.580606
8	0	-0.242278	2.388312	0.962916
6	0	2.642573	-1.651228	-1.272473
1	0	2.332262	-2.692184	-1.439697
6	0	4.163673	-1.558490	-1.234890
1	0	4.570939	-2.257996	-0.492836
1	0	4.585250	-1.813517	-2.217993
1	0	4.473525	-0.536326	-0.973656
6	0	0.113297	0.140155	-3.243967
1	0	-0.967996	0.171731	-3.073836
1	0	0.536162	1.160935	-3.192973
1	0	0.323572	-0.275227	-4.246505
6	0	0.113364	3.375671	0.022323
1	0	0.649174	2.921764	-0.829073
1	0	-0.790555	3.890719	-0.364076
6	0	1.026784	4.365759	0.721727

1	0	0.537418	4.722638	1.646552
1	0	1.949534	3.842583	1.007056
8	0	1.399316	5.422197	-0.134755
1	0	0.603447	5.932005	-0.332639
6	0	-3.482819	-0.955262	-0.804767
1	0	-3.692537	-2.040072	-0.791369
1	0	-3.424188	-0.615413	0.244958
6	0	-4.627285	-0.245836	-1.495367
1	0	-4.412589	0.839450	-1.513842
1	0	-4.683402	-0.594334	-2.542914
8	0	-2.306844	-0.652072	-1.521928
1	0	-1.558873	-1.060386	-1.057220
8	0	-5.801396	-0.543935	-0.769420
1	0	-6.543097	-0.120116	-1.215861
8	0	0.668483	-0.678687	-2.234671
6	0	2.064194	-0.773604	-2.378446
1	0	2.323402	-1.240395	-3.348877
1	0	2.518458	0.231568	-2.346170

- Exo product:

6	0	-0.641384	3.148599	0.103232
6	0	-0.405629	1.669848	0.390867
6	0	1.539700	2.531501	0.253823
6	0	0.577280	3.688367	0.019539
1	0	-1.614837	3.596374	-0.087365
1	0	0.857434	4.704168	-0.252472
6	0	0.086019	1.078917	-0.988606
1	0	-0.590719	1.341454	-1.806515
6	0	1.512125	1.650390	-1.037348
1	0	1.792655	2.194330	-1.946281
1	0	2.540236	2.740053	0.642532
8	0	0.801173	1.695156	1.139173
6	0	2.413368	0.441379	-0.835654
6	0	0.267425	-0.425237	-0.917916
7	0	1.621578	-0.704684	-0.830518
8	0	-0.606132	-1.265171	-0.914731
8	0	3.609418	0.451977	-0.687711
6	0	-1.480936	0.871962	1.082424
1	0	-1.790213	1.376314	2.016202
1	0	-1.068514	-0.120580	1.333960
8	0	-2.542827	0.768404	0.167798
6	0	2.150769	-2.014927	-0.457756
1	0	3.208689	-1.819868	-0.234366
6	0	2.020101	-3.021198	-1.593810
1	0	2.588989	-2.687742	-2.471731
1	0	2.414699	-3.997916	-1.277800
1	0	0.964461	-3.140435	-1.874067
6	0	0.759314	-1.774235	2.918852
1	0	0.683686	-0.859105	3.519115
1	0	-0.252766	-2.195355	2.766998
1	0	1.367512	-2.518708	3.465097
6	0	-3.368417	-0.353297	0.381633
1	0	-2.756564	-1.259078	0.533237
1	0	-4.007703	-0.203859	1.276673
6	0	-4.229001	-0.529929	-0.856347
1	0	-4.739831	0.423595	-1.083939

1	0	-3.571511	-0.771824	-1.702368
8	0	-5.129929	-1.605133	-0.712723
1	0	-5.763636	-1.365032	-0.024867
8	0	1.352792	-1.420155	1.697222
6	0	1.461475	-2.507280	0.815918
1	0	2.075738	-3.317651	1.255288
1	0	0.461935	-2.916127	0.580978

Furan + Maleimide (HB: maleimide C=O + glycol, conformation 1): Endo

- Reactant 1 + glycol:

6	0	-0.198337	-2.106061	-0.570735
6	0	0.611433	-1.878251	-1.605608
6	0	0.319442	-1.321295	0.605168
6	0	1.675362	-0.906199	-1.175933
1	0	0.563281	-2.261745	-2.621905
1	0	-1.103144	-2.705682	-0.501028
7	0	1.484914	-0.691389	0.192177
8	0	2.519470	-0.383305	-1.859672
8	0	-0.160277	-1.241786	1.714758
6	0	2.069974	0.417084	0.945300
1	0	1.633047	0.319886	1.949062
6	0	1.590167	1.736263	0.348116
1	0	1.884952	2.564152	1.021653
1	0	2.066191	1.906610	-0.636903
6	0	3.587857	0.324800	1.013471
1	0	3.983177	1.149428	1.623448
1	0	3.891228	-0.623080	1.477167
1	0	4.025931	0.383087	0.008678
8	0	0.192886	1.666205	0.218093
6	0	-0.366377	2.873023	-0.248912
1	0	0.062941	3.156190	-1.228003
1	0	-1.446678	2.710664	-0.349764
1	0	-0.181366	3.698042	0.462820
6	0	-3.116560	-0.224134	0.750137
1	0	-4.192944	-0.065278	0.925768
1	0	-2.560098	0.414562	1.456586
6	0	-2.803816	0.212636	-0.671284
1	0	-3.245337	-0.525562	-1.365362
1	0	-1.708121	0.231363	-0.825636
8	0	-2.810116	-1.592622	0.929470
1	0	-2.010083	-1.634643	1.475919
8	0	-3.368965	1.501984	-0.850211
1	0	-3.256226	1.750735	-1.775562

- Reactant complex:

6	0	-0.895696	-2.181136	1.835211
6	0	-0.868280	-2.952889	0.712286
6	0	1.201092	-2.359134	1.074086
6	0	0.463006	-1.793586	2.072126
6	0	-0.873083	-0.340175	-0.861168
6	0	0.435833	-0.216816	-1.089508
1	0	-1.653617	-3.431935	0.137996
1	0	-1.781173	-1.915149	2.403961
1	0	0.852036	-1.171280	2.872385

8	0	0.397515	-3.080754	0.251287
6	0	-1.239195	0.566915	0.275767
6	0	0.994058	0.782506	-0.116319
1	0	1.070001	-0.739986	-1.798013
1	0	-1.615437	-0.982015	-1.330091
7	0	-0.076080	1.194002	0.684604
8	0	2.128982	1.185660	-0.014083
8	0	-2.334811	0.728285	0.777934
6	0	-0.045155	2.309492	1.623284
1	0	-1.096773	2.451204	1.910781
6	0	2.654624	-2.314540	0.765802
1	0	3.148668	-1.746396	1.574065
1	0	3.083850	-3.334161	0.751019
8	0	2.862291	-1.687254	-0.481046
6	0	4.186020	-1.239442	-0.652577
1	0	4.470437	-0.539754	0.154558
1	0	4.892819	-2.095693	-0.631929
6	0	4.267135	-0.525596	-1.990588
1	0	3.878068	-1.194228	-2.780901
1	0	3.626966	0.365671	-1.945431
8	0	5.577919	-0.080285	-2.258567
1	0	6.130588	-0.860452	-2.393706
6	0	0.431856	3.572584	0.913036
1	0	0.279616	4.438263	1.587560
1	0	1.511063	3.496923	0.684756
6	0	0.803574	1.992564	2.848255
1	0	0.852333	2.867403	3.512736
1	0	0.364921	1.158978	3.412572
1	0	1.825035	1.724028	2.543703
8	0	-0.315670	3.713974	-0.266338
6	0	0.108694	4.811810	-1.033444
1	0	1.169463	4.709727	-1.326857
1	0	-0.512688	4.843369	-1.936587
1	0	-0.010985	5.762602	-0.481693
6	0	-4.871259	-1.469058	-0.534669
1	0	-5.224097	-2.372641	-1.051928
1	0	-5.570823	-1.267873	0.296261
6	0	-4.904977	-0.291810	-1.493842
1	0	-4.247927	-0.512404	-2.357094
1	0	-4.497426	0.599025	-0.978705
8	0	-3.560722	-1.725231	-0.076457
1	0	-3.253076	-0.917440	0.373565
8	0	-6.249858	-0.112370	-1.885352
1	0	-6.293948	0.659865	-2.460675

- Transition state:

6	0	-0.177746	-1.668489	-1.745893
6	0	-1.850810	-0.359659	-1.410630
6	0	-1.055179	0.269668	-2.412039
6	0	0.409684	-0.727598	0.069708
6	0	-0.711846	0.090046	0.279423
1	0	0.356767	-2.614785	-1.705471
1	0	0.910737	-0.384507	-3.209787
1	0	-1.207075	1.277040	-2.791861
8	0	-1.473783	-1.670315	-1.333225
6	0	1.545252	0.164640	-0.289294

6	0	-0.291762	1.501155	0.049213
1	0	-1.540856	-0.123455	0.949380
1	0	0.614694	-1.684237	0.542908
7	0	1.044877	1.456596	-0.364949
8	0	-0.950705	2.513688	0.143408
8	0	2.703335	-0.134820	-0.530869
6	0	1.862553	2.619258	-0.688912
1	0	2.864238	2.205331	-0.872531
6	0	-3.269785	-0.030808	-1.073350
1	0	-3.355674	1.071021	-1.022194
1	0	-3.934467	-0.394771	-1.881113
8	0	-3.619780	-0.628828	0.146676
6	0	-4.967676	-0.404813	0.488210
1	0	-5.191192	0.676459	0.547523
1	0	-5.636499	-0.844080	-0.280438
6	0	-5.225379	-1.059407	1.834460
1	0	-4.933145	-2.123010	1.779425
1	0	-4.593675	-0.581040	2.595504
8	0	-6.562144	-0.880639	2.242338
1	0	-7.121215	-1.414970	1.664201
6	0	1.949672	3.549844	0.515031
1	0	2.660203	4.367796	0.281549
1	0	0.959739	3.997285	0.720137
6	0	1.341552	3.337142	-1.928831
1	0	1.982588	4.197580	-2.168600
1	0	1.343507	2.656918	-2.791937
1	0	0.316882	3.696340	-1.758471
8	0	2.393324	2.796936	1.613180
6	0	2.431868	3.556735	2.793013
1	0	1.431912	3.951728	3.050398
1	0	2.776206	2.897972	3.599528
1	0	3.130318	4.409821	2.704056
6	0	3.414513	-3.614848	-0.087941
1	0	3.116717	-4.672724	-0.055415
1	0	4.404822	-3.564102	-0.575300
6	0	3.544716	-3.082365	1.328666
1	0	2.569205	-3.183319	1.842174
1	0	3.791278	-2.004342	1.281681
8	0	2.428887	-2.913422	-0.812588
1	0	2.684014	-1.969926	-0.808641
8	0	4.557143	-3.833533	1.965578
1	0	4.687415	-3.471387	2.849398

- Endo product + glycol:

6	0	0.045202	-0.687627	-2.732066
6	0	0.030078	-1.652155	-1.555242
6	0	-1.676298	-0.394886	-1.269631
6	0	-1.019184	0.100596	-2.555355
6	0	0.467349	-0.831095	-0.280196
6	0	-0.758777	0.060764	-0.074296
1	0	0.533110	-2.615589	-1.653460
1	0	0.830507	-0.612829	-3.482318
1	0	-1.317113	0.982793	-3.120587
8	0	-1.362703	-1.784204	-1.294462
6	0	1.615696	0.135871	-0.476775
6	0	-0.251100	1.477728	-0.190124

1	0	-1.301559	-0.093659	0.864038
1	0	0.686354	-1.519830	0.543091
7	0	1.125434	1.426570	-0.412378
8	0	-0.900268	2.496721	-0.146572
8	0	2.783499	-0.134735	-0.687208
6	0	1.977898	2.602984	-0.581976
1	0	2.997109	2.194743	-0.619606
6	0	-3.146122	-0.126190	-1.094052
1	0	-3.318020	0.962534	-1.196184
1	0	-3.702303	-0.650569	-1.893502
8	0	-3.545454	-0.577935	0.173279
6	0	-4.918651	-0.387036	0.409692
1	0	-5.195367	0.680838	0.326826
1	0	-5.519717	-0.947013	-0.336319
6	0	-5.228772	-0.892016	1.808102
1	0	-4.894277	-1.941109	1.893988
1	0	-4.659118	-0.301683	2.538949
8	0	-6.592280	-0.726958	2.124197
1	0	-7.098117	-1.334908	1.570163
6	0	1.869478	3.507874	0.639109
1	0	2.634013	4.305078	0.551602
1	0	0.873476	3.984520	0.676942
6	0	1.648988	3.337326	-1.876618
1	0	2.320906	4.198064	-2.003758
1	0	1.780417	2.667383	-2.737350
1	0	0.611091	3.697379	-1.859337
8	0	2.089207	2.722489	1.781982
6	0	1.961427	3.468792	2.965356
1	0	0.950332	3.905956	3.057460
1	0	2.136397	2.787183	3.806665
1	0	2.701403	4.289478	3.008733
6	0	3.514841	-3.518268	0.150654
1	0	3.316340	-4.599580	0.157746
1	0	4.593991	-3.381334	-0.042588
6	0	3.192996	-2.940340	1.518419
1	0	2.127471	-3.136310	1.747778
1	0	3.338414	-1.842725	1.483309
8	0	2.715186	-2.943016	-0.856818
1	0	2.933901	-1.993836	-0.894862
8	0	4.054341	-3.560185	2.449395
1	0	3.879799	-3.182302	3.318875

- Endo product:

6	0	-0.257992	-2.935116	0.390716
6	0	0.253293	-2.581122	-0.998791
6	0	1.268146	-1.244605	0.322373
6	0	0.375026	-2.099030	1.218115
6	0	-0.403365	-1.208014	-1.400294
6	0	0.327838	-0.252450	-0.454879
1	0	0.227659	-3.353907	-1.770497
1	0	-1.054956	-3.641745	0.614763
1	0	0.220312	-1.950044	2.285934
8	0	1.583929	-2.155202	-0.730174
6	0	-1.871090	-1.066595	-1.045716
6	0	-0.752388	0.371089	0.400064
1	0	0.943911	0.510973	-0.941186

1	0	-0.246697	-1.006318	-2.465577
7	0	-1.977052	-0.133504	-0.013628
8	0	-0.593933	1.141840	1.320882
8	0	-2.801878	-1.666186	-1.523863
6	0	-3.261337	0.217282	0.587831
1	0	-4.002988	-0.274230	-0.057147
6	0	2.486654	-0.625328	0.950352
1	0	2.160107	-0.018878	1.816835
1	0	3.153366	-1.431259	1.310461
8	0	3.136528	0.174329	-0.002300
6	0	4.293563	0.791008	0.506526
1	0	4.058538	1.422418	1.383899
1	0	5.027344	0.025048	0.832929
6	0	4.894081	1.645496	-0.596170
1	0	5.067327	1.014699	-1.486104
1	0	4.175167	2.426755	-0.878725
8	0	6.061341	2.303429	-0.158858
1	0	6.744099	1.633580	-0.026526
6	0	-3.484650	1.722470	0.515258
1	0	-4.519425	1.939003	0.847800
1	0	-2.785627	2.246677	1.191600
6	0	-3.362233	-0.311437	2.014297
1	0	-4.346077	-0.068885	2.440902
1	0	-3.242530	-1.403635	2.022918
1	0	-2.581982	0.138207	2.644087
8	0	-3.288779	2.128013	-0.814406
6	0	-3.429062	3.516786	-0.966909
1	0	-2.698783	4.065671	-0.344419
1	0	-3.250672	3.753343	-2.022941
1	0	-4.445304	3.854670	-0.690498

Furan + Maleimide (HB: maleimide C=O + glycol, conformation 2): Endo

- Reactant 1 + glycol:

6	0	0.502714	-0.998830	-1.296606
6	0	-0.363320	-2.001993	-1.447112
6	0	0.019350	-0.135541	-0.161993
6	0	-1.452309	-1.853980	-0.421091
1	0	-0.358066	-2.821098	-2.162129
1	0	1.411590	-0.746444	-1.841238
7	0	-1.109672	-0.735020	0.354888
8	0	-2.426550	-2.538642	-0.260812
8	0	0.513007	0.902341	0.239528
6	0	-2.037227	-0.111678	1.295056
1	0	-2.883665	-0.811880	1.337647
6	0	-2.536661	1.212202	0.724419
1	0	-3.377988	1.574375	1.347361
1	0	-1.731333	1.970171	0.758236
6	0	-1.413795	0.059470	2.674017
1	0	-2.142211	0.511327	3.362401
1	0	-1.111607	-0.914117	3.081700
1	0	-0.531067	0.711055	2.617068
8	0	-2.946258	0.983788	-0.598080
6	0	-3.388605	2.159419	-1.228398
1	0	-2.590814	2.923971	-1.256237
1	0	-3.671089	1.896757	-2.254872

1	0	-4.266723	2.589227	-0.712357
6	0	3.976221	0.901267	-0.649030
1	0	4.793518	0.570695	-1.305772
1	0	4.280148	1.866005	-0.204427
6	0	3.796719	-0.113370	0.467766
1	0	3.515156	-1.088875	0.025320
1	0	2.965239	0.212509	1.121905
8	0	2.818062	1.023098	-1.443752
1	0	2.086437	1.285995	-0.858422
8	0	5.022311	-0.181699	1.165349
1	0	4.917081	-0.797411	1.899564

- Reactant complex:

6	0	0.756984	0.544989	2.199952
6	0	0.515808	1.862201	1.947208
6	0	-1.433696	0.880855	1.915664
6	0	-0.522580	-0.098137	2.182544
6	0	0.518518	0.907049	-0.952516
6	0	-0.802696	0.824489	-1.118281
1	0	1.174015	2.716014	1.830153
1	0	1.732892	0.095241	2.353369
1	0	-0.720307	-1.158229	2.308830
8	0	-0.809281	2.081997	1.784797
6	0	1.062747	-0.475996	-0.729092
6	0	-1.193843	-0.619785	-1.013995
1	0	-1.539339	1.609386	-1.257421
1	0	1.174799	1.774250	-0.927531
7	0	-0.008578	-1.343407	-0.795521
8	0	-2.284376	-1.127356	-1.106977
8	0	2.227929	-0.775790	-0.533460
6	0	-0.028130	-2.799303	-0.657775
1	0	-0.990907	-3.021244	-0.173296
6	0	-2.908867	0.845514	1.738600
1	0	-3.242092	-0.189318	1.932648
1	0	-3.409800	1.511317	2.466620
8	0	-3.242932	1.236117	0.423238
6	0	-4.541791	0.842214	0.050608
1	0	-4.670832	-0.248557	0.173164
1	0	-5.300574	1.353200	0.680305
6	0	-4.737770	1.212204	-1.409077
1	0	-4.492047	2.281699	-1.546681
1	0	-4.039008	0.617193	-2.012703
8	0	-6.037995	0.896280	-1.853529
1	0	-6.654870	1.476073	-1.389054
6	0	1.092557	-3.299019	0.240834
1	0	0.955048	-4.388576	0.382929
1	0	2.070133	-3.131791	-0.240232
6	0	0.014913	-3.470630	-2.026635
1	0	-0.050428	-4.563111	-1.921485
1	0	-0.831582	-3.132080	-2.638383
1	0	0.952325	-3.223134	-2.546892
8	0	1.027198	-2.625605	1.471669
6	0	2.217788	-2.754809	2.210186
1	0	3.062365	-2.289816	1.670644
1	0	2.067466	-2.240269	3.168011
1	0	2.458604	-3.814988	2.409658

6	0	4.488548	1.983014	-0.064417
1	0	4.759452	3.017894	0.188789
1	0	5.252178	1.321652	0.383052
6	0	4.518928	1.807621	-1.572887
1	0	3.799003	2.512777	-2.030687
1	0	4.187091	0.779971	-1.817398
8	0	3.200437	1.739619	0.457865
1	0	2.972603	0.818453	0.236807
8	0	5.843637	2.053850	-1.994822
1	0	5.882402	1.932131	-2.950330

- Transition state:

6	0	-0.387120	-0.699285	-1.958243
6	0	-0.040430	0.672432	-1.840850
6	0	1.654436	-0.421895	-1.098285
6	0	0.695323	-1.402577	-1.489313
6	0	-0.321850	0.849772	0.267978
6	0	0.825278	0.163574	0.703667
1	0	-0.556092	1.549397	-2.225281
1	0	-1.374365	-1.082303	-2.201638
1	0	0.737965	-2.463833	-1.258768
8	0	1.305168	0.768195	-1.675846
6	0	-1.495106	-0.006868	0.580608
6	0	0.380690	-1.129627	1.295374
1	0	1.748558	0.623590	1.047115
1	0	-0.449545	1.927233	0.206558
7	0	-1.015370	-1.185462	1.124692
8	0	1.039954	-2.007166	1.799323
8	0	-2.682461	0.224539	0.384324
6	0	-1.816848	-2.352943	1.475470
1	0	-1.079014	-3.095354	1.812633
6	0	3.108269	-0.661726	-0.845557
1	0	3.193981	-1.579027	-0.233132
1	0	3.625786	-0.836621	-1.808962
8	0	3.668064	0.446250	-0.190493
6	0	5.051247	0.297913	0.028102
1	0	5.267329	-0.606443	0.626520
1	0	5.583992	0.194689	-0.939790
6	0	5.545644	1.532543	0.761807
1	0	5.263626	2.431171	0.184988
1	0	5.047283	1.595984	1.738885
8	0	6.929899	1.458015	1.014216
1	0	7.388746	1.536798	0.168273
6	0	-2.527922	-2.916214	0.252998
1	0	-3.142680	-3.780582	0.571218
1	0	-3.203546	-2.154430	-0.179388
6	0	-2.798607	-2.034733	2.599504
1	0	-3.335663	-2.944285	2.903674
1	0	-2.257469	-1.645597	3.472179
1	0	-3.527486	-1.281245	2.271482
8	0	-1.566349	-3.322699	-0.692465
6	0	-2.160645	-3.889026	-1.833196
1	0	-2.851404	-3.178040	-2.324387
1	0	-1.356360	-4.148995	-2.533112
1	0	-2.728498	-4.803225	-1.580816
6	0	-3.292225	3.489397	-0.932915

1	0	-3.033710	4.367182	-1.542396
1	0	-4.366627	3.283327	-1.088280
6	0	-3.069505	3.811052	0.534962
1	0	-2.006649	4.081092	0.686569
1	0	-3.275652	2.901414	1.131764
8	0	-2.478090	2.418784	-1.354548
1	0	-2.713954	1.642653	-0.809246
8	0	-3.937463	4.874154	0.867235
1	0	-3.824523	5.072368	1.803866

- Endo product + glycol:

6	0	-0.368712	-0.657944	-2.106089
6	0	-0.127757	0.772854	-1.653082
6	0	1.570970	-0.326511	-0.961966
6	0	0.687815	-1.348500	-1.670004
6	0	-0.366788	0.790829	-0.089726
6	0	0.861401	0.021422	0.401007
1	0	-0.616558	1.591140	-2.184163
1	0	-1.282972	-1.035138	-2.560715
1	0	0.834707	-2.427291	-1.660886
8	0	1.290691	0.866989	-1.686282
6	0	-1.547278	-0.024835	0.396257
6	0	0.321154	-1.185526	1.128261
1	0	1.559211	0.589956	1.024378
1	0	-0.441327	1.825377	0.263696
7	0	-1.081393	-1.116337	1.096377
8	0	0.943724	-2.082346	1.637143
8	0	-2.730684	0.205747	0.202871
6	0	-1.922303	-2.210943	1.584303
1	0	-1.205050	-2.926245	2.010552
6	0	3.041751	-0.625890	-0.863617
1	0	3.166208	-1.617425	-0.387816
1	0	3.465725	-0.665745	-1.884407
8	0	3.664411	0.375089	-0.101163
6	0	5.050713	0.175662	0.026039
1	0	5.274004	-0.801101	0.494835
1	0	5.534961	0.185642	-0.972441
6	0	5.610080	1.297872	0.882951
1	0	5.327950	2.267187	0.434850
1	0	5.155291	1.249593	1.881947
8	0	7.001769	1.164558	1.061515
1	0	7.423917	1.323971	0.207791
6	0	-2.627385	-2.889477	0.416981
1	0	-3.194124	-3.758260	0.805680
1	0	-3.345432	-2.192624	-0.053962
6	0	-2.902005	-1.736855	2.651263
1	0	-3.467979	-2.593099	3.044318
1	0	-2.359948	-1.272511	3.485793
1	0	-3.606772	-1.005318	2.234398
8	0	-1.653279	-3.301089	-0.508777
6	0	-2.224676	-3.912956	-1.636863
1	0	-2.926426	-3.231803	-2.153391
1	0	-1.408418	-4.174615	-2.322043
1	0	-2.773081	-4.833448	-1.364445
6	0	-3.285525	3.560461	-0.822174
1	0	-3.130494	4.449990	-1.449364

1	0	-4.375842	3.421884	-0.709066
6	0	-2.686065	3.807649	0.551943
1	0	-1.604896	4.017045	0.437435
1	0	-2.792983	2.884443	1.155871
8	0	-2.672132	2.468925	-1.467174
1	0	-2.881429	1.669478	-0.949868
8	0	-3.378089	4.896941	1.123106
1	0	-3.002127	5.070893	1.993567

- Endo product:

6	0	0.863897	-2.151335	0.235357
6	0	0.121404	-1.812583	1.518325
6	0	-1.054144	-0.957026	-0.045668
6	0	0.133218	-1.611768	-0.743088
6	0	0.350221	-0.273889	1.777188
6	0	-0.504103	0.335853	0.663867
1	0	0.261566	-2.457291	2.389077
1	0	1.853507	-2.599676	0.171463
1	0	0.393463	-1.495828	-1.793878
8	0	-1.234665	-1.799218	1.091545
6	0	1.761725	0.208676	1.499006
6	0	0.455477	1.132824	-0.190210
1	0	-1.351673	0.944111	0.996176
1	0	0.041696	-0.003782	2.793168
7	0	1.728013	1.030541	0.377720
8	0	0.202345	1.737191	-1.203305
8	0	2.763689	-0.080423	2.112653
6	0	2.918330	1.555712	-0.290067
1	0	2.522733	2.079321	-1.171732
6	0	-2.313827	-0.769217	-0.845394
1	0	-2.064214	-0.191797	-1.756001
1	0	-2.697371	-1.761877	-1.147283
8	0	-3.257103	-0.084946	-0.062627
6	0	-4.470912	0.126878	-0.740292
1	0	-4.315383	0.704354	-1.670888
1	0	-4.929833	-0.843559	-1.022246
6	0	-5.401206	0.887591	0.188392
1	0	-5.498384	0.329191	1.136440
1	0	-4.956729	1.864484	0.423247
8	0	-6.644961	1.141856	-0.424110
1	0	-7.099446	0.296229	-0.527914
6	0	3.796037	0.409602	-0.776392
1	0	4.650949	0.834158	-1.338604
1	0	4.195566	-0.156699	0.085224
6	0	3.685389	2.520615	0.606905
1	0	4.538325	2.944757	0.058433
1	0	3.033503	3.346855	0.920334
1	0	4.057293	2.003584	1.501421
8	0	3.021865	-0.424689	-1.602855
6	0	3.759481	-1.519765	-2.080408
1	0	4.160509	-2.130304	-1.249670
1	0	3.084344	-2.137352	-2.686554
1	0	4.608200	-1.193843	-2.709777

Furan + Maleimide (HB: maleimide C=O + glycol, conformation 1): Exo

- Reactant 1 + glycol:

6	0	0.851373	-1.828702	-1.624921
1	0	0.813151	-2.185962	-2.651097
6	0	0.098206	-2.164747	-0.577339
1	0	-0.743176	-2.850352	-0.504348
6	0	0.562770	-1.361271	0.606706
6	0	1.823207	-0.766112	-1.191995
7	0	1.649653	-0.605651	0.186517
8	0	2.591800	-0.143463	-1.881011
8	0	0.097472	-1.357233	1.724817
6	0	2.098112	0.569621	0.934115
1	0	1.680046	0.423509	1.939989
6	0	3.615897	0.663701	0.995854
1	0	4.038472	0.761155	-0.012646
1	0	3.910515	1.539297	1.591718
1	0	4.034881	-0.233469	1.470239
6	0	1.458606	1.817698	0.329651
1	0	1.920742	2.046142	-0.649957
1	0	1.636889	2.677907	1.004019
8	0	0.085877	1.563967	0.181769
6	0	-0.609932	2.643934	-0.399703
1	0	-0.195176	2.890007	-1.394363
1	0	-1.660002	2.341013	-0.505154
1	0	-0.547390	3.545088	0.237441
8	0	-2.555135	-1.651664	0.840890
1	0	-1.803182	-1.762484	1.443447
6	0	-2.497438	-0.318693	0.376212
1	0	-1.760035	-0.195716	-0.441402
1	0	-2.203146	0.387101	1.172280
6	0	-3.868351	0.047827	-0.148114
1	0	-4.165614	-0.691986	-0.913211
1	0	-4.598208	-0.007757	0.678824
8	0	-3.776634	1.354342	-0.686843
1	0	-4.648064	1.608942	-1.011162

- Reactant complex:

6	0	-0.506369	-3.153411	0.552137
6	0	0.382203	-2.304811	1.142824
6	0	-1.603761	-1.468627	1.517348
6	0	-1.805162	-2.601653	0.790473
1	0	-0.249357	-4.047026	-0.007840
1	0	-2.767909	-2.971321	0.453587
6	0	0.428880	-0.316052	-1.285207
1	0	0.822024	-1.087970	-1.940482
6	0	-0.834999	0.031209	-1.029432
1	0	-1.773534	-0.369527	-1.406451
1	0	-2.275583	-0.718304	1.918738
8	0	-0.277942	-1.279539	1.742074
6	0	-0.817915	1.117876	0.006181
6	0	1.335729	0.548511	-0.452779
7	0	0.508818	1.373704	0.303767
8	0	2.546031	0.562048	-0.421642
8	0	-1.766311	1.671169	0.525851
6	0	1.871624	-2.265009	1.172651
1	0	2.283268	-3.094004	1.779063

1	0	2.190044	-1.313622	1.639268
8	0	2.344968	-2.357093	-0.151973
6	0	3.746123	-2.215140	-0.225666
1	0	4.259797	-3.079095	0.236947
1	0	4.059501	-1.295502	0.305482
6	0	4.125055	-2.103340	-1.690967
1	0	3.581754	-1.246334	-2.125456
1	0	3.814122	-3.014966	-2.220751
8	0	5.522067	-1.991928	-1.850763
1	0	5.780751	-1.125434	-1.511565
6	0	0.949783	2.370065	1.272798
1	0	0.017175	2.824644	1.636278
6	0	1.709011	1.719894	2.424115
1	0	2.650035	1.280313	2.063479
1	0	1.941595	2.466209	3.196812
1	0	1.097050	0.925461	2.872738
6	0	1.760944	3.454510	0.577513
1	0	2.697718	3.026752	0.173121
1	0	2.029397	4.231185	1.321058
8	0	0.972107	3.992169	-0.450530
6	0	1.653827	4.978854	-1.180781
1	0	2.568212	4.572662	-1.651255
1	0	0.975922	5.337339	-1.964747
1	0	1.943054	5.831496	-0.538493
8	0	-3.851592	-0.122712	-0.094210
1	0	-3.294363	0.637606	0.151449
6	0	-5.111437	0.355958	-0.511241
1	0	-5.038071	0.998957	-1.406779
1	0	-5.617509	0.938364	0.279156
6	0	-5.966411	-0.847280	-0.845688
1	0	-5.460462	-1.434499	-1.634521
1	0	-6.043406	-1.486396	0.053331
8	0	-7.223304	-0.360901	-1.265626
1	0	-7.783905	-1.117948	-1.469655

- Transition state:

6	0	-0.145717	-3.060528	0.037509
6	0	0.594427	-2.060037	0.725073
6	0	-1.473461	-1.499172	0.916582
6	0	-1.467491	-2.709772	0.171918
1	0	0.297977	-3.847323	-0.565737
1	0	-2.341285	-3.144014	-0.305341
6	0	0.398357	-0.574883	-0.860569
1	0	0.821765	-1.143791	-1.682185
6	0	-0.952800	-0.241431	-0.665414
1	0	-1.787446	-0.456909	-1.326557
1	0	-2.315273	-1.009612	1.398926
8	0	-0.254372	-1.359447	1.517283
6	0	-0.982245	1.032854	0.114955
6	0	1.217011	0.499358	-0.236447
7	0	0.329164	1.368104	0.406559
8	0	2.424556	0.637110	-0.232149
8	0	-1.955011	1.663332	0.488909
6	0	2.041791	-2.078235	1.105315
1	0	2.234227	-2.868162	1.856757
1	0	2.309078	-1.105245	1.553261

8	0	2.758193	-2.313731	-0.076025
6	0	4.123441	-1.967491	0.019124
1	0	4.688481	-2.712780	0.608922
1	0	4.221738	-0.978663	0.504414
6	0	4.672149	-1.888152	-1.393877
1	0	4.073252	-1.149455	-1.954518
1	0	4.560947	-2.864032	-1.887259
8	0	6.049268	-1.584022	-1.391979
1	0	6.141711	-0.670548	-1.092463
6	0	0.707839	2.518679	1.217427
1	0	-0.250610	2.954875	1.533118
6	0	1.528186	2.093007	2.430204
1	0	2.493001	1.673313	2.112579
1	0	1.715859	2.954953	3.086010
1	0	0.982371	1.330358	3.002653
6	0	1.431525	3.550892	0.362910
1	0	2.389447	3.135145	-0.001095
1	0	1.653652	4.439096	0.987851
8	0	0.593429	3.888911	-0.710379
6	0	1.201760	4.796386	-1.591264
1	0	2.130241	4.379100	-2.023016
1	0	0.489787	4.999843	-2.400322
1	0	1.451555	5.747941	-1.085490
8	0	-3.949631	-0.212600	-0.051494
1	0	-3.437783	0.600804	0.124893
6	0	-5.282929	0.134759	-0.351276
1	0	-5.354396	0.764055	-1.256206
1	0	-5.766374	0.681823	0.477460
6	0	-6.045897	-1.149989	-0.592529
1	0	-5.560913	-1.700268	-1.420460
1	0	-5.978206	-1.777833	0.315252
8	0	-7.376866	-0.792601	-0.897151
1	0	-7.879160	-1.602503	-1.041313

- Exo product + glycol:

6	0	0.001357	-3.042789	0.075099
6	0	-0.726830	-1.862113	-0.558125
6	0	1.348736	-1.498100	-0.900208
6	0	1.298278	-2.817772	-0.147551
1	0	-0.481669	-3.825112	0.657277
1	0	2.166946	-3.369593	0.205122
6	0	-0.470135	-0.654062	0.420761
1	0	-0.769590	-0.883377	1.447420
6	0	1.022288	-0.394390	0.164125
1	0	1.700052	-0.436054	1.021894
1	0	2.214336	-1.291049	-1.532240
8	0	0.128079	-1.516736	-1.640237
6	0	1.069999	0.978210	-0.472761
6	0	-1.169468	0.605679	-0.055784
7	0	-0.217735	1.466478	-0.590756
8	0	-2.356196	0.845313	-0.013516
8	0	2.061580	1.564537	-0.858362
6	0	-2.159164	-2.050632	-0.989851
1	0	-2.250193	-2.947833	-1.628287
1	0	-2.480214	-1.171204	-1.574491
8	0	-2.908943	-2.175491	0.192843

6	0	-4.261668	-1.809836	0.039875
1	0	-4.797537	-2.512486	-0.625083
1	0	-4.322439	-0.794079	-0.395251
6	0	-4.897718	-1.812242	1.417801
1	0	-4.319658	-1.134862	2.071699
1	0	-4.841825	-2.822118	1.847471
8	0	-6.264125	-1.470097	1.354342
1	0	-6.318491	-0.548174	1.071609
6	0	-0.512116	2.789211	-1.141950
1	0	0.474857	3.197624	-1.399084
6	0	-1.390110	2.693360	-2.383791
1	0	-2.369082	2.264008	-2.132119
1	0	-1.540660	3.693871	-2.813472
1	0	-0.908856	2.060065	-3.140904
6	0	-1.125906	3.673974	-0.063988
1	0	-2.140117	3.317042	0.192761
1	0	-1.210208	4.704811	-0.461393
8	0	-0.284525	3.633764	1.059001
6	0	-0.781829	4.407951	2.120116
1	0	-1.778340	4.054968	2.443452
1	0	-0.078429	4.313537	2.956204
1	0	-0.864631	5.474302	1.839344
8	0	3.977843	-0.380643	-0.174928
1	0	3.531615	0.436742	-0.463508
6	0	5.256115	-0.057889	0.324703
1	0	5.203872	0.613738	1.200431
1	0	5.888808	0.431785	-0.436738
6	0	5.919557	-1.352012	0.743907
1	0	5.284083	-1.847024	1.501996
1	0	5.977855	-2.019854	-0.135253
8	0	7.194776	-1.021295	1.250623
1	0	7.637440	-1.839118	1.504055

- Exo product:

6	0	-1.993752	2.650257	-0.773998
6	0	-1.376040	1.591062	0.131566
6	0	0.118077	3.100201	-0.073582
6	0	-1.062871	3.599308	-0.894298
1	0	-2.959306	2.554918	-1.266826
1	0	-1.072883	4.493420	-1.514515
6	0	-0.322898	0.848359	-0.774492
1	0	-0.766922	0.481558	-1.704239
6	0	0.762247	1.931296	-0.888899
1	0	1.061262	2.221385	-1.901972
1	0	0.829917	3.825919	0.329543
8	0	-0.521556	2.363657	0.967296
6	0	1.953568	1.369020	-0.132395
6	0	0.345327	-0.294995	-0.030504
7	0	1.620142	0.098573	0.333252
8	0	-0.145669	-1.372190	0.235883
8	0	3.003412	1.922814	0.073466
6	0	-2.299292	0.695079	0.918258
1	0	-3.030742	1.301248	1.482454
1	0	-1.704864	0.104931	1.637084
8	0	-2.926792	-0.135387	-0.025913
6	0	-3.355760	-1.370856	0.501479

1	0	-4.216289	-1.242506	1.183976
1	0	-2.525515	-1.842312	1.060236
6	0	-3.744611	-2.257490	-0.667367
1	0	-2.876299	-2.339233	-1.345295
1	0	-4.567105	-1.790582	-1.227076
8	0	-4.210515	-3.513595	-0.227433
1	0	-3.461845	-3.975069	0.171804
6	0	2.566960	-0.732031	1.074597
1	0	3.480699	-0.122919	1.114431
6	0	2.063358	-1.030461	2.481799
1	0	1.134802	-1.615968	2.442248
1	0	2.819846	-1.601582	3.038624
1	0	1.869771	-0.093846	3.021537
6	0	2.873820	-2.001063	0.289527
1	0	1.984493	-2.656979	0.259989
1	0	3.690103	-2.544890	0.805105
8	0	3.259486	-1.626941	-1.007555
6	0	3.543967	-2.739768	-1.815007
1	0	2.664318	-3.402529	-1.912040
1	0	3.823260	-2.365906	-2.807545
1	0	4.382776	-3.333455	-1.406339

Furan + Maleimide (HB: maleimide C=O + glycol, conformation 2): Exo

- Reactant 1 + glycol:

6	0	0.203200	2.045225	-1.161350
1	0	0.065953	2.930827	-1.776942
6	0	-0.554229	0.952225	-1.062152
1	0	-1.479378	0.673734	-1.565286
6	0	0.095553	0.025213	-0.069232
6	0	1.381238	1.897315	-0.238409
7	0	1.205054	0.674542	0.427861
8	0	2.299189	2.654351	-0.071747
8	0	-0.268268	-1.092593	0.245695
6	0	2.249145	0.056329	1.240426
1	0	3.029844	0.827824	1.302854
6	0	1.740619	-0.306173	2.629582
1	0	0.916958	-1.029770	2.559246
1	0	2.552395	-0.750707	3.222870
1	0	1.381529	0.590052	3.152137
6	0	2.825147	-1.151512	0.508220
1	0	2.093531	-1.981476	0.507246
1	0	3.733483	-1.492331	1.042849
8	0	3.127840	-0.756571	-0.804014
6	0	3.637452	-1.816233	-1.573258
1	0	2.914353	-2.649881	-1.637064
1	0	3.830189	-1.429291	-2.581067
1	0	4.582115	-2.205194	-1.150714
8	0	-2.806957	-1.134478	-0.962801
1	0	-1.936705	-1.416759	-0.633536
6	0	-3.494116	-0.584975	0.140094
1	0	-3.033928	0.361642	0.486924
1	0	-3.517159	-1.275194	1.001523
6	0	-4.913680	-0.300106	-0.299823
1	0	-4.885574	0.372189	-1.177619
1	0	-5.382165	-1.248719	-0.619030

8	0	-5.580402	0.285184	0.798685
1	0	-6.494017	0.450266	0.539465

- Reactant complex:

6	0	0.015495	-2.429638	1.603210
6	0	0.743482	-1.277340	1.579522
6	0	-1.353128	-0.674874	1.752780
6	0	-1.356566	-2.034889	1.709655
1	0	0.421897	-3.432857	1.523194
1	0	-2.238584	-2.666581	1.732366
6	0	0.410404	-0.823848	-1.503302
1	0	0.837434	-1.791656	-1.750104
6	0	-0.856509	-0.483264	-1.253707
1	0	-1.764189	-1.082777	-1.229743
1	0	-2.144190	0.064224	1.814235
8	0	-0.081500	-0.201691	1.679774
6	0	-0.884469	0.974153	-0.894949
6	0	1.267126	0.405022	-1.371818
7	0	0.405831	1.450190	-1.015011
8	0	2.453594	0.523986	-1.552002
8	0	-1.847140	1.636781	-0.549658
6	0	2.198782	-1.016345	1.386564
1	0	2.775674	-1.294418	2.290100
1	0	2.343205	0.062536	1.198854
8	0	2.635969	-1.787668	0.286585
6	0	3.996453	-1.559467	-0.006716
1	0	4.649045	-1.968355	0.788057
1	0	4.187136	-0.472397	-0.090547
6	0	4.305236	-2.229158	-1.333009
1	0	3.621692	-1.819162	-2.096699
1	0	4.123053	-3.310113	-1.250563
8	0	5.661929	-2.067461	-1.684201
1	0	5.795152	-1.131663	-1.883061
6	0	0.851482	2.801809	-0.685231
1	0	1.922957	2.804296	-0.932589
6	0	0.113134	3.852895	-1.507019
1	0	-0.951650	3.880760	-1.240490
1	0	0.552516	4.843610	-1.326201
1	0	0.200557	3.626697	-2.578109
6	0	0.725214	3.041697	0.812682
1	0	-0.303410	2.805908	1.145386
1	0	0.919397	4.110630	1.028063
8	0	1.663634	2.229323	1.470466
6	0	1.505970	2.263364	2.868093
1	0	0.503748	1.908509	3.164296
1	0	2.263630	1.597192	3.301073
1	0	1.659942	3.284331	3.262992
8	0	-3.796103	-0.340196	-0.079101
1	0	-3.284492	0.470570	-0.252939
6	0	-5.086989	-0.188377	-0.627369
1	0	-5.061740	-0.054903	-1.724025
1	0	-5.622804	0.676262	-0.196662
6	0	-5.866516	-1.445854	-0.308631
1	0	-5.332144	-2.313808	-0.737508
1	0	-5.893151	-1.575540	0.789001
8	0	-7.156023	-1.286842	-0.860679

1	0	-7.667980	-2.077784	-0.657296

- Transition state:

6	0	0.222807	-2.578626	0.983693
6	0	0.848020	-1.302471	1.043612
6	0	-1.271080	-0.926182	1.078759
6	0	-1.129599	-2.339915	1.026700
1	0	0.751851	-3.511601	0.811988
1	0	-1.949705	-3.038649	0.888578
6	0	0.428509	-0.858266	-1.069181
1	0	0.843153	-1.746021	-1.536344
6	0	-0.932583	-0.534706	-0.924166
1	0	-1.785137	-1.070168	-1.333291
1	0	-2.157582	-0.348293	1.327012
8	0	-0.067261	-0.389577	1.446992
6	0	-1.014924	0.956712	-0.824461
6	0	1.190310	0.411938	-1.143418
7	0	0.265991	1.456411	-0.937400
8	0	2.371823	0.593786	-1.333332
8	0	-2.012428	1.637740	-0.632301
6	0	2.294530	-0.990721	1.265650
1	0	2.579642	-1.239502	2.307160
1	0	2.458019	0.088303	1.105601
8	0	3.006282	-1.774092	0.344220
6	0	4.321502	-1.313042	0.116644
1	0	4.996026	-1.593937	0.946659
1	0	4.316996	-0.212248	0.017377
6	0	4.801994	-1.930288	-1.184136
1	0	4.092661	-1.646763	-1.980932
1	0	4.799059	-3.026068	-1.096269
8	0	6.127955	-1.545955	-1.474021
1	0	6.112007	-0.605042	-1.691464
6	0	0.677510	2.819922	-0.616492
1	0	1.750616	2.842257	-0.855047
6	0	-0.067906	3.861490	-1.442343
1	0	-1.134815	3.876755	-1.186751
1	0	0.362240	4.856158	-1.259202
1	0	0.030897	3.634586	-2.512266
6	0	0.530220	3.043479	0.882072
1	0	-0.513630	2.834581	1.186501
1	0	0.755484	4.099497	1.127389
8	0	1.424037	2.179681	1.541073
6	0	1.188945	2.124158	2.925890
1	0	0.174918	1.744748	3.142948
1	0	1.927342	1.436278	3.358392
1	0	1.309498	3.117674	3.395244
8	0	-3.906764	-0.349657	-0.165606
1	0	-3.430055	0.474390	-0.386308
6	0	-5.263364	-0.211730	-0.523874
1	0	-5.391464	-0.043331	-1.608096
1	0	-5.750450	0.626176	0.005956
6	0	-5.968281	-1.496876	-0.146720
1	0	-5.479886	-2.337132	-0.674550
1	0	-5.844243	-1.663124	0.939428
8	0	-7.323294	-1.351089	-0.513887
1	0	-7.787831	-2.160345	-0.272401

- Exo product + glycol:

6	0	0.250905	-2.684202	0.941022
6	0	0.871433	-1.298875	0.805234
6	0	-1.224778	-0.960121	1.036412
6	0	-1.059719	-2.469722	1.081576
1	0	0.797209	-3.618995	0.831011
1	0	-1.875747	-3.187878	1.123473
6	0	0.476257	-0.846758	-0.660761
1	0	0.733872	-1.608518	-1.401140
6	0	-1.012051	-0.527617	-0.454147
1	0	-1.734782	-0.985128	-1.136714
1	0	-2.095232	-0.516550	1.523699
8	0	0.004426	-0.497971	1.597371
6	0	-1.076523	0.987890	-0.520253
6	0	1.121299	0.476276	-1.008333
7	0	0.158507	1.485461	-0.872109
8	0	2.266229	0.677187	-1.329773
8	0	-2.049414	1.675957	-0.262504
6	0	2.321685	-1.099781	1.165817
1	0	2.514988	-1.479107	2.185990
1	0	2.546995	-0.018683	1.137627
8	0	3.060310	-1.804024	0.198913
6	0	4.363934	-1.302157	0.004674
1	0	5.021030	-1.541949	0.861229
1	0	4.324617	-0.203363	-0.115469
6	0	4.911025	-1.928736	-1.264738
1	0	4.216287	-1.698481	-2.091885
1	0	4.950436	-3.021108	-1.150042
8	0	6.228130	-1.496992	-1.525359
1	0	6.185256	-0.555878	-1.738372
6	0	0.557187	2.888749	-0.734208
1	0	1.593490	2.910075	-1.098808
6	0	-0.311257	3.837351	-1.548188
1	0	-1.345014	3.840106	-1.181504
1	0	0.097601	4.855299	-1.481317
1	0	-0.313282	3.538296	-2.604936
6	0	0.571551	3.216306	0.755185
1	0	-0.453218	3.121093	1.160826
1	0	0.907201	4.260688	0.903163
8	0	1.442035	2.308706	1.384496
6	0	1.224511	2.224014	2.772842
1	0	0.201688	1.873411	2.992488
1	0	1.939087	1.492769	3.171274
1	0	1.392695	3.198219	3.266975
8	0	-3.961241	-0.353154	0.057790
1	0	-3.502947	0.502423	-0.034090
6	0	-5.254486	-0.245368	-0.493054
1	0	-5.229672	-0.001933	-1.570649
1	0	-5.858753	0.529246	0.011854
6	0	-5.938173	-1.583635	-0.313841
1	0	-5.330743	-2.361878	-0.812504
1	0	-5.970917	-1.821906	0.765211
8	0	-7.227620	-1.470658	-0.876809
1	0	-7.681345	-2.312590	-0.757079

- Exo product:

6	0	-0.899267	3.006540	0.023467
6	0	-0.579443	1.548164	0.327882
6	0	1.311799	2.487987	0.013715
6	0	0.283857	3.593683	-0.173818
1	0	-1.904067	3.406511	-0.097889
1	0	0.497004	4.611946	-0.492838
6	0	-0.172952	0.945469	-1.078265
1	0	-0.916604	1.176554	-1.845446
6	0	1.228764	1.553761	-1.236231
1	0	1.445489	2.056254	-2.185358
1	0	2.329378	2.750623	0.316157
8	0	0.682507	1.645646	0.978680
6	0	2.172171	0.381405	-1.003005
6	0	0.052086	-0.549052	-0.984558
7	0	1.425138	-0.786183	-0.926592
8	0	-0.790812	-1.413173	-0.940403
8	0	3.370442	0.451730	-0.858583
6	0	-1.567254	0.725995	1.116437
1	0	-1.819747	1.240026	2.061914
1	0	-1.104494	-0.248813	1.350920
8	0	-2.691627	0.576172	0.286276
6	0	-3.435083	-0.597864	0.532271
1	0	-4.051631	-0.501806	1.445026
1	0	-2.747229	-1.454645	0.655622
6	0	-4.320819	-0.838855	-0.676433
1	0	-3.673881	-0.901570	-1.569106
1	0	-5.004488	0.011444	-0.809924
8	0	-5.123433	-1.986081	-0.504877
1	0	-4.533615	-2.750941	-0.506499
6	0	1.945735	-2.057230	-0.418771
1	0	1.088639	-2.742633	-0.474767
6	0	3.108236	-2.590946	-1.243896
1	0	3.970460	-1.914679	-1.191195
1	0	3.402692	-3.580580	-0.867084
1	0	2.809116	-2.699661	-2.295055
6	0	2.300616	-1.858221	1.051319
1	0	3.116220	-1.116077	1.133130
1	0	2.655949	-2.814737	1.480397
8	0	1.146482	-1.403046	1.715221
6	0	1.440067	-0.779153	2.942306
1	0	2.080596	0.106764	2.792430
1	0	0.487610	-0.453905	3.379936
1	0	1.937097	-1.476871	3.640751

Furan + Maleimide (HB: furan -O- + glycol, conformation 1): Endo

- Reactant 1 + glycol:

6	0	2.970642	-1.651143	-0.282060
6	0	2.516229	-1.288720	0.948146
6	0	0.754408	-1.920260	-0.174966
6	0	1.811466	-2.061265	-1.021628
1	0	2.999130	-0.906325	1.840578
1	0	3.999441	-1.620413	-0.625128
1	0	1.766052	-2.401319	-2.051522

6	0	-0.714129	-2.085369	-0.316573
1	0	-0.942297	-2.562088	-1.284715
1	0	-1.111793	-2.725265	0.492637
8	0	-1.297515	-0.797968	-0.238017
6	0	-2.706198	-0.800491	-0.144951
1	0	-3.157176	-1.369937	-0.977684
1	0	-3.028452	-1.275179	0.803908
6	0	-3.157667	0.651807	-0.189366
1	0	-2.575389	1.230113	0.549979
1	0	-2.929899	1.067376	-1.180871
8	0	-4.551341	0.759790	-0.008736
1	0	-4.740985	0.590698	0.922590
8	0	-0.003307	1.317691	1.091223
1	0	-0.409576	0.456948	0.909707
8	0	1.176795	-1.457022	1.028885
6	0	0.687472	1.664264	-0.088656
1	0	-0.001432	1.938720	-0.909438
1	0	1.325769	0.837277	-0.453834
6	0	1.565555	2.853812	0.231167
1	0	0.929225	3.669831	0.620841
1	0	2.270889	2.565880	1.032972
8	0	2.232435	3.220110	-0.959628
1	0	2.783291	3.987062	-0.766521

- Reactant complex:

6	0	0.217531	1.876461	2.545384
6	0	-1.024313	2.230025	2.116968
6	0	-1.008519	0.046506	2.164836
6	0	0.227498	0.442363	2.578062
6	0	0.619884	1.156652	-1.117184
6	0	0.269266	-0.063935	-0.704134
1	0	-1.507693	3.181645	1.924769
1	0	1.030123	2.552262	2.792685
1	0	1.051450	-0.215056	2.837588
6	0	2.030304	1.413562	-0.665854
6	0	1.421258	-0.674226	0.038881
1	0	-0.678283	-0.589400	-0.802811
1	0	0.032261	1.887795	-1.674276
7	0	2.424361	0.282167	0.064175
8	0	1.478249	-1.776637	0.541830
8	0	2.714312	2.385761	-0.855524
6	0	3.798858	0.052311	0.488744
1	0	4.322990	0.981199	0.221217
6	0	-1.636296	-1.285501	1.948625
1	0	-0.837933	-2.045938	1.900752
1	0	-2.325579	-1.541229	2.776011
8	0	-2.362980	-1.239293	0.732909
6	0	-3.056006	-2.442075	0.457257
1	0	-2.370290	-3.306612	0.502039
1	0	-3.853697	-2.602213	1.210228
6	0	-3.661334	-2.323416	-0.933217
1	0	-4.246758	-1.389677	-0.995621
1	0	-2.850208	-2.258995	-1.673592
8	0	-4.413660	-3.472341	-1.253172
1	0	-5.246072	-3.422894	-0.766957
6	0	4.408842	-1.089541	-0.317067

1	0	5.490115	-1.156420	-0.083712
1	0	3.934289	-2.048195	-0.034202
6	0	3.893032	-0.200249	1.988514
1	0	4.942290	-0.339834	2.286154
1	0	3.488557	0.655644	2.546312
1	0	3.328308	-1.104178	2.258356
8	0	4.201962	-0.816620	-1.677680
6	0	4.673712	-1.849124	-2.503949
1	0	4.167321	-2.806148	-2.280406
1	0	4.461891	-1.564401	-3.541637
1	0	5.763589	-1.996401	-2.387124
8	0	-3.939254	0.934196	-0.309131
1	0	-3.576719	0.359296	0.382805
8	0	-1.783438	1.127697	1.898889
6	0	-2.834282	1.258806	-1.119606
1	0	-2.572532	0.434388	-1.812839
1	0	-1.945098	1.476387	-0.508109
6	0	-3.148102	2.492425	-1.936094
1	0	-4.001161	2.294866	-2.609137
1	0	-3.433925	3.311625	-1.252170
8	0	-1.964821	2.790809	-2.657473
1	0	-2.128733	3.562467	-3.211723

- Transition state:

6	0	-0.778809	-0.344894	2.835517
6	0	0.237712	-1.147856	2.258811
6	0	0.621254	0.855659	1.577800
6	0	-0.536178	0.938117	2.398384
6	0	-0.534555	-1.269310	0.274266
6	0	-0.242225	0.029979	-0.160398
1	0	0.529320	-2.163385	2.513120
1	0	-1.646765	-0.720061	3.370847
1	0	-1.170656	1.814397	2.503996
6	0	-2.019557	-1.360296	0.437632
6	0	-1.526418	0.780724	-0.263480
1	0	0.605425	0.324077	-0.775119
1	0	0.028499	-2.165728	0.020398
7	0	-2.525135	-0.077241	0.182361
8	0	-1.693598	1.931697	-0.613044
8	0	-2.690186	-2.308642	0.764133
6	0	-3.945900	0.239968	0.227944
1	0	-4.425281	-0.706243	0.517464
6	0	1.491136	1.974376	1.101076
1	0	0.842851	2.736306	0.631588
1	0	2.025417	2.436537	1.952813
8	0	2.414363	1.450579	0.176196
6	0	3.322095	2.416949	-0.322725
1	0	2.777540	3.297979	-0.705572
1	0	3.996577	2.758093	0.487334
6	0	4.124451	1.768235	-1.440637
1	0	4.562005	0.825392	-1.072349
1	0	3.445661	1.519017	-2.268870
8	0	5.083463	2.667263	-1.951352
1	0	5.812216	2.709331	-1.319799
6	0	-4.445920	0.618757	-1.161425
1	0	-5.546867	0.741933	-1.122316

1	0	-3.999489	1.580482	-1.475037
6	0	-4.240195	1.329308	1.253366
1	0	-5.317989	1.544612	1.285197
1	0	-3.926002	1.005226	2.255465
1	0	-3.703627	2.252183	0.990673
8	0	-4.086369	-0.408746	-2.046465
6	0	-4.438542	-0.111957	-3.372147
1	0	-3.942112	0.811392	-3.723514
1	0	-4.114832	-0.953677	-3.996389
1	0	-5.531580	0.017434	-3.483141
8	0	3.863340	-0.997046	0.375837
1	0	3.351700	-0.217024	0.641811
8	0	1.245519	-0.329769	1.840765
6	0	2.930496	-1.913494	-0.140965
1	0	2.210351	-1.430520	-0.828202
1	0	2.345322	-2.403068	0.660879
6	0	3.669097	-2.994244	-0.897949
1	0	4.208488	-2.542607	-1.750281
1	0	4.418019	-3.452428	-0.226464
8	0	2.687360	-3.920766	-1.317063
1	0	3.122965	-4.615636	-1.823544

- Endo product + glycol:

6	0	-0.843413	-1.027680	-2.779113
6	0	0.025285	0.170229	-2.421168
6	0	0.464900	-1.311452	-0.937606
6	0	-0.571107	-1.951978	-1.852979
6	0	-0.600122	0.832153	-1.138658
6	0	-0.277471	-0.224737	-0.080032
1	0	0.308898	0.862013	-3.217550
1	0	-1.599314	-1.051472	-3.561766
1	0	-1.052552	-2.914895	-1.689116
6	0	-2.114520	0.930419	-1.144679
6	0	-1.616300	-0.678605	0.457971
1	0	0.383009	0.108829	0.728041
1	0	-0.153043	1.815439	-0.952809
7	0	-2.608572	0.043822	-0.186506
8	0	-1.807963	-1.553101	1.273766
8	0	-2.801074	1.612802	-1.863074
6	0	-4.040466	-0.125019	0.051528
1	0	-4.506597	0.680082	-0.533355
6	0	1.414087	-2.195192	-0.174945
1	0	0.843110	-2.786488	0.564881
1	0	1.921978	-2.885004	-0.874523
8	0	2.340952	-1.344742	0.450340
6	0	3.378004	-2.025556	1.123753
1	0	2.968687	-2.717432	1.882434
1	0	3.970992	-2.624945	0.403768
6	0	4.261770	-0.979473	1.784129
1	0	4.583019	-0.257116	1.015329
1	0	3.670796	-0.435448	2.535028
8	0	5.337662	-1.585649	2.467531
1	0	5.979760	-1.870039	1.805351
6	0	-4.360178	0.102395	1.523737
1	0	-5.461767	0.114684	1.644183
1	0	-3.953178	-0.722378	2.136153

6	0	-4.521306	-1.486390	-0.438090
1	0	-5.604652	-1.586091	-0.279558
1	0	-4.318961	-1.596793	-1.512395
1	0	-4.008272	-2.290993	0.107106
8	0	-3.797983	1.330242	1.905732
6	0	-3.995765	1.598877	3.269962
1	0	-3.538274	0.817248	3.903804
1	0	-3.521052	2.562938	3.489054
1	0	-5.071436	1.663600	3.518752
8	0	3.724132	0.756791	-1.004253
1	0	3.132394	-0.002205	-1.121458
8	0	1.169435	-0.462255	-1.848676
6	0	2.898677	1.810930	-0.575735
1	0	2.250578	1.510545	0.269711
1	0	2.241721	2.178514	-1.388083
6	0	3.784069	2.953829	-0.129838
1	0	4.410563	2.616921	0.716740
1	0	4.458371	3.225291	-0.962487
8	0	2.926790	4.016991	0.233321
1	0	3.472753	4.753315	0.531386

- Endo product:

6	0	-0.258384	-2.935004	0.391146
6	0	0.253004	-2.581510	-0.998443
6	0	1.268086	-1.244807	0.322362
6	0	0.374729	-2.098771	1.218335
6	0	-0.403421	-1.208358	-1.400344
6	0	0.327929	-0.252656	-0.455229
1	0	0.227284	-3.354533	-1.769907
1	0	-1.055530	-3.641378	0.615360
1	0	0.219956	-1.949406	2.286092
6	0	-1.871137	-1.066796	-1.045650
6	0	-0.752166	0.371253	0.399625
1	0	0.944179	0.510536	-0.941656
1	0	-0.246735	-1.007034	-2.465687
7	0	-1.976950	-0.133339	-0.013956
8	0	-0.593666	1.142082	1.320329
8	0	-2.801935	-1.666589	-1.523528
6	0	-3.261135	0.217516	0.587658
1	0	-4.002890	-0.273875	-0.057298
6	0	2.486648	-0.625560	0.950253
1	0	2.160182	-0.019021	1.816704
1	0	3.153291	-1.431526	1.310422
8	0	3.136614	0.173958	-0.002443
6	0	4.293376	0.791061	0.506482
1	0	4.057984	1.422653	1.383626
1	0	5.027267	0.025364	0.833272
6	0	4.893957	1.645406	-0.596287
1	0	5.067623	1.014439	-1.486019
1	0	4.174903	2.426398	-0.879229
8	0	6.060904	2.303785	-0.158823
1	0	6.743652	1.634099	-0.025589
6	0	-3.484415	1.722713	0.515309
1	0	-4.519223	1.939168	0.847815
1	0	-2.785467	2.246841	1.191781
6	0	-3.361949	-0.311407	2.014055

1	0	-4.345745	-0.068914	2.440804
1	0	-3.242261	-1.403610	2.022494
1	0	-2.581630	0.138113	2.643860
8	0	-3.288463	2.128470	-0.814271
6	0	-3.428715	3.517267	-0.966541
1	0	-2.698379	4.066032	-0.344011
1	0	-3.250387	3.753986	-2.022548
1	0	-4.444929	3.855138	-0.690010
8	0	1.583680	-2.155729	-0.729885

Furan + Maleimide (HB: furan -O- + glycol, conformation 2): Endo

- Reactant 1 + glycol:

6	0	2.970380	-1.651606	-0.282102
6	0	2.516119	-1.288874	0.948065
6	0	0.754110	-1.920315	-0.174822
6	0	1.811091	-2.061681	-1.021519
1	0	2.999126	-0.906409	1.840409
1	0	3.999162	-1.621135	-0.625246
1	0	1.765563	-2.401912	-2.051349
6	0	-0.714461	-2.085285	-0.316262
1	0	-0.942798	-2.562358	-1.284189
1	0	-1.112087	-2.724823	0.493252
8	0	-1.297687	-0.797793	-0.238130
6	0	-2.706354	-0.800161	-0.144775
1	0	-3.157545	-1.369870	-0.977214
1	0	-3.028447	-1.274495	0.804317
6	0	-3.157780	0.652136	-0.189611
1	0	-2.575390	1.230691	0.549448
1	0	-2.930176	1.067364	-1.181296
8	0	-4.551425	0.760193	-0.008801
1	0	-4.740930	0.591301	0.922590
8	0	-0.003445	1.318026	1.090932
1	0	-0.409873	0.457347	0.909461
8	0	1.176645	-1.456924	1.028915
6	0	0.687914	1.664154	-0.088748
1	0	-0.000579	1.938435	-0.909929
1	0	1.326291	0.836984	-0.453368
6	0	1.565988	2.853698	0.231121
1	0	0.929562	3.669917	0.620226
1	0	2.270874	2.565932	1.033383
8	0	2.233515	3.219521	-0.959448
1	0	2.784392	3.986453	-0.766326

- Reactant complex:

6	0	1.179356	1.149401	1.667164
6	0	0.054278	1.866953	1.398690
6	0	-0.597844	-0.201487	1.661955
6	0	0.753187	-0.207592	1.836934
6	0	0.463472	0.907374	-1.504276
6	0	-0.055314	-0.306588	-1.297622
1	0	-0.132073	2.901367	1.129615
1	0	2.196668	1.526841	1.691804
1	0	1.388199	-1.073478	1.997382
6	0	1.959964	0.805402	-1.391131

6	0	1.062612	-1.266046	-1.011365
1	0	-1.093811	-0.628667	-1.259353
1	0	-0.038286	1.858749	-1.683178
7	0	2.243464	-0.525172	-1.087588
8	0	0.990387	-2.444059	-0.757011
8	0	2.780851	1.681947	-1.532497
6	0	3.574915	-1.103793	-0.954100
1	0	3.391715	-2.151696	-0.674553
6	0	-1.627275	-1.274092	1.703401
1	0	-1.115156	-2.248802	1.631396
1	0	-2.196732	-1.247472	2.652490
8	0	-2.520883	-1.096408	0.618228
6	0	-3.506017	-2.113067	0.551857
1	0	-3.030996	-3.109901	0.545988
1	0	-4.169398	-2.057792	1.438358
6	0	-4.312486	-1.914136	-0.722433
1	0	-4.710707	-0.886442	-0.744834
1	0	-3.647314	-2.038078	-1.589581
8	0	-5.315855	-2.899868	-0.832751
1	0	-6.027639	-2.662223	-0.225641
6	0	4.357298	-0.447543	0.174749
1	0	5.374378	-0.885431	0.196280
1	0	4.453004	0.638445	-0.010265
6	0	4.338619	-1.029972	-2.273976
1	0	5.312151	-1.531936	-2.182337
1	0	3.766943	-1.527086	-3.068966
1	0	4.503850	0.017708	-2.561936
8	0	3.698000	-0.688359	1.394753
6	0	4.392719	-0.139649	2.485759
1	0	4.535939	0.950742	2.366433
1	0	3.794813	-0.325625	3.387212
1	0	5.386878	-0.607765	2.607336
8	0	-4.018293	1.267312	0.079650
1	0	-3.505285	0.611577	0.578346
8	0	-1.037660	1.059843	1.413760
6	0	-3.086867	1.900987	-0.768424
1	0	-3.551428	2.065826	-1.753341
1	0	-2.191518	1.275490	-0.914087
6	0	-2.632057	3.228160	-0.181625
1	0	-3.436369	3.978586	-0.265313
1	0	-2.418936	3.075188	0.891406
8	0	-1.467546	3.627235	-0.892607
1	0	-1.326523	4.571088	-0.753092

- Transition state:

6	0	-1.418923	-0.085857	1.965657
6	0	-0.272727	-0.910114	1.834617
6	0	0.283887	0.999740	1.018871
6	0	-1.066740	1.136199	1.441889
6	0	-0.442218	-1.317892	-0.251205
6	0	-0.031650	-0.074605	-0.755037
1	0	-0.062724	-1.870354	2.298490
1	0	-2.413277	-0.423121	2.244097
1	0	-1.734279	1.958951	1.200046
6	0	-1.913218	-1.434367	-0.496555
6	0	-1.229155	0.613568	-1.314552

1	0	0.956522	0.162573	-1.142558
1	0	0.164782	-2.220967	-0.218915
7	0	-2.319805	-0.225637	-1.067974
8	0	-1.296563	1.698854	-1.846121
8	0	-2.652956	-2.359137	-0.241146
6	0	-3.696089	0.138837	-1.381090
1	0	-3.627639	1.172220	-1.751104
6	0	1.251507	2.083077	0.665660
1	0	0.766236	2.749167	-0.070462
1	0	1.511560	2.674327	1.564077
8	0	2.407293	1.483855	0.128244
6	0	3.418037	2.415061	-0.213812
1	0	3.006796	3.215282	-0.854031
1	0	3.825785	2.886927	0.702000
6	0	4.513407	1.661355	-0.952825
1	0	4.827717	0.794266	-0.348261
1	0	4.105946	1.277062	-1.898943
8	0	5.577216	2.525755	-1.284635
1	0	6.088636	2.683648	-0.481379
6	0	-4.560539	0.141633	-0.128059
1	0	-5.598329	0.398100	-0.416832
1	0	-4.568553	-0.865036	0.330101
6	0	-4.278581	-0.772404	-2.458188
1	0	-5.289969	-0.440495	-2.732731
1	0	-3.648750	-0.740825	-3.357131
1	0	-4.326043	-1.808953	-2.097648
8	0	-4.058525	1.096494	0.779583
6	0	-4.847004	1.179269	1.939583
1	0	-4.904923	0.204440	2.459735
1	0	-4.381141	1.915058	2.607628
1	0	-5.876729	1.506333	1.705765
8	0	3.734938	-0.858580	1.065217
1	0	3.166990	-0.071998	1.065840
8	0	0.810189	-0.110759	1.617114
6	0	2.993839	-1.869918	0.429261
1	0	2.509889	-1.509958	-0.498370
1	0	2.196583	-2.271165	1.083744
6	0	3.920352	-3.012123	0.077824
1	0	4.687954	-2.657173	-0.633592
1	0	4.437099	-3.347905	0.995393
8	0	3.103982	-4.023768	-0.477829
1	0	3.668788	-4.761413	-0.734782

- Endo product + glycol:

6	0	-1.445745	-0.434670	-2.058183
6	0	-0.373309	0.623970	-1.856046
6	0	0.220403	-1.029327	-0.626109
6	0	-1.080135	-1.462472	-1.287507
6	0	-0.593584	1.220687	-0.415531
6	0	-0.154394	0.044074	0.458911
1	0	-0.202079	1.356516	-2.648013
1	0	-2.378630	-0.296635	-2.601153
1	0	-1.652946	-2.352798	-1.034449
6	0	-2.049294	1.455964	-0.053133
6	0	-1.371000	-0.327657	1.277029
1	0	0.716742	0.231524	1.096373

1	0	-0.006988	2.136155	-0.278231
7	0	-2.401015	0.554931	0.946318
8	0	-1.468720	-1.238338	2.062173
8	0	-2.801176	2.260193	-0.553579
6	0	-3.764348	0.374577	1.444846
1	0	-3.683879	-0.466520	2.147701
6	0	1.206243	-2.075842	-0.184194
1	0	0.753823	-2.682005	0.622752
1	0	1.447565	-2.736202	-1.038098
8	0	2.350655	-1.393769	0.258759
6	0	3.441023	-2.239398	0.549280
1	0	3.189790	-2.949343	1.358727
1	0	3.712912	-2.831616	-0.348008
6	0	4.609434	-1.358893	0.961168
1	0	4.792358	-0.627637	0.156038
1	0	4.336990	-0.804757	1.871220
8	0	5.743915	-2.136928	1.277699
1	0	6.115509	-2.459757	0.447349
6	0	-4.687750	-0.045982	0.308459
1	0	-5.690048	-0.257711	0.729810
1	0	-4.787481	0.774424	-0.425928
6	0	-4.270318	1.622977	2.158203
1	0	-5.272696	1.438645	2.569894
1	0	-3.600635	1.883859	2.988362
1	0	-4.320592	2.471459	1.462967
8	0	-4.150954	-1.194569	-0.301101
6	0	-4.945255	-1.639442	-1.370411
1	0	-5.057094	-0.856505	-2.143790
1	0	-4.449679	-2.513235	-1.812695
1	0	-5.954825	-1.935030	-1.030254
8	0	3.556903	0.681898	-1.445801
1	0	2.798295	0.084291	-1.536291
8	0	0.786589	-0.174836	-1.625993
6	0	3.139198	1.681269	-0.549083
1	0	2.744482	1.250778	0.390655
1	0	2.350076	2.325545	-0.984300
6	0	4.333892	2.552672	-0.227829
1	0	5.111178	1.935627	0.260129
1	0	4.754223	2.939349	-1.174139
8	0	3.873234	3.588517	0.616090
1	0	4.626828	4.144675	0.843950

- Endo product:

6	0	0.863651	-2.151505	0.235673
6	0	0.121189	-1.812422	1.518572
6	0	-1.054127	-0.956842	-0.045614
6	0	0.133113	-1.611926	-0.742869
6	0	0.350277	-0.273789	1.777211
6	0	-0.503872	0.335973	0.663725
1	0	0.261225	-2.457054	2.389400
1	0	1.853134	-2.600142	0.171871
1	0	0.393384	-1.496307	-1.793687
6	0	1.761848	0.208579	1.499143
6	0	0.455886	1.132784	-0.190280
1	0	-1.351299	0.944435	0.996045
1	0	0.041702	-0.003419	2.793110

7	0	1.728325	1.030571	0.377919
8	0	0.202928	1.737060	-1.203469
8	0	2.763751	-0.080636	2.112845
6	0	2.918750	1.555548	-0.289809
1	0	2.523262	2.079535	-1.171301
6	0	-2.313820	-0.768890	-0.845317
1	0	-2.064164	-0.191364	-1.755854
1	0	-2.697402	-1.761489	-1.147343
8	0	-3.257083	-0.084648	-0.062508
6	0	-4.470447	0.128102	-0.740681
1	0	-4.314464	0.707672	-1.669904
1	0	-4.928720	-0.841914	-1.025092
6	0	-5.401766	0.886425	0.188927
1	0	-5.499852	0.325697	1.135507
1	0	-4.957589	1.862758	0.426648
8	0	-6.644946	1.142093	-0.424156
1	0	-7.099557	0.296773	-0.529851
6	0	3.795901	0.409207	-0.776654
1	0	4.650988	0.833505	-1.338784
1	0	4.195247	-0.157606	0.084731
6	0	3.686270	2.519873	0.607369
1	0	4.539198	2.943962	0.058846
1	0	3.034669	3.346141	0.921290
1	0	4.058209	2.002373	1.501600
8	0	3.021232	-0.424402	-1.603329
6	0	3.758269	-1.519629	-2.081441
1	0	4.159316	-2.130562	-1.251001
1	0	3.082692	-2.136773	-2.687544
1	0	4.606911	-1.193836	-2.710971
8	0	-1.234870	-1.798881	1.091757

Furan + Maleimide (HB: furan -O- + glycol, conformation 1): Exo

- Reactant 1 + glycol:

6	0	-2.138597	-0.542976	-0.656264
6	0	-2.033424	0.451749	0.268534
6	0	-4.170971	0.079745	0.031839
6	0	-3.546014	-0.782507	-0.811867
1	0	-1.317462	-1.038507	-1.168392
1	0	-4.023170	-1.502578	-1.468592
1	0	-5.210684	0.274361	0.269934
8	0	-3.262211	0.839918	0.691453
6	0	-0.880120	1.159626	0.895423
1	0	-0.482242	0.586559	1.755305
1	0	-1.228099	2.137153	1.274238
8	0	0.142306	1.330729	-0.065688
6	0	1.298156	1.935377	0.480554
1	0	1.673075	1.358679	1.346397
1	0	1.054753	2.957104	0.834415
6	0	2.364717	1.991556	-0.601803
1	0	1.959814	2.523891	-1.481240
1	0	2.613147	0.969200	-0.922238
8	0	3.550426	2.572778	-0.111531
1	0	3.383516	3.513561	0.027546
8	0	1.025875	-1.022483	-1.291016
1	0	0.636681	-0.141625	-1.163017

6	0	1.261522	-1.531272	0.003858
1	0	1.983246	-0.909830	0.568239
1	0	0.330041	-1.592443	0.599351
6	0	1.838536	-2.922307	-0.142799
1	0	1.114961	-3.551932	-0.691980
1	0	2.760754	-2.860396	-0.748602
8	0	2.085157	-3.404250	1.161412
1	0	2.456417	-4.290672	1.086742

- Reactant complex:

6	0	1.616156	-2.310655	-0.293531
6	0	0.918499	-1.544147	0.592324
6	0	-0.357634	-3.261934	0.147659
6	0	0.776033	-3.434523	-0.585994
1	0	2.590104	-2.072890	-0.714991
1	0	0.988280	-4.262057	-1.255394
6	0	-0.795879	0.103596	-1.612821
1	0	0.046348	0.520020	-2.158977
6	0	-1.465160	-1.033065	-1.813087
1	0	-1.321165	-1.800168	-2.569523
1	0	-1.276678	-3.827641	0.258347
8	0	-0.281035	-2.116666	0.870423
6	0	-2.525857	-1.153458	-0.753956
6	0	-1.399914	0.805966	-0.427062
7	0	-2.403438	-0.027464	0.053430
8	0	-1.092935	1.875401	0.045859
8	0	-3.332268	-2.041887	-0.607145
6	0	1.199429	-0.231029	1.238669
1	0	2.008607	-0.312135	1.988073
1	0	0.286147	0.118875	1.753399
8	0	1.577090	0.691986	0.234262
6	0	1.817921	1.990687	0.750518
1	0	2.677830	1.980581	1.445404
1	0	0.920188	2.339491	1.291602
6	0	-3.287915	0.317701	1.159560
1	0	-2.918594	1.293440	1.507339
6	0	2.099538	2.902698	-0.430258
1	0	1.234681	2.858583	-1.115223
1	0	2.987190	2.539204	-0.970701
8	0	2.382070	4.214611	-0.001719
1	0	1.556605	4.593969	0.326480
6	0	-4.710135	0.508583	0.648250
1	0	-5.112468	-0.455456	0.283417
1	0	-5.348805	0.854515	1.485170
6	0	-3.212566	-0.716445	2.277141
1	0	-2.176568	-0.832993	2.621571
1	0	-3.833795	-0.400574	3.127287
1	0	-3.568526	-1.692269	1.919240
8	0	-4.675004	1.457888	-0.384678
6	0	-5.939397	1.660482	-0.960953
1	0	-6.666491	2.041904	-0.219985
1	0	-5.823287	2.402332	-1.760334
1	0	-6.340731	0.724499	-1.391167
8	0	3.832692	-0.099825	-1.203726
1	0	2.929395	0.144150	-0.940202
6	0	4.614806	-0.007037	-0.033622

1	0	4.623051	1.018873	0.380433
1	0	4.254292	-0.688277	0.762228
6	0	6.033475	-0.391350	-0.393282
1	0	6.031276	-1.416756	-0.806388
1	0	6.392928	0.290629	-1.184997
8	0	6.799704	-0.293235	0.788650
1	0	7.713332	-0.510206	0.571142

- Transition state:

6	0	1.472224	-2.029213	-0.533491
6	0	0.758587	-1.187173	0.363391
6	0	-0.498102	-2.902269	0.048387
6	0	0.675695	-3.135063	-0.717706
1	0	2.395525	-1.755939	-1.042951
1	0	0.818096	-3.954796	-1.416257
6	0	-0.641989	-0.436045	-1.137895
1	0	0.063110	-0.043855	-1.864435
6	0	-1.438385	-1.584570	-1.270976
1	0	-1.509265	-2.216297	-2.152516
1	0	-1.277916	-3.600738	0.344037
8	0	-0.232428	-1.908361	0.945992
6	0	-2.674324	-1.370741	-0.448779
6	0	-1.376749	0.516990	-0.256125
7	0	-2.527631	-0.140681	0.182182
8	0	-1.061190	1.637880	0.082845
8	0	-3.616510	-2.119603	-0.309043
6	0	1.259088	0.005441	1.117436
1	0	2.035569	-0.290852	1.847793
1	0	0.421444	0.471138	1.661653
8	0	1.789161	0.898601	0.166112
6	0	1.974584	2.210130	0.671943
1	0	2.724905	2.216108	1.483488
1	0	1.009942	2.580383	1.061148
6	0	-3.472781	0.435331	1.129697
1	0	-3.046664	1.419207	1.373063
6	0	2.441996	3.077967	-0.483368
1	0	1.691937	3.016061	-1.291796
1	0	3.393694	2.688137	-0.875733
8	0	2.679988	4.400108	-0.060871
1	0	1.823614	4.800380	0.137474
6	0	-4.820837	0.663087	0.458502
1	0	-5.268388	-0.306696	0.172457
1	0	-5.499696	1.159268	1.180850
6	0	-3.592777	-0.426966	2.381779
1	0	-2.602578	-0.576677	2.833668
1	0	-4.245067	0.059920	3.120582
1	0	-4.013193	-1.410174	2.129021
8	0	-4.613489	1.470387	-0.670792
6	0	-5.797318	1.678741	-1.394983
1	0	-6.561125	2.196807	-0.784972
1	0	-5.549760	2.304605	-2.261085
1	0	-6.226899	0.723701	-1.749757
8	0	3.971798	-0.071688	-1.244027
1	0	3.117474	0.325496	-1.006342
6	0	4.652993	-0.260721	-0.022505
1	0	4.701473	0.670601	0.571827

1	0	4.168103	-1.033962	0.606134
6	0	6.061930	-0.711940	-0.339739
1	0	6.010541	-1.631155	-0.951755
1	0	6.556365	0.069067	-0.945072
8	0	6.712441	-0.927391	0.894690
1	0	7.620983	-1.192367	0.711540

- Exo product + glycol:

6	0	1.369576	-2.030176	-0.593066
6	0	0.569512	-1.052886	0.264917
6	0	-0.674754	-2.774047	0.068554
6	0	0.591996	-3.110005	-0.704268
1	0	2.322969	-1.802714	-1.072440
1	0	0.754302	-4.007892	-1.297136
6	0	-0.559160	-0.499081	-0.682559
1	0	-0.157285	-0.082265	-1.610790
6	0	-1.459962	-1.739087	-0.803715
1	0	-1.665658	-2.098052	-1.817889
1	0	-1.282230	-3.587124	0.475841
8	0	-0.185220	-1.921429	1.102451
6	0	-2.756535	-1.347164	-0.115970
6	0	-1.415947	0.535440	0.027132
7	0	-2.640553	-0.041134	0.336692
8	0	-1.096372	1.664080	0.322513
8	0	-3.725985	-2.051496	0.042095
6	0	1.316652	-0.014901	1.064415
1	0	2.117691	-0.488026	1.659207
1	0	0.618462	0.496461	1.746250
8	0	1.849093	0.901479	0.129830
6	0	2.128911	2.176007	0.682295
1	0	2.846636	2.092904	1.518466
1	0	1.186256	2.615139	1.055506
6	0	-3.704213	0.691630	1.021318
1	0	-3.295058	1.703802	1.145399
6	0	2.716890	3.036590	-0.422542
1	0	2.005553	3.064303	-1.267507
1	0	3.648835	2.579745	-0.788252
8	0	3.045211	4.320551	0.054208
1	0	2.218241	4.777162	0.255569
6	0	-4.933271	0.780712	0.125380
1	0	-5.396628	-0.216299	0.011278
1	0	-5.670394	1.453897	0.606253
6	0	-4.023098	0.072003	2.376915
1	0	-3.122257	0.045226	3.004435
1	0	-4.787773	0.670037	2.892745
1	0	-4.397024	-0.953048	2.251933
8	0	-4.527138	1.286612	-1.120029
6	0	-5.596517	1.377833	-2.025776
1	0	-6.380637	2.066147	-1.659151
1	0	-5.197299	1.764688	-2.971259
1	0	-6.058011	0.389341	-2.204720
8	0	3.981975	-0.149996	-1.291153
1	0	3.149192	0.264227	-1.007708
6	0	4.712368	-0.393090	-0.108397
1	0	4.771387	0.506093	0.533260
1	0	4.264091	-1.206153	0.495727

6	0	6.114274	-0.803627	-0.503162
1	0	6.050770	-1.689201	-1.161846
1	0	6.573487	0.016520	-1.084215
8	0	6.816909	-1.074755	0.691097
1	0	7.718136	-1.324255	0.457279

- Exo product:

6	0	-1.930908	2.646535	-0.807124
6	0	-1.406060	1.554704	0.117939
6	0	0.206592	2.934949	-0.099655
6	0	-0.926037	3.515779	-0.934932
1	0	-2.898493	2.622194	-1.304541
1	0	-0.860708	4.398562	-1.567880
6	0	-0.411119	0.715961	-0.769186
1	0	-0.878078	0.368645	-1.695200
6	0	0.757124	1.706937	-0.896661
1	0	1.078025	1.959241	-1.913211
1	0	0.972959	3.606789	0.296991
8	0	-0.495568	2.268514	0.947631
6	0	1.901128	1.059975	-0.134488
6	0	0.161229	-0.462821	-0.001445
7	0	1.472116	-0.165378	0.351644
8	0	-0.412244	-1.484266	0.298423
8	0	2.998946	1.532443	0.047740
6	0	-2.403414	0.748912	0.911858
1	0	-3.087232	1.421720	1.460157
1	0	-1.863318	0.124909	1.644368
8	0	-3.089749	-0.044026	-0.023495
6	0	-3.615664	-1.234680	0.520243
1	0	-4.461214	-1.028280	1.202322
1	0	-2.824825	-1.764517	1.083405
6	0	2.314956	-1.094808	1.101502
1	0	1.681920	-1.983064	1.234073
6	0	-4.078674	-2.100913	-0.636781
1	0	-3.223280	-2.258304	-1.317675
1	0	-4.865074	-1.577256	-1.198282
8	0	-4.638842	-3.311682	-0.180063
1	0	-3.924071	-3.830264	0.211208
6	0	3.524341	-1.491640	0.263979
1	0	4.206160	-0.629825	0.144910
1	0	4.070488	-2.296592	0.794617
6	0	2.716424	-0.514690	2.452657
1	0	1.822863	-0.263549	3.039547
1	0	3.307098	-1.250345	3.016962
1	0	3.316467	0.395029	2.316109
8	0	3.061395	-1.936409	-0.984970
6	0	4.115710	-2.295477	-1.840161
1	0	4.712003	-3.127500	-1.421499
1	0	3.675149	-2.617382	-2.791627
1	0	4.793068	-1.441497	-2.024865

Furan + Maleimide (HB: furan -O- + glycol, conformation 2): Exo

- Reactant 1 + glycol:

6	0	2.138260	0.541429	-0.656834

6	0	2.033034	-0.453010	0.268260
6	0	4.170573	-0.080828	0.031922
6	0	3.545700	0.781043	-0.812241
1	0	1.317154	1.036781	-1.169186
1	0	4.022916	1.500858	-1.469207
1	0	5.210261	-0.275299	0.270264
8	0	3.261746	-0.840877	0.691626
6	0	0.879644	-1.160687	0.895172
1	0	0.482250	-0.587742	1.755381
1	0	1.227298	-2.138488	1.273607
8	0	-0.143037	-1.331069	-0.065765
6	0	-1.299220	-1.934856	0.480737
1	0	-1.673862	-1.357502	1.346278
1	0	-1.056265	-2.956498	0.835137
6	0	-2.365766	-1.991172	-0.601619
1	0	-1.960944	-2.524126	-1.480726
1	0	-2.613794	-0.968869	-0.922573
8	0	-3.551688	-2.571714	-0.111157
1	0	-3.385124	-3.512500	0.028318
8	0	-1.026370	1.022552	-1.290730
1	0	-0.637711	0.141488	-1.162558
6	0	-1.261668	1.531722	0.004057
1	0	-1.985168	0.911842	0.567886
1	0	-0.330414	1.590750	0.600117
6	0	-1.835419	2.924081	-0.142835
1	0	-1.110045	3.552075	-0.691512
1	0	-2.757404	2.864341	-0.749209
8	0	-2.081729	3.406481	1.161261
1	0	-2.451345	4.293572	1.086395

- Reactant complex:

6	0	1.927834	-2.445100	-0.134837
6	0	0.988474	-1.812817	0.624240
6	0	0.403038	-3.917444	0.579179
6	0	1.539280	-3.827173	-0.165137
1	0	2.771953	-1.972213	-0.631763
1	0	2.044690	-4.644111	-0.670455
6	0	-0.985063	-0.731310	-2.046494
1	0	-0.251552	-0.507621	-2.817055
6	0	-1.310635	-1.902238	-1.499233
1	0	-0.906744	-2.894643	-1.690623
1	0	-0.252702	-4.735451	0.856618
8	0	0.058719	-2.699142	1.060637
6	0	-2.364510	-1.660988	-0.456522
6	0	-1.811201	0.345253	-1.400907
7	0	-2.573182	-0.269895	-0.424964
8	0	-1.834891	1.529179	-1.673906
8	0	-2.953259	-2.462269	0.218942
6	0	0.758724	-0.391249	1.008867
1	0	1.471234	-0.067464	1.791674
1	0	-0.266265	-0.281879	1.403418
8	0	0.928866	0.427168	-0.139339
6	0	0.651508	1.793903	0.128042
1	0	1.267977	2.145725	0.976948
1	0	-0.409710	1.899910	0.409493
6	0	-3.630787	0.357369	0.363661

1	0	-3.954536	-0.433314	1.055608
6	0	0.906940	2.597070	-1.146663
1	0	0.789358	1.908256	-2.002514
1	0	1.935989	2.983593	-1.170696
8	0	0.036922	3.697603	-1.235368
1	0	-0.825080	3.322657	-1.472023
6	0	-3.094067	1.516529	1.191269
1	0	-2.720519	2.323899	0.533025
1	0	-3.927250	1.924473	1.794933
6	0	-4.792298	0.795210	-0.523760
1	0	-5.193428	-0.065833	-1.074948
1	0	-5.601299	1.219707	0.087285
1	0	-4.459655	1.554359	-1.245842
8	0	-2.067438	1.044507	2.028319
6	0	-1.590893	2.046632	2.895575
1	0	-2.382298	2.381456	3.589837
1	0	-0.764119	1.615695	3.474449
1	0	-1.221542	2.923343	2.332420
8	0	3.481431	0.221242	-1.207919
1	0	2.517047	0.209841	-1.086113
6	0	4.019733	0.581913	0.044137
1	0	3.610154	1.541905	0.412520
1	0	3.821494	-0.185989	0.817825
6	0	5.516836	0.729135	-0.118587
1	0	5.930969	-0.229941	-0.479760
1	0	5.714525	1.494373	-0.891028
8	0	6.035385	1.093870	1.143401
1	0	6.990228	1.193085	1.056392

- Transition state:

6	0	1.112485	-2.218537	-0.210824
6	0	0.316844	-1.186179	0.359285
6	0	-0.949120	-2.919059	0.283652
6	0	0.307719	-3.333688	-0.237136
1	0	2.103341	-2.076699	-0.640630
1	0	0.511831	-4.296030	-0.698131
6	0	-0.831408	-0.835059	-1.491071
1	0	-0.022386	-0.691432	-2.201057
6	0	-1.676954	-1.953218	-1.396598
1	0	-1.707852	-2.789781	-2.090215
1	0	-1.782771	-3.523943	0.633836
8	0	-0.767093	-1.740315	0.946847
6	0	-2.970003	-1.478675	-0.797413
6	0	-1.599074	0.353308	-1.028324
7	0	-2.822545	-0.112448	-0.554448
8	0	-1.269417	1.524503	-1.035076
8	0	-3.959416	-2.121383	-0.537397
6	0	0.730565	0.161887	0.858670
1	0	1.366192	0.067112	1.760018
1	0	-0.177606	0.729077	1.118773
8	0	1.439867	0.789897	-0.183945
6	0	1.586731	2.186480	-0.000495
1	0	2.242624	2.404274	0.862240
1	0	0.590668	2.630480	0.176102
6	0	-3.831785	0.686316	0.127051
1	0	-4.508015	-0.056424	0.573984

6	0	2.187243	2.747895	-1.277372
1	0	1.524094	2.478824	-2.118230
1	0	3.168940	2.283626	-1.457604
8	0	2.399367	4.136641	-1.171904
1	0	1.533164	4.563464	-1.185438
6	0	-3.218160	1.520357	1.246428
1	0	-2.631460	2.357870	0.827253
1	0	-4.043534	1.942260	1.852703
6	0	-4.594958	1.566094	-0.858106
1	0	-5.098623	0.944085	-1.609806
1	0	-5.357147	2.160694	-0.333467
1	0	-3.900877	2.249344	-1.368178
8	0	-2.398743	0.692974	2.034821
6	0	-1.894695	1.368474	3.158386
1	0	-2.709297	1.713673	3.821167
1	0	-1.259841	0.663152	3.709613
1	0	-1.290037	2.247999	2.864377
8	0	3.729876	-0.486263	-1.077223
1	0	2.863643	-0.048255	-1.028978
6	0	4.291049	-0.353504	0.210800
1	0	4.338119	0.702999	0.534240
1	0	3.712934	-0.911516	0.973742
6	0	5.696525	-0.911876	0.165161
1	0	5.649872	-1.964766	-0.168677
1	0	6.277945	-0.345867	-0.584900
8	0	6.231942	-0.787144	1.465780
1	0	7.134811	-1.124456	1.450599

- Exo product + glycol:

6	0	1.103991	-2.203452	-0.437979
6	0	0.191383	-1.082828	0.056147
6	0	-1.034921	-2.825367	0.027362
6	0	0.331371	-3.293156	-0.452655
1	0	2.125541	-2.066359	-0.796001
1	0	0.569564	-4.290297	-0.817423
6	0	-0.746081	-0.766421	-1.175468
1	0	-0.173382	-0.595613	-2.091627
6	0	-1.666379	-1.997257	-1.139546
1	0	-1.766945	-2.561611	-2.073310
1	0	-1.713199	-3.544187	0.495146
8	0	-0.698018	-1.769819	0.923127
6	0	-3.014015	-1.450713	-0.693862
6	0	-1.655981	0.413804	-0.892798
7	0	-2.929168	-0.060189	-0.629768
8	0	-1.342609	1.585316	-0.869235
8	0	-3.997193	-2.091186	-0.420866
6	0	0.792671	0.122030	0.732935
1	0	1.456858	-0.187983	1.559035
1	0	-0.024036	0.742869	1.137073
8	0	1.508009	0.827752	-0.259909
6	0	1.707581	2.195091	0.047964
1	0	2.279438	2.307970	0.986995
1	0	0.721973	2.681563	0.162494
6	0	-3.985477	0.755532	-0.034320
1	0	-4.744948	0.022881	0.271505
6	0	2.473947	2.816856	-1.106590

1	0	1.905746	2.647045	-2.038773
1	0	3.447444	2.315295	-1.214456
8	0	2.734797	4.180585	-0.868903
1	0	1.892653	4.650865	-0.917002
6	0	-3.452287	1.461451	1.214016
1	0	-2.862520	2.352731	0.932052
1	0	-4.315376	1.792936	1.823917
6	0	-4.569110	1.744207	-1.035252
1	0	-5.019928	1.213333	-1.884026
1	0	-5.350352	2.349785	-0.553116
1	0	-3.782232	2.414226	-1.408187
8	0	-2.654623	0.547870	1.920263
6	0	-2.157980	1.078829	3.121265
1	0	-2.975452	1.339098	3.818439
1	0	-1.525196	0.308024	3.578214
1	0	-1.552537	1.987246	2.939627
8	0	3.826272	-0.456758	-1.065865
1	0	2.963977	-0.010100	-1.017555
6	0	4.339183	-0.441053	0.248581
1	0	4.335016	0.576640	0.682322
1	0	3.759081	-1.096367	0.927489
6	0	5.765877	-0.942532	0.197093
1	0	5.769679	-1.953399	-0.250346
1	0	6.351227	-0.278653	-0.464590
8	0	6.251548	-0.942936	1.522964
1	0	7.163979	-1.253328	1.506518

- Exo product:

6	0	-0.637456	3.153084	0.095313
6	0	-0.403797	1.675605	0.391008
6	0	1.542811	2.530272	0.230860
6	0	0.582011	3.688547	-0.002360
1	0	-1.611605	3.602053	-0.088729
1	0	0.863041	4.701817	-0.282726
6	0	0.073487	1.074243	-0.989871
1	0	-0.609861	1.335107	-1.802828
6	0	1.500989	1.641053	-1.054534
1	0	1.775256	2.178248	-1.969369
1	0	2.547486	2.738062	0.609135
8	0	0.809845	1.701469	1.127817
6	0	2.400063	0.430898	-0.852683
6	0	0.251306	-0.429979	-0.914381
7	0	1.604467	-0.713297	-0.834853
8	0	-0.624544	-1.268688	-0.903803
8	0	3.597059	0.437453	-0.714160
6	0	-1.477828	0.888346	1.096466
1	0	-1.773048	1.397298	2.031957
1	0	-1.071054	-0.107164	1.346561
8	0	-2.549436	0.791780	0.192724
6	0	-3.369867	-0.334898	0.403389
1	0	-3.985606	-0.222762	1.315179
1	0	-2.739390	-1.237949	0.509330
6	0	2.133474	-2.021778	-0.456047
1	0	3.194011	-1.827662	-0.244597
6	0	-4.262202	-0.485786	-0.815001
1	0	-3.617505	-0.560779	-1.708518

1	0	-4.890990	0.408865	-0.925081
8	0	-5.134943	-1.585598	-0.680402
1	0	-4.595054	-2.385914	-0.707729
6	0	1.456003	-2.500218	0.829299
1	0	0.453391	-2.909947	0.608417
1	0	2.072921	-3.307453	1.270687
6	0	1.990105	-3.037920	-1.581647
1	0	2.551899	-2.713970	-2.467643
1	0	2.385160	-4.012749	-1.260389
1	0	0.931802	-3.156616	-1.851776
8	0	1.357777	-1.404072	1.699783
6	0	0.781447	-1.743858	2.933751
1	0	1.397337	-2.481898	3.479963
1	0	0.714008	-0.821677	3.523972
1	0	-0.232818	-2.166246	2.801090

Furan + Maleimide (HB: maleimide -O- + glycol, conformation 1): Endo

- Reactant 1 + glycol:

6	0	1.256420	-2.440228	-0.648927
6	0	1.994140	-1.672823	-1.450519
6	0	0.839056	-1.624744	0.542176
6	0	2.084009	-0.297390	-0.849997
1	0	2.466945	-1.911915	-2.399712
1	0	0.960733	-3.480219	-0.758942
7	0	1.364791	-0.343947	0.338819
8	0	2.660177	0.669004	-1.292434
8	0	0.214836	-1.971810	1.511752
6	0	1.135929	0.767143	1.253704
1	0	0.318254	0.422974	1.904732
6	0	0.676964	2.007764	0.494850
1	0	0.355651	2.773056	1.224760
1	0	1.509464	2.411424	-0.103970
6	0	2.376973	1.066319	2.088115
1	0	2.185955	1.900701	2.778254
1	0	2.656310	0.184865	2.679841
1	0	3.217940	1.335917	1.432754
8	0	-0.398066	1.658606	-0.351967
6	0	-0.669846	2.645539	-1.325970
1	0	0.211644	2.807699	-1.969245
1	0	-1.505291	2.277033	-1.934430
1	0	-0.956927	3.600209	-0.852988
8	0	-1.353640	-0.910613	-0.976965
1	0	-0.921917	-0.053452	-0.811316
6	0	-2.398685	-1.017913	-0.035868
1	0	-2.888128	-1.990725	-0.213136
1	0	-2.023236	-1.027414	1.003751
6	0	-3.418414	0.102507	-0.193159
1	0	-2.919376	1.064591	0.000955
1	0	-3.781750	0.117768	-1.238352
8	0	-4.470119	0.003670	0.742170
1	0	-4.924586	-0.832780	0.578435

- Reactant complex:

6	0	1.759056	-3.437736	0.234823

6	0	2.418536	-3.276436	-0.945737
6	0	3.004945	-1.579239	0.291883
6	0	2.146829	-2.322642	1.047262
6	0	-0.027659	-1.257300	-1.547360
6	0	0.725843	-0.192736	-1.268568
1	0	2.449514	-3.851348	-1.865022
1	0	1.070263	-4.237984	0.486181
1	0	1.826812	-2.092098	2.058418
8	0	3.184561	-2.161532	-0.919691
6	0	-0.924283	-1.529874	-0.377577
6	0	0.347958	0.315660	0.093918
1	0	1.513805	0.275815	-1.849896
1	0	-0.046074	-1.880957	-2.436786
7	0	-0.655391	-0.524564	0.561650
8	0	0.799880	1.270367	0.691691
8	0	-1.708676	-2.429888	-0.211493
6	0	-1.425599	-0.372244	1.788338
1	0	-2.343663	-0.953284	1.612039
6	0	3.737295	-0.313676	0.567490
1	0	3.414874	0.045784	1.561086
1	0	4.829381	-0.491694	0.603674
8	0	3.437005	0.635236	-0.428757
6	0	3.752828	1.951049	-0.038229
1	0	3.290266	2.189368	0.935874
1	0	4.852193	2.076192	0.055450
6	0	3.204430	2.891675	-1.096335
1	0	3.564755	2.569803	-2.090858
1	0	2.108274	2.818210	-1.087357
8	0	3.528693	4.234368	-0.812616
1	0	4.484445	4.331305	-0.910186
6	0	-1.800553	1.088155	2.021983
1	0	-2.534725	1.138401	2.846551
1	0	-0.905945	1.665790	2.302336
6	0	-0.687379	-0.942958	2.994039
1	0	-1.283476	-0.815682	3.909450
1	0	-0.503722	-2.016014	2.848355
1	0	0.275483	-0.426699	3.127517
8	0	-2.359078	1.615535	0.837318
6	0	-2.318142	3.027692	0.790017
1	0	-1.274574	3.382517	0.830955
1	0	-2.773652	3.330991	-0.160894
1	0	-2.890516	3.468997	1.623739
8	0	-2.722654	0.218121	-1.580104
1	0	-2.478272	0.722727	-0.784192
6	0	-3.952221	-0.406681	-1.290535
1	0	-4.221816	-1.006513	-2.176410
1	0	-3.875195	-1.108437	-0.439166
6	0	-5.049679	0.610616	-1.006516
1	0	-4.767422	1.196591	-0.118241
1	0	-5.123879	1.311445	-1.859372
8	0	-6.283697	-0.004689	-0.704583
1	0	-6.550880	-0.511379	-1.482284

- Transition state:

6	0	0.983253	-2.869461	-1.157030
6	0	1.320680	-1.914428	-2.149955

6	0	2.433145	-1.346725	-0.402625
6	0	1.689956	-2.501172	-0.034427
6	0	0.123357	-0.297236	-1.471630
6	0	0.864813	0.092661	-0.350972
1	0	1.148920	-1.963681	-3.221581
1	0	0.203457	-3.620683	-1.248332
1	0	1.599628	-2.893295	0.975437
8	0	2.455912	-1.268372	-1.762616
6	0	-1.136215	-0.931458	-0.979988
6	0	0.101070	-0.296109	0.865079
1	0	1.575396	0.913661	-0.307754
1	0	0.111847	0.217421	-2.428685
7	0	-1.052790	-0.941534	0.421275
8	0	0.403831	-0.141852	2.031514
8	0	-2.052371	-1.408242	-1.607668
6	0	-2.070774	-1.539004	1.272740
1	0	-2.891241	-1.790176	0.584428
6	0	3.576020	-0.742741	0.347152
1	0	3.295947	-0.716014	1.417081
1	0	4.471813	-1.385399	0.237623
8	0	3.841836	0.543410	-0.145699
6	0	4.938579	1.148664	0.497902
1	0	4.774675	1.215957	1.589331
1	0	5.858611	0.550408	0.333154
6	0	5.114835	2.540636	-0.083993
1	0	5.209399	2.462904	-1.181612
1	0	4.217992	3.138039	0.129790
8	0	6.202617	3.209491	0.511797
1	0	7.013070	2.773000	0.220273
6	0	-2.578821	-0.528998	2.296333
1	0	-3.440305	-0.964918	2.835294
1	0	-1.780824	-0.298589	3.021036
6	0	-1.551908	-2.797798	1.961125
1	0	-2.334044	-3.245730	2.591087
1	0	-1.245464	-3.541007	1.212275
1	0	-0.687588	-2.550546	2.594913
8	0	-2.970706	0.647188	1.620732
6	0	-3.138033	1.747419	2.490540
1	0	-2.202160	1.958149	3.035746
1	0	-3.400881	2.612350	1.868686
1	0	-3.947334	1.559023	3.216974
8	0	-2.247088	1.670869	-0.904178
1	0	-2.386226	1.222893	-0.050944
6	0	-3.427601	1.494537	-1.655106
1	0	-3.276215	2.022464	-2.612672
1	0	-3.615208	0.431517	-1.887983
6	0	-4.643626	2.077520	-0.946452
1	0	-4.800590	1.532254	-0.002801
1	0	-4.441429	3.136452	-0.693439
8	0	-5.827924	1.922682	-1.698476
1	0	-5.702953	2.395040	-2.531664

- Endo product + glycol:

6	0	0.936034	-3.066462	-1.083516
6	0	0.991756	-1.912868	-2.076401
6	0	2.128270	-1.251525	-0.392482

6	0	1.647358	-2.655427	-0.030238
6	0	0.066855	-0.769749	-1.524390
6	0	0.877448	-0.304082	-0.312872
1	0	0.870446	-2.141712	-3.137789
1	0	0.342655	-3.972617	-1.192297
1	0	1.774546	-3.139514	0.937177
8	0	2.272407	-1.350428	-1.808848
6	0	-1.262784	-1.214672	-0.955909
6	0	-0.004954	-0.556094	0.887254
1	0	1.202285	0.740719	-0.352662
1	0	-0.097490	0.009236	-2.274446
7	0	-1.217707	-1.057043	0.434211
8	0	0.285379	-0.395864	2.053565
8	0	-2.212835	-1.664026	-1.549774
6	0	-2.338960	-1.437357	1.287557
1	0	-3.187252	-1.538132	0.595232
6	0	3.371100	-0.750734	0.291036
1	0	3.214666	-0.812233	1.385181
1	0	4.219934	-1.404743	0.015801
8	0	3.610222	0.573914	-0.104913
6	0	4.760241	1.119262	0.492191
1	0	4.688807	1.095734	1.596003
1	0	5.659390	0.536050	0.203517
6	0	4.900070	2.554873	0.016664
1	0	4.910383	2.568707	-1.087620
1	0	4.024463	3.130559	0.346507
8	0	6.033486	3.175446	0.580196
1	0	6.816423	2.756995	0.200381
6	0	-2.648990	-0.339657	2.299684
1	0	-3.614510	-0.568498	2.786620
1	0	-1.859881	-0.298710	3.066839
6	0	-2.067468	-2.768406	1.980808
1	0	-2.918270	-3.053781	2.616275
1	0	-1.913931	-3.557825	1.232781
1	0	-1.167557	-2.690135	2.608039
8	0	-2.725338	0.899576	1.624194
6	0	-2.653789	2.003979	2.503638
1	0	-1.705083	1.989115	3.066359
1	0	-2.700500	2.910732	1.887478
1	0	-3.499558	2.000610	3.212213
8	0	-1.734091	1.590313	-0.918888
1	0	-1.998155	1.288044	-0.030912
6	0	-2.911840	1.619734	-1.698847
1	0	-2.623061	1.987279	-2.698295
1	0	-3.348472	0.613012	-1.829745
6	0	-3.958707	2.548333	-1.097457
1	0	-4.245330	2.162039	-0.106841
1	0	-3.511063	3.549905	-0.950840
8	0	-5.140495	2.592447	-1.865888
1	0	-4.909665	2.952562	-2.731885

- Endo product:

6	0	-0.258624	-2.934956	0.391465
6	0	0.252951	-2.581647	-0.998094
6	0	1.267808	-1.244733	0.322689
6	0	0.374344	-2.098589	1.218631

6	0	-0.403420	-1.208567	-1.400288
6	0	0.327758	-0.252729	-0.455169
1	0	0.227339	-3.354788	-1.769444
1	0	-1.055776	-3.641326	0.615671
1	0	0.219393	-1.949082	2.286343
8	0	1.583589	-2.155815	-0.729412
6	0	-1.871200	-1.066942	-1.045910
6	0	-0.752509	0.371287	0.399403
1	0	0.944044	0.510416	-0.941628
1	0	-0.246521	-1.007388	-2.465626
7	0	-1.977218	-0.133299	-0.014414
8	0	-0.594191	1.142143	1.320109
8	0	-2.801910	-1.666822	-1.523862
6	0	-3.261534	0.217644	0.586860
1	0	-4.003173	-0.273335	-0.058545
6	0	2.486328	-0.625394	0.950585
1	0	2.159821	-0.018692	1.816905
1	0	3.152917	-1.431318	1.310942
8	0	3.136389	0.173939	-0.002210
6	0	4.293227	0.790937	0.506683
1	0	4.057849	1.422955	1.383524
1	0	5.026853	0.025182	0.833911
6	0	4.894211	1.644674	-0.596340
1	0	5.067985	1.013256	-1.485730
1	0	4.175343	2.425640	-0.879827
8	0	6.061170	2.303065	-0.158918
1	0	6.743947	1.633399	-0.025751
6	0	-3.484417	1.722937	0.515080
1	0	-4.519449	1.939442	0.846853
1	0	-2.785904	2.246559	1.192387
6	0	-3.362968	-0.311808	2.013013
1	0	-4.346755	-0.069026	2.439623
1	0	-3.243771	-1.404066	2.021066
1	0	-2.582608	0.137135	2.643185
8	0	-3.287285	2.129351	-0.814122
6	0	-3.426464	3.518340	-0.965660
1	0	-2.696142	4.066205	-0.342326
1	0	-3.247283	3.755553	-2.021411
1	0	-4.442621	3.856762	-0.689593

Furan + Maleimide (HB: maleimide -O- + glycol, conformation 2): Endo

- Reactant 1 + glycol:

6	0	0.808692	-2.269974	-1.390899
6	0	-0.263359	-2.362160	-0.604504
6	0	1.716909	-1.208030	-0.838323
6	0	-0.111188	-1.389003	0.530801
1	0	-1.136262	-3.003824	-0.693818
1	0	1.050324	-2.811061	-2.302104
7	0	1.095406	-0.720562	0.308213
8	0	-0.836483	-1.220685	1.479418
8	0	2.777763	-0.831196	-1.277092
6	0	1.615747	0.328819	1.175528
1	0	0.814876	0.498842	1.910212
6	0	1.830466	1.617182	0.391113
1	0	2.143845	2.412144	1.093072

1	0	2.624308	1.478166	-0.363588
6	0	2.895765	-0.110803	1.879288
1	0	3.252522	0.680234	2.554089
1	0	2.708162	-1.014019	2.474555
1	0	3.680643	-0.329059	1.141776
8	0	0.617151	1.976089	-0.234524
6	0	0.720095	3.167042	-0.985161
1	0	1.507114	3.083840	-1.754260
1	0	-0.249447	3.327122	-1.472668
1	0	0.947245	4.026237	-0.330772
8	0	-1.452791	0.341759	-1.114716
1	0	-0.634233	0.837462	-0.942774
6	0	-2.407454	0.813169	-0.187818
1	0	-1.974247	0.887279	0.828215
1	0	-2.795938	1.811482	-0.463712
6	0	-3.558260	-0.176273	-0.158320
1	0	-4.018259	-0.231991	-1.155506
1	0	-3.156385	-1.175831	0.087557
8	0	-4.570662	0.225761	0.738695
1	0	-4.196417	0.173283	1.627763

- Reactant complex:

6	0	0.081114	-2.090507	-1.991716
6	0	-1.158535	-2.604437	-2.227969
6	0	-1.449779	-0.465115	-1.928601
6	0	-0.110309	-0.683760	-1.798278
6	0	-1.329600	-2.556816	0.969114
6	0	-1.968791	-1.386600	0.947433
1	0	-1.522921	-3.606050	-2.430609
1	0	1.011937	-2.645853	-1.942486
1	0	0.636730	0.077413	-1.584911
8	0	-2.096082	-1.629445	-2.201022
6	0	0.129718	-2.317061	1.238460
6	0	-0.959224	-0.304460	1.198744
1	0	-3.011424	-1.159545	0.747585
1	0	-1.716071	-3.562356	0.823079
7	0	0.275899	-0.937185	1.372478
8	0	-1.124973	0.891122	1.267068
8	0	0.995078	-3.155427	1.337102
6	0	1.482273	-0.143726	1.602105
1	0	1.397660	0.716136	0.917757
6	0	-2.260718	0.776625	-1.823687
1	0	-1.562519	1.603175	-1.605548
1	0	-2.771434	0.995674	-2.780437
8	0	-3.213578	0.644403	-0.790088
6	0	-3.678438	1.886139	-0.316184
1	0	-2.831663	2.544626	-0.053818
1	0	-4.291071	2.395511	-1.089657
6	0	-4.513228	1.623702	0.924599
1	0	-5.275423	0.856372	0.693293
1	0	-3.853008	1.226414	1.708236
8	0	-5.079426	2.811264	1.431019
1	0	-5.720718	3.128642	0.782535
6	0	2.744386	-0.913194	1.250015
1	0	3.613829	-0.254171	1.436917
1	0	2.839146	-1.809971	1.879826

6	0	1.535918	0.355502	3.042915
1	0	2.403953	1.015053	3.188782
1	0	0.626612	0.925403	3.272064
1	0	1.614731	-0.489952	3.742747
8	0	2.702278	-1.285274	-0.115394
6	0	3.566057	-2.367560	-0.405214
1	0	3.232406	-3.265374	0.138911
1	0	3.513831	-2.545851	-1.486464
1	0	4.608883	-2.129918	-0.131234
8	0	2.543971	1.230709	-1.244675
1	0	2.701078	0.303082	-0.989601
6	0	3.428523	2.055368	-0.524802
1	0	3.145775	2.117624	0.549524
1	0	4.471501	1.693049	-0.569742
6	0	3.364732	3.449045	-1.125959
1	0	3.708554	3.405559	-2.168675
1	0	2.311652	3.783920	-1.138674
8	0	4.212553	4.351138	-0.450585
1	0	3.843516	4.494883	0.429998

- Transition state:

6	0	0.151741	-0.807899	-2.019974
6	0	-0.921670	-1.734293	-2.003623
6	0	-1.701210	0.071106	-1.143111
6	0	-0.343827	0.349783	-1.465458
6	0	-0.879789	-2.248098	0.064969
6	0	-1.396979	-1.060740	0.602111
1	0	-1.010846	-2.683099	-2.525777
1	0	1.185985	-1.040573	-2.256387
1	0	0.223013	1.230935	-1.170187
8	0	-2.084739	-1.051931	-1.818288
6	0	0.567031	-2.325557	0.426692
6	0	-0.275576	-0.352780	1.277977
1	0	-2.420387	-0.899190	0.930955
1	0	-1.433966	-3.173947	-0.069383
7	0	0.877190	-1.120570	1.064419
8	0	-0.286536	0.694269	1.885587
8	0	1.339990	-3.235595	0.215209
6	0	2.168105	-0.660062	1.566571
1	0	2.163753	0.427513	1.394502
6	0	-2.760130	1.073919	-0.817192
1	0	-2.318271	1.810622	-0.120456
1	0	-3.061005	1.606220	-1.740720
8	0	-3.869950	0.429113	-0.248349
6	0	-4.920278	1.322128	0.038218
1	0	-4.590846	2.119261	0.730067
1	0	-5.273075	1.811568	-0.893163
6	0	-6.052743	0.528572	0.666576
1	0	-6.325970	-0.303408	-0.006568
1	0	-5.705397	0.089480	1.611897
8	0	-7.147300	1.358431	0.980686
1	0	-7.543340	1.651224	0.150118
6	0	3.326258	-1.282629	0.801029
1	0	4.266662	-0.811320	1.143546
1	0	3.374387	-2.363481	0.998091
6	0	2.300731	-0.936272	3.061534

1	0	3.256491	-0.548104	3.443295
1	0	1.484510	-0.439650	3.601465
1	0	2.255757	-2.018013	3.257354
8	0	3.151158	-1.062023	-0.586517
6	0	3.870600	-1.986776	-1.377163
1	0	3.496490	-3.006184	-1.189576
1	0	3.705510	-1.717913	-2.428398
1	0	4.952044	-1.938035	-1.161706
8	0	2.384970	1.574865	-0.827154
1	0	2.600533	0.625925	-0.884417
6	0	3.539957	2.243531	-0.378440
1	0	3.802205	1.945073	0.659843
1	0	4.419192	2.033326	-1.013679
6	0	3.257889	3.735562	-0.403445
1	0	3.041441	4.041175	-1.436762
1	0	2.354207	3.939691	0.199151
8	0	4.372271	4.488598	0.021378
1	0	4.501006	4.312114	0.961921

- Endo product + glycol:

6	0	0.248058	-1.671696	-1.911927
6	0	-0.604351	-2.620478	-1.085598
6	0	-1.555612	-0.716654	-0.908624
6	0	-0.342738	-0.478841	-1.798495
6	0	-0.327629	-2.285584	0.428654
6	0	-1.028280	-0.931232	0.559920
1	0	-0.594473	-3.684177	-1.335074
1	0	1.202105	-1.913383	-2.376171
1	0	0.028682	0.493436	-2.117405
8	0	-1.905279	-2.064061	-1.228785
6	0	1.122668	-2.022409	0.786311
6	0	0.044665	0.030067	1.016348
1	0	-1.886635	-0.911141	1.239633
1	0	-0.735038	-3.071756	1.074557
7	0	1.250886	-0.673636	1.098214
8	0	-0.075308	1.199229	1.291643
8	0	2.021607	-2.833205	0.805303
6	0	2.477788	0.007742	1.503062
1	0	2.309586	1.055158	1.216078
6	0	-2.709611	0.238018	-1.046155
1	0	-2.331686	1.266016	-0.886591
1	0	-3.112030	0.164445	-2.073808
8	0	-3.693617	-0.086355	-0.098551
6	0	-4.813606	0.760845	-0.170198
1	0	-4.526886	1.818538	-0.019451
1	0	-5.292510	0.683814	-1.168490
6	0	-5.794896	0.334401	0.907603
1	0	-6.023387	-0.738882	0.781104
1	0	-5.325144	0.459463	1.892873
8	0	-6.950352	1.141611	0.901323
1	0	-7.433773	0.955658	0.086343
6	0	3.699298	-0.532006	0.767066
1	0	4.549890	0.149807	0.956617
1	0	3.954843	-1.533083	1.144282
6	0	2.671872	-0.082288	3.013426
1	0	3.586931	0.447175	3.316573

1	0	1.819510	0.378538	3.529793
1	0	2.755140	-1.133451	3.326903
8	0	3.425886	-0.601637	-0.617370
6	0	4.256434	-1.525223	-1.289986
1	0	4.078162	-2.541849	-0.902221
1	0	3.996086	-1.483595	-2.354817
1	0	5.322471	-1.264467	-1.171558
8	0	1.799647	1.539648	-1.255784
1	0	2.402857	0.776553	-1.212222
6	0	2.509879	2.712320	-0.936984
1	0	3.194719	2.552498	-0.077171
1	0	3.126691	3.073818	-1.780020
6	0	1.491844	3.777512	-0.564338
1	0	0.828515	3.948710	-1.424779
1	0	0.869409	3.398916	0.265630
8	0	2.107898	5.014825	-0.274202
1	0	2.561208	4.921282	0.572758

- Endo product:

6	0	0.864046	-2.151478	0.234935
6	0	0.121596	-1.813016	1.518031
6	0	-1.054080	-0.957226	-0.045734
6	0	0.133261	-1.611793	-0.743357
6	0	0.350332	-0.274387	1.777176
6	0	-0.504068	0.335533	0.663989
1	0	0.261838	-2.457892	2.388644
1	0	1.853680	-2.599758	0.170932
1	0	0.393351	-1.495715	-1.794168
8	0	-1.234502	-1.799662	1.091312
6	0	1.761769	0.208374	1.499057
6	0	0.455417	1.132730	-0.189957
1	0	-1.351649	0.943673	0.996489
1	0	0.041854	-0.004432	2.793214
7	0	1.727953	1.030588	0.377998
8	0	0.202175	1.737179	-1.202982
8	0	2.763803	-0.080753	2.112581
6	0	2.918205	1.555950	-0.289726
1	0	2.522562	2.079831	-1.171212
6	0	-2.313800	-0.769316	-0.845377
1	0	-2.064248	-0.191565	-1.755794
1	0	-2.697233	-1.761919	-1.147594
8	0	-3.257144	-0.085407	-0.062381
6	0	-4.470632	0.127384	-0.740327
1	0	-4.314566	0.705699	-1.670310
1	0	-4.929709	-0.842656	-1.023389
6	0	-5.401096	0.887454	0.188714
1	0	-5.498770	0.328193	1.136202
1	0	-4.956475	1.864019	0.424659
8	0	-6.644554	1.142588	-0.424035
1	0	-7.099319	0.297201	-0.528547
6	0	3.795810	0.409929	-0.776536
1	0	4.650721	0.834568	-1.338675
1	0	4.195372	-0.156687	0.084873
6	0	3.685412	2.520530	0.607448
1	0	4.538238	2.944824	0.058921
1	0	3.033572	3.346637	0.921295

1	0	4.057470	2.003185	1.501718
8	0	3.021499	-0.424028	-1.603190
6	0	3.758888	-1.519161	-2.080976
1	0	4.159927	-2.129844	-1.250349
1	0	3.083571	-2.136581	-2.687092
1	0	4.607568	-1.193276	-2.710406

Furan + Maleimide (HB: maleimide -O- + glycol, conformation 1): Exo

- Reactant 1 + glycol:

6	0	0.878279	-2.251732	-1.395597
1	0	1.128383	-2.778388	-2.312993
6	0	-0.183257	-2.382416	-0.600518
1	0	-1.037305	-3.049248	-0.686812
6	0	-0.050989	-1.413440	0.541068
6	0	1.758141	-1.165297	-0.843768
7	0	1.132488	-0.705288	0.310622
8	0	2.802763	-0.753280	-1.290541
8	0	-0.770056	-1.275519	1.498008
6	0	1.621249	0.360527	1.175934
1	0	0.817089	0.504960	1.912507
6	0	2.916673	-0.037063	1.876186
1	0	3.704434	-0.235296	1.135982
1	0	3.252655	0.767707	2.545479
6	0	1.792617	1.654192	0.389182
1	0	2.067055	2.463092	1.091540
1	0	2.598528	1.544519	-0.357592
8	0	-1.473058	0.264394	-1.075393
1	0	-0.660283	0.774861	-0.921021
6	0	-2.430864	0.761293	-0.163352
1	0	-2.851097	1.729088	-0.496048
1	0	-2.000980	0.902469	0.843612
6	0	-3.556141	-0.246987	-0.080321
1	0	-3.899151	-0.475275	-1.107067
1	0	-3.162672	-1.175103	0.371564
8	0	-4.578711	0.333734	0.704020
1	0	-5.250057	-0.339508	0.861296
8	0	0.572390	1.961586	-0.249601
6	0	0.632810	3.154141	-1.001439
1	0	-0.339193	3.274749	-1.495365
1	0	0.820921	4.022947	-0.347283
1	0	1.427746	3.101210	-1.765186
1	0	2.758774	-0.942388	2.476836

- Reactant complex:

6	0	-2.597275	3.141041	-0.163090
6	0	-2.456892	2.027422	0.608429
6	0	-0.693614	3.264734	0.995568
6	0	-1.441871	3.950163	0.088087
1	0	-3.421585	3.343517	-0.839416
1	0	-1.198724	4.914505	-0.346591
6	0	-0.547709	0.326354	-1.315589
1	0	-1.291962	0.273703	-2.104455
6	0	0.429905	1.212528	-1.117937
1	0	0.709511	2.079093	-1.710925

1	0	0.258568	3.452106	1.480196
8	0	-1.302224	2.096789	1.321818
6	0	1.140945	0.864566	0.156381
6	0	-0.493691	-0.691359	-0.211978
7	0	0.553479	-0.318513	0.618292
8	0	-1.198743	-1.665703	-0.048110
8	0	2.005414	1.477243	0.734028
6	0	-3.272334	0.788391	0.753256
1	0	-4.263521	1.017141	1.188656
1	0	-2.749572	0.099842	1.443284
8	0	-3.430045	0.199518	-0.517165
6	0	-4.126939	-1.025093	-0.447857
1	0	-5.188124	-0.864235	-0.178606
1	0	-3.665571	-1.678167	0.317642
6	0	0.924668	-0.967056	1.867914
1	0	1.798755	-0.399880	2.220228
6	0	-4.027228	-1.695496	-1.805677
1	0	-2.959197	-1.832059	-2.047952
1	0	-4.476483	-1.045258	-2.569765
8	0	-4.742876	-2.911114	-1.828001
1	0	-4.263557	-3.534905	-1.267759
6	0	-0.201696	-0.889407	2.894202
1	0	-1.070982	-1.468455	2.550015
1	0	0.130819	-1.292297	3.861450
6	0	1.357045	-2.405305	1.616291
1	0	1.693146	-2.850462	2.571421
1	0	0.508516	-2.996958	1.229203
8	0	2.870764	-0.583827	-1.386310
1	0	2.556368	-1.255108	-0.757749
6	0	4.110936	-0.125457	-0.889447
1	0	4.927436	-0.842849	-1.096620
1	0	4.075155	0.048479	0.200045
6	0	4.431494	1.179278	-1.585521
1	0	4.345689	1.024408	-2.677477
1	0	3.683328	1.932549	-1.279837
8	0	5.741967	1.546435	-1.202540
1	0	5.920362	2.423020	-1.560872
8	0	2.417156	-2.401299	0.684776
6	0	2.895972	-3.695783	0.393777
1	0	3.674057	-3.589063	-0.372002
1	0	3.329990	-4.166686	1.292805
1	0	2.087936	-4.340545	0.006716
1	0	-0.506323	0.156688	3.031487

- Transition state:

6	0	2.057090	-3.072223	-0.384412
6	0	2.008141	-1.938173	0.465101
6	0	0.177801	-3.013619	0.813836
6	0	0.899923	-3.776197	-0.142330
1	0	2.817249	-3.242192	-1.141188
1	0	0.517824	-4.644710	-0.671452
6	0	0.563773	-0.843718	-0.814807
1	0	1.100013	-0.874032	-1.757907
6	0	-0.585006	-1.570795	-0.471637
1	0	-1.169471	-2.189189	-1.147419
1	0	-0.669850	-3.306071	1.429430

8	0	1.044828	-2.129678	1.397181
6	0	-1.316606	-0.785323	0.575285
6	0	0.544654	0.423037	-0.039941
7	0	-0.559020	0.365080	0.811176
8	0	1.321989	1.359543	-0.080851
8	0	-2.323400	-1.079559	1.176728
6	0	3.103115	-0.969115	0.780559
1	0	3.895734	-1.462774	1.375693
1	0	2.690047	-0.139519	1.380387
8	0	3.595784	-0.517845	-0.450662
6	0	4.341500	0.676896	-0.352919
1	0	5.350168	0.490251	0.059880
1	0	3.812461	1.390926	0.305113
6	0	-0.880293	1.334987	1.847460
1	0	-1.826133	0.972110	2.275517
6	0	4.442817	1.264827	-1.748671
1	0	3.419123	1.418899	-2.131391
1	0	4.953323	0.552508	-2.412126
8	0	5.209436	2.448888	-1.750292
1	0	4.693860	3.125282	-1.292839
6	0	0.204294	1.381467	2.919542
1	0	1.147994	1.748104	2.490678
1	0	-0.092692	2.047791	3.741983
6	0	-1.127347	2.710369	1.240585
1	0	-1.459417	3.399556	2.039773
1	0	-0.197404	3.103279	0.794004
8	0	-2.657927	0.468258	-1.486502
1	0	-2.290743	1.195127	-0.956418
6	0	-3.996467	0.307213	-1.063336
1	0	-4.675873	1.013822	-1.577051
1	0	-4.098969	0.459350	0.024410
6	0	-4.429054	-1.101759	-1.408498
1	0	-4.179874	-1.293421	-2.469537
1	0	-3.858909	-1.808040	-0.779089
8	0	-5.820646	-1.183100	-1.168641
1	0	-6.084352	-2.104704	-1.266670
8	0	-2.131146	2.593102	0.255516
6	0	-2.438042	3.824065	-0.360418
1	0	-3.178509	3.619503	-1.143524
1	0	-2.864359	4.534584	0.369034
1	0	-1.539467	4.275110	-0.816168
1	0	0.368254	0.374371	3.326438

- Exo product + glycol:

6	0	1.766482	-3.085632	-0.470680
6	0	1.711024	-1.799769	0.346013
6	0	-0.097777	-2.837005	0.800706
6	0	0.641233	-3.740406	-0.176877
1	0	2.541637	-3.323208	-1.196522
1	0	0.256470	-4.664548	-0.603590
6	0	0.652222	-0.896095	-0.388949
1	0	0.875894	-0.767122	-1.451696
6	0	-0.647063	-1.640249	-0.038133
1	0	-1.273857	-1.927530	-0.887098
1	0	-0.813722	-3.283599	1.496503
8	0	0.973987	-2.199402	1.497071

6	0	-1.414857	-0.681309	0.848893
6	0	0.522439	0.458592	0.282671
7	0	-0.673199	0.495220	0.979289
8	0	1.325757	1.368351	0.254051
8	0	-2.468312	-0.883054	1.401969
6	0	3.013494	-1.128000	0.700866
1	0	3.700006	-1.854693	1.171753
1	0	2.819285	-0.313563	1.419670
8	0	3.533171	-0.628351	-0.505773
6	0	4.401639	0.468060	-0.332009
1	0	5.332142	0.168795	0.185479
1	0	3.895995	1.247353	0.269371
6	0	-1.119160	1.611729	1.808592
1	0	-2.137718	1.332257	2.113349
6	0	4.727645	1.018751	-1.708117
1	0	3.780631	1.272562	-2.217249
1	0	5.232419	0.245794	-2.304200
8	0	5.611427	2.114712	-1.627384
1	0	5.139750	2.834343	-1.188724
6	0	-0.226147	1.774321	3.034450
1	0	0.800568	2.019433	2.729005
1	0	-0.606799	2.578522	3.680079
6	0	-1.192725	2.898123	0.996032
1	0	-1.656496	3.682506	1.622746
1	0	-0.181980	3.227692	0.701767
8	0	-2.379274	0.297094	-1.554305
1	0	-2.063855	1.113563	-1.130827
6	0	-3.769213	0.234689	-1.296161
1	0	-4.343156	0.855089	-2.009583
1	0	-4.005785	0.577140	-0.274305
6	0	-4.216357	-1.203341	-1.451237
1	0	-3.839683	-1.584544	-2.419875
1	0	-3.770067	-1.802682	-0.637559
8	0	-5.628638	-1.205842	-1.394869
1	0	-5.922348	-2.122291	-1.344472
8	0	-1.983275	2.666511	-0.150913
6	0	-2.122361	3.815376	-0.958422
1	0	-2.704134	3.523158	-1.841323
1	0	-2.657192	4.614459	-0.416712
1	0	-1.137185	4.196110	-1.278631
1	0	-0.210284	0.841296	3.613005

- Exo product:

6	0	-1.994775	2.649862	-0.774427
6	0	-1.377013	1.591027	0.131508
6	0	0.116858	3.100393	-0.073758
6	0	-1.064064	3.599074	-0.894784
1	0	-2.960230	2.554235	-1.267395
1	0	-1.074174	4.493050	-1.515196
6	0	-0.323584	0.848307	-0.774170
1	0	-0.767369	0.481224	-1.703920
6	0	0.761389	1.931440	-0.888697
1	0	1.060477	2.221396	-1.901785
1	0	0.828472	3.826379	0.329292
8	0	-0.522823	2.364029	0.967212
6	0	1.952750	1.369518	-0.132009

6	0	0.344781	-0.294730	-0.029854
7	0	1.619383	0.099234	0.334167
8	0	-0.145996	-1.372030	0.236541
8	0	3.002582	1.923428	0.073558
6	0	-2.300181	0.695072	0.918348
1	0	-3.031844	1.301279	1.482225
1	0	-1.705691	0.105375	1.637486
8	0	-2.927254	-0.135908	-0.025623
6	0	-3.354897	-1.371959	0.501515
1	0	-4.217139	-1.244984	1.182091
1	0	-2.524931	-1.841551	1.062242
6	0	2.566563	-0.731453	1.074959
1	0	3.480196	-0.122170	1.114794
6	0	-3.739410	-2.259983	-0.667724
1	0	-2.869354	-2.340501	-1.343547
1	0	-4.561494	-1.794948	-1.229594
8	0	-4.203993	-3.516700	-0.228117
1	0	-3.455412	-3.976454	0.173268
6	0	2.063374	-1.030641	2.482123
1	0	1.134920	-1.616302	2.442511
1	0	2.820093	-1.601842	3.038542
6	0	2.873558	-2.000063	0.289228
1	0	3.690510	-2.543566	0.804084
1	0	1.984604	-2.656487	0.259961
8	0	3.258171	-1.625216	-1.007981
6	0	3.542968	-2.737591	-1.815935
1	0	3.821378	-2.363216	-2.808528
1	0	4.382462	-3.330781	-1.407960
1	0	2.663745	-3.400971	-1.912645
1	0	1.869705	-0.094273	3.022250

Furan + Maleimide (HB: maleimide -O- + glycol, conformation 2): Exo

- Reactant 1 + glycol:

6	0	-0.877891	-2.251400	-1.396945
1	0	-1.128451	-2.777439	-2.314573
6	0	0.183833	-2.382835	-0.602247
1	0	1.037759	-3.049748	-0.689163
6	0	0.052101	-1.414557	0.539986
6	0	-1.757425	-1.165222	-0.844108
7	0	-1.131223	-0.705945	0.310287
8	0	-2.802322	-0.753035	-1.290077
8	0	0.771426	-1.277570	1.496876
6	0	-1.619570	0.359241	1.176584
1	0	-0.814433	0.504169	1.911994
6	0	-1.793537	1.653050	0.390676
1	0	-2.600791	1.543074	-0.354569
1	0	-2.067390	2.461270	1.094066
6	0	-2.913577	-0.039800	1.878688
1	0	-2.753914	-0.945233	2.478707
1	0	-3.249360	0.764365	2.548800
1	0	-3.702244	-0.238491	1.139549
8	0	-0.574918	1.962092	-0.250388
6	0	-0.638269	3.154967	-1.001457
1	0	-0.825891	4.023218	-0.346421

1	0	0.332511	3.276919	-1.497452
1	0	-1.434819	3.101576	-1.763481
8	0	1.472302	0.265657	-1.074095
1	0	0.659710	0.776484	-0.919889
6	0	2.430047	0.762195	-0.161772
1	0	2.000907	0.900129	0.845980
1	0	2.848200	1.731520	-0.492574
6	0	3.557067	-0.244378	-0.082118
1	0	3.165196	-1.174586	0.366901
1	0	3.900280	-0.468910	-1.109616
8	0	4.578738	0.335580	0.703903
1	0	5.252805	-0.336073	0.856262

- Reactant complex:

6	0	1.621284	3.242223	-0.228780
6	0	1.342590	2.028546	-0.784991
6	0	-0.581086	2.863341	-0.154363
6	0	0.363388	3.786183	0.186079
1	0	2.609755	3.676988	-0.118933
1	0	0.181403	4.740030	0.671305
6	0	1.238254	0.549914	1.855156
1	0	2.275171	0.866617	1.923301
6	0	0.127805	1.117607	2.333533
1	0	0.011398	2.023338	2.922542
1	0	-1.660770	2.795799	-0.071379
8	0	0.007262	1.795929	-0.749142
6	0	-1.050117	0.275301	1.934402
6	0	0.849894	-0.703907	1.119702
7	0	-0.533973	-0.770469	1.158114
8	0	1.579375	-1.530194	0.613085
8	0	-2.207505	0.404420	2.240954
6	0	2.195504	0.942752	-1.347021
1	0	2.621968	1.233385	-2.326586
1	0	1.558751	0.050880	-1.493592
8	0	3.233631	0.672637	-0.432256
6	0	4.018357	-0.428786	-0.830669
1	0	4.653094	-0.173943	-1.700496
1	0	3.362477	-1.275749	-1.109644
6	0	-1.363956	-1.845912	0.636867
1	0	-2.371863	-1.410080	0.585308
6	0	4.882858	-0.832627	0.349432
1	0	4.219482	-1.062998	1.201315
1	0	5.531097	0.008161	0.634482
8	0	5.730700	-1.910627	0.018960
1	0	5.171554	-2.689711	-0.095343
6	0	-0.927843	-2.263458	-0.764737
1	0	0.012986	-2.836968	-0.720227
1	0	-1.718872	-2.909496	-1.192211
6	0	-1.369072	-3.048490	1.575938
1	0	-1.760897	-2.755938	2.559061
1	0	-2.006409	-3.850070	1.174979
1	0	-0.348201	-3.438527	1.700234
8	0	-0.746262	-1.118396	-1.569618
6	0	-0.402866	-1.443102	-2.897686
1	0	-1.200580	-2.033944	-3.381793
1	0	-0.270580	-0.498717	-3.440122

1	0	0.539068	-2.019015	-2.928906
8	0	-2.581032	0.819888	-0.785785
1	0	-1.789319	0.364335	-1.119253
6	0	-3.715053	0.085992	-1.180527
1	0	-3.484371	-0.988599	-1.311382
1	0	-4.128495	0.447998	-2.139897
6	0	-4.769900	0.228277	-0.102540
1	0	-4.357756	-0.143624	0.853120
1	0	-4.992903	1.303004	0.031765
8	0	-5.902999	-0.501321	-0.530892
1	0	-6.584992	-0.411668	0.144353

- Transition state:

6	0	1.556012	3.074543	-0.436009
6	0	1.275317	1.705874	-0.702665
6	0	-0.616376	2.608486	-0.204486
6	0	0.347112	3.652814	-0.129424
1	0	2.553769	3.499297	-0.373351
1	0	0.156158	4.656751	0.239683
6	0	1.082804	1.125333	1.360031
1	0	2.067931	1.418336	1.709908
6	0	-0.142725	1.744239	1.667550
1	0	-0.276329	2.598159	2.326269
1	0	-1.702594	2.640613	-0.247243
8	0	-0.060507	1.571317	-0.889449
6	0	-1.194673	0.682966	1.670019
6	0	0.805376	-0.325341	1.146430
7	0	-0.573854	-0.486788	1.212665
8	0	1.594190	-1.230143	0.945572
8	0	-2.351175	0.755224	2.009170
6	0	2.154224	0.680109	-1.348189
1	0	2.362600	0.957585	-2.399829
1	0	1.621920	-0.286337	-1.338822
8	0	3.335126	0.627957	-0.593846
6	0	4.076009	-0.555075	-0.800235
1	0	4.607359	-0.534902	-1.769932
1	0	3.394666	-1.426112	-0.783595
6	0	-1.306399	-1.716264	0.959888
1	0	-2.347911	-1.390776	0.820109
6	0	5.068462	-0.676153	0.341772
1	0	4.503171	-0.668761	1.289941
1	0	5.742324	0.192250	0.338343
8	0	5.876603	-1.823375	0.198896
1	0	5.313315	-2.592209	0.354383
6	0	-0.820617	-2.400694	-0.314605
1	0	0.160637	-2.874941	-0.145621
1	0	-1.553690	-3.182880	-0.590196
6	0	-1.222400	-2.670257	2.147637
1	0	-1.652515	-2.194320	3.038609
1	0	-1.782318	-3.594706	1.943383
1	0	-0.172710	-2.927315	2.350380
8	0	-0.711862	-1.449314	-1.354267
6	0	-0.334392	-2.035295	-2.579407
1	0	-1.085135	-2.771682	-2.916596
1	0	-0.260389	-1.228790	-3.319407
1	0	0.644162	-2.539447	-2.487558

8	0	-2.587794	0.608756	-1.063677
1	0	-1.790903	0.083205	-1.246182
6	0	-3.712281	-0.215736	-1.263978
1	0	-3.459283	-1.286722	-1.150352
1	0	-4.137190	-0.088317	-2.276755
6	0	-4.768711	0.148137	-0.240942
1	0	-4.361206	-0.014384	0.772371
1	0	-4.994123	1.226737	-0.338310
8	0	-5.900181	-0.657038	-0.512303
1	0	-6.570382	-0.455987	0.150635

- Exo product + glycol:

6	0	1.649687	3.095825	-0.313430
6	0	1.321101	1.612321	-0.420339
6	0	-0.547720	2.578852	-0.043642
6	0	0.479733	3.701396	-0.094103
1	0	2.655637	3.510699	-0.330916
1	0	0.282515	4.751207	0.114558
6	0	1.030223	1.139376	1.053581
1	0	1.865415	1.341642	1.730140
6	0	-0.302124	1.859489	1.334854
1	0	-0.316021	2.556681	2.178690
1	0	-1.594591	2.777540	-0.283373
8	0	0.004009	1.629109	-0.952233
6	0	-1.311450	0.749250	1.550374
6	0	0.676836	-0.338190	1.059372
7	0	-0.689551	-0.459901	1.232148
8	0	1.440150	-1.270647	0.919481
8	0	-2.447344	0.847532	1.938515
6	0	2.236932	0.718961	-1.218561
1	0	2.425540	1.159499	-2.214496
1	0	1.742426	-0.258931	-1.350810
8	0	3.421812	0.594854	-0.473011
6	0	4.134202	-0.588817	-0.751207
1	0	4.540744	-0.583765	-1.779886
1	0	3.459920	-1.459430	-0.640022
6	0	-1.445335	-1.697777	1.088970
1	0	-2.481037	-1.362898	0.931314
6	0	5.267168	-0.692001	0.253307
1	0	4.837783	-0.652368	1.270347
1	0	5.940489	0.168930	0.139033
8	0	6.042705	-1.849625	0.036362
1	0	5.487425	-2.612136	0.243656
6	0	-0.973222	-2.478801	-0.137102
1	0	-0.030568	-3.005681	0.085607
1	0	-1.748663	-3.227126	-0.391070
6	0	-1.366524	-2.554711	2.347047
1	0	-1.790031	-2.009155	3.200435
1	0	-1.937009	-3.485357	2.213018
1	0	-0.319609	-2.809760	2.565790
8	0	-0.774546	-1.591816	-1.216483
6	0	-0.262447	-2.245784	-2.354605
1	0	-0.956995	-3.025830	-2.713612
1	0	-0.133611	-1.488083	-3.137178
1	0	0.714141	-2.711250	-2.130821
8	0	-2.529169	0.613022	-1.070557

1	0	-1.713072	0.129426	-1.277330
6	0	-3.629906	-0.221040	-1.341003
1	0	-3.402221	-1.284510	-1.139988
1	0	-3.951772	-0.151183	-2.396774
6	0	-4.776116	0.209542	-0.449636
1	0	-4.462925	0.117961	0.605812
1	0	-4.989952	1.277454	-0.641570
8	0	-5.879024	-0.618479	-0.765211
1	0	-6.612075	-0.363224	-0.193757

- Exo product:

6	0	-0.636913	3.153288	0.095342
6	0	-0.403477	1.675805	0.391094
6	0	1.543262	2.530125	0.230685
6	0	0.582629	3.688557	-0.002499
1	0	-1.610992	3.602454	-0.088588
1	0	0.863767	4.701770	-0.282953
6	0	0.073559	1.074282	-0.989782
1	0	-0.609816	1.335190	-1.802705
6	0	1.501159	1.640799	-1.054622
1	0	1.775438	2.177876	-1.969528
1	0	2.548012	2.737743	0.608859
8	0	0.810256	1.701496	1.127781
6	0	2.399993	0.430480	-0.852787
6	0	0.251057	-0.429960	-0.914178
7	0	1.604143	-0.713545	-0.834664
8	0	-0.624982	-1.268474	-0.903566
8	0	3.597013	0.436770	-0.714463
6	0	-1.477620	0.888782	1.096668
1	0	-1.772688	1.397871	2.032133
1	0	-1.071019	-0.106778	1.346857
8	0	-2.549269	0.792334	0.192964
6	0	-3.369774	-0.334341	0.403510
1	0	-3.985950	-0.221982	1.314976
1	0	-2.739304	-1.237328	0.509985
6	0	2.132934	-2.022122	-0.455924
1	0	3.193502	-1.828201	-0.244448
6	0	-4.261417	-0.485552	-0.815353
1	0	-3.616132	-0.560715	-1.708441
1	0	-4.890159	0.409060	-0.925986
8	0	-5.134234	-1.585322	-0.681108
1	0	-4.594182	-2.385595	-0.706660
6	0	1.455312	-2.500450	0.829392
1	0	0.452658	-2.910042	0.608429
1	0	2.072042	-3.307779	1.270876
6	0	1.989395	-3.038176	-1.581576
1	0	2.551279	-2.714286	-2.467535
1	0	2.384234	-4.013109	-1.260366
1	0	0.931071	-3.156640	-1.851732
8	0	1.357158	-1.404269	1.699845
6	0	0.780774	-1.743990	2.933809
1	0	1.396620	-2.482033	3.480066
1	0	0.713361	-0.821786	3.523996
1	0	-0.233509	-2.166319	2.801123
